# Carga de Doenças Cardiovasculares Atribuível aos Fatores de Risco nos Países de Língua Portuguesa: Dados do Estudo “Global Burden of Disease 2019”

**DOI:** 10.36660/abc.20210680

**Published:** 2022-06-06

**Authors:** Bruno Ramos Nascimento, Luisa Campos Caldeira Brant, André Dias Nassar Naback, Guilherme Augusto Veloso, Carisi Anne Polanczyk, Antonio Luiz Pinho Ribeiro, Deborah Carvalho Malta, Albano Vicente Lopes Ferreira, Gláucia Maria Moraes de Oliveira

**Affiliations:** 1 Universidade Federal de Minas Gerais Faculdade de Medicina e Hospital das Clínicas Belo Horizonte MG Brasil Faculdade de Medicina e Hospital das Clínicas, Universidade Federal de Minas Gerais, Belo Horizonte, MG – Brasil; 2 Universidade Federal de Minas Gerais Departamento de Estatística Programa de Pós-Graduação em Estatística Belo Horizonte MG Brasil Programa de Pós-Graduação em Estatística, Departamento de Estatística, Universidade Federal de Minas Gerais, Belo Horizonte, MG – Brasil; 3 Instituto Nacional de Avaliação de Tecnologias em Saúde Porto Alegre RS Brasil Instituto Nacional de Avaliação de Tecnologias em Saúde, IATS/CNPq, Porto Alegre, RS – Brasil; 4 Universidade Federal do Rio Grande do Sul Faculdade de Medicina Porto Alegre RS Brasil Faculdade de Medicina - Universidade Federal do Rio Grande do Sul, Porto Alegre, RS – Brasil; 5 Hospital Moinhos de Vento Porto Alegre RS Brasil Hospital Moinhos de Vento, Porto Alegre, RS – Brasil; 6 Universidade Federal de Minas Gerais Escola de Enfermagem Belo Horizonte MG Brasil Escola de Enfermagem, Universidade Federal de Minas Gerais, Belo Horizonte, MG – Brasil; 7 Universidade Katyavala Bwila Faculdade de Medicina Benguela Angola Faculdade de Medicina, Universidade Katyavala Bwila, Benguela – Angola; 8 Universidade Federal do Rio de Janeiro Rio de Janeiro RJ Brasil Universidade Federal do Rio de Janeiro, Rio de Janeiro, RJ – Brasil

**Keywords:** Doença Cardiovascular, Fatores de Risco, Carga Global da Doença, Epidemiologia, Comunidade dos Países de Língua Portuguesa

## Abstract

**Fundamento:**

O impacto dos fatores de risco (FR) sobre a morbimortalidade por doença cardiovascular (DCV) na maioria dos países de língua portuguesa (PLP) é pouco conhecido.

**Objetivo:**

Analisar a morbimortalidade por DCV atribuível aos FR e sua variação nos PLP de 1990 a 2019, a partir de estimativas do estudo Global Burden of Disease (GBD) 2019.

**Métodos:**

Avaliamos as mudanças nos FR ocorridas no período, as taxas de mortalidade e os anos de vida perdidos por incapacidade (DALYs), padronizados por idade, entre 1990 e 2019. Realizou-se a correlação entre a variação percentual das taxas de mortalidade e o índice sociodemográfico (SDI) de cada PLP pelo método de Spearman. O valor p<0,05 foi considerado estatisticamente significativo.

**Resultados:**

A pressão arterial sistólica (PAS) elevada foi o principal fator de risco para mortalidade e DALY por DCV para todos os PLP. A mortalidade por DCV mostrou uma tendência de redução em 2019, maior em Portugal (-66,6%, IC95% -71,0 - -61,2) e no Brasil (-49,8%, IC95% -52,5 - -47,1). Observou-se tendência à correlação inversa entre SDI e a variação percentual da mortalidade, que foi significativa para os riscos dietéticos (r=-0,70, p=0,036), colesterol LDL elevado (r=-0,77, p=0,015) e PAS elevada (r=-0,74, p=0,023).

**Conclusões:**

Além da PAS, os FR dietéticos e metabólicos justificaram uma maior variação da carga de DCV, correlacionada com o SDI nos PLP, sugerindo a necessidade de adoção de políticas de saúde adaptadas à realidade de cada país, visando a redução de seu impacto sobre a população.

## Introdução

As doenças cardiovasculares (DCVs) são as principais causas de morte no mundo, embora ainda não as sejam em muitos países de baixa e média renda, onde a transição epidemiológica ocorreu mais tardiamente.^1^ No entanto, com o controle das doenças infecciosas e materno-infantis, além do aumento da expectativa de vida e da urbanização, a importância das DCVs tende a crescer nesses países, demandando uma adaptação dos sistemas de saúde. Em muitos países, já se evidencia um aumento da proporção das DCV no total de mortes por todas as causas.^2,3^

Além disso, as DCVs têm impactado significativamente na morbidade, sendo importantes causas de incapacidade e, consequentemente, de perda de anos de vida saudáveis.^2,4^ Para se estabelecer estratégias de controle e prevenção das DCVs, é fundamental conhecer os principais fatores de risco (FR) cardiovasculares e suas prevalências. A hipertensão arterial e os fatores dietéticos continuam sendo os principais FR para DCV no mundo.^4,5^ Entretanto, nos últimos anos, outros fatores vêm exercendo um papel progressivamente maior no desenvolvimento das DCVs, o índice de massa corporal (IMC) elevado, elevação na glicemia de jejum e níveis séricos de LDL- colesterol, consumo de álcool e disfunção renal.^4^

Os países de língua portuguesa (PLP) sofreram influências culturais de Portugal em diferentes intensidades, sendo o tipo de colonização e os modelos político-econômicos importantes determinantes de sua heterogeneidade.^6^ Apesar de várias semelhanças socioculturais, são países com realidades socioeconômicas distintas, o que tem impacto direto sobre o padrão e as tendências temporais da carga de doenças. Dados apresentados em um estudo^3^ de tendências na morbimortalidade por DCVs mostraram diferenças na importância relativa da carga de DCV nesses países. No entanto, os FR atribuíveis mais relevantes para as DCVs (hipertensão arterial e os fatores dietéticos) são comuns entre a maioria dos PLP.^4^ A análise pormenorizada desses dados pode propiciar uma troca de informações entre os países no que concerne ações bem-sucedidas de enfrentamento às DCV, principalmente em relação ao controle dos principais FR e redução do seu impacto sobre a morbidade e mortalidade cardiovascular.

O “*Global Burden of Disease Study*” (GBD) é um importante estudo epidemiológico observacional que utiliza métricas de morbimortalidade relativas às principais doenças e fatores de risco em níveis global, nacional e regional. Um dos objetivos do GBD é compreender, por meio de avaliação de tendências, as mudanças no perfil das doenças que afetam as populações no século XXI, servindo também como um instrumento para tomada de decisão em políticas de saúde.^4,7^ O objetivo do presente estudo foi analisar a tendência dos FR cardiovasculares e a carga das DCVs atribuível a esses FR entre 1990 e 2019, nos PLP, a partir de estimativas do estudo GBD 2019 do *Institute of Health Metrics and Evaluation* (IHME).^8^

## Métodos

### Países de língua portuguesa

Os PLP são os membros oficiais da Comunidade dos Países de Língua Portuguesa: Angola, Brasil, Cabo Verde, Guiné-Bissau, Moçambique, Portugal e São Tomé e Príncipe, Timor-Leste e Guiné Equatorial.^6^ A Guiné Equatorial, originalmente uma colônia portuguesa, tem três línguas oficiais (espanhola, francesa, e portuguesa) e é o membro mais recente da Comunidade, desde 2014. Considerando-se a influência portuguesa – em diferentes magnitudes – sobre traços socioculturais, hábitos e comportamentos em saúde, e sobre a organização dos sistemas de saúde, contrastando com a heterogeneidade no desenvolvimento socioeconômico, consideramos relevante o estudo dos FR cardiovasculares no grupo de PLP.

### Estimativas de Carga atribuível e Exposição aos Fatores de Risco

O GBD utiliza uma lista hierárquica de FR que são analisados em quatro níveis. O nível 1 estratifica os FR em três grupos: metabólicos, comportamentais e ambientais. Os FR do nível 1 são detalhados no nível 2, perfazendo 20 FR. Os níveis 3 e 4 avançam nesse detalhamento, sendo que ao todo, em 2019, o estudo GBD analisou no total 87 FR.^4^ No estudo atual analisamos 12 FR, conforme Tabela 1. A opção por este grupo de FR deveu-se à sua associação epidemiológica mais robusta e bem estabelecida na literatura com a carga de doença e mortalidade por DCV, objetos deste estudo.

**Tabela 1:** Taxas de mortalidade padronizadas por idade

Países	Fatores de risco	Mulheres			Homens			Both		
		1990	2019	Variação percentual %	1990	2019	Variação percentual %	1990	2019	Variação percentual %
**Angola**	Todos os fatores de risco	287,8 (201,2 ; 369,9)	245,7 (200,3 ; 300,0)	-14,6 (-37,4 ; 21,5)	338,1 (257,8 ; 411,9)	274,3 (231,3 ; 327,8)	-18,9 (-37,2 ; 11,9)	314.4 (255.6 ; 375.7)	260.2 (219.5 ; 310.4)	-17.2 (-34.3 ; 6.1)
	Poluição do ar	94,2 ( 58,6 ; 133,9)	51,3 ( 36,5 ; 69,0)	-45,5 (-63,0 ; -16,2)	122,0 ( 91,7 ; 156,3)	66,0 ( 49,5 ; 85,3)	-45,9 (-60,8 ; -22,3)	108.4 ( 82.6 ; 140.8)	58.1 ( 43.1 ; 75.7)	-46.4 (-61.2 ; -27.3)
	Consumo de álcool	-0,1 ( -4,3 ; 4,2)	8,7 ( 2,4 ; 16,4)	-11607,2 (-5407,9 ; 5127,9)	4,5 ( -2,8 ; 13,0)	21,3 ( 12,4 ; 31,2)	367,8 (-3677,7 ; 4350,4)	2.2 ( -2.5 ; 6.9)	14.1 ( 8.4 ; 21.3)	555.0 (-4697.5 ; 4693.6)
	Riscos dietéticos	94,2 ( 59,0 ; 141,1)	74,6 ( 50,1 ; 112,6)	-20,8 (-44,1 ; 16,8)	126,7 ( 91,0 ; 174,1)	92,8 ( 67,3 ; 131,2)	-26,7 (-45,7 ; 3,1)	110.8 ( 81.0 ; 154.2)	83.2 ( 58.6 ; 120.5)	-24.9 (-43.4 ; -0.5)
	Índice de massa corporal elevado	19,2 ( 4,1 ; 46,4)	40,1 ( 19,8 ; 64,9)	108,5 ( 17,6 ; 551,7)	19,2 ( 3,7 ; 48,5)	38,0 ( 17,7 ; 62,7)	97,8 ( 12,6 ; 533,9)	19.4 ( 4.0 ; 47.3)	39.5 ( 19.6 ; 63.8)	103.6 ( 20.2 ; 517.9)
	Glicemia de jejum elevada	33,2 ( 17,6 ; 59,3)	42,4 ( 23,6 ; 70,1)	28,0 (-22,5 ; 116,4)	67,8 ( 41,9 ; 102,0)	79,6 ( 51,9 ; 115,9)	17,3 (-18,7 ; 85,7)	49.6 ( 32.2 ; 75.4)	58.4 ( 37.8 ; 87.3)	17.8 (-14.6 ; 71.0)
	Colesterol LDL elevado	40,9 ( 24,9 ; 63,3)	40,8 ( 25,0 ; 60,4)	-0,2 (-32,5 ; 46,7)	51,8 ( 35,4 ; 73,3)	46,8 ( 31,4 ; 66,4)	-9,7 (-34,4 ; 28,5)	46.8 ( 32.1 ; 66.3)	44.0 ( 28.6 ; 62.8)	-6.0 (-31.5 ; 26.1)
	Pressão arterial sistólica elevada	216,1 (153,1 ; 284,2)	188,7 (148,2 ; 236,2)	-12,7 (-37,4 ; 27,4)	230,7 (171,4 ; 291,4)	188,9 (154,6 ; 230,9)	-18,1 (-38,1 ; 15,3)	225.3 (178.2 ; 279.6)	191.0 (156.9 ; 233.6)	-15.2 (-33.8 ; 10.8)
	Disfunção renal	13,4 ( 8,2 ; 20,4)	15,0 ( 10,3 ; 20,8)	11,4 (-20,0 ; 64,3)	16,6 ( 11,2 ; 22,9)	16,7 ( 12,1 ; 22,3)	0,3 (-25,0 ; 39,7)	15.1 ( 10.4 ; 20.9)	15.9 ( 11.3 ; 21.6)	5.1 (-20.1 ; 37.9)
	Baixo nível de atividade física	8,0 ( 3,1 ; 16,6)	9,0 ( 3,8 ; 18,0)	12,8 (-20,7 ; 67,8)	5,2 ( 1,4 ; 13,0)	5,7 ( 1,8 ; 14,1)	10,4 (-20,5 ; 64,7)	6.8 ( 2.6 ; 15.1)	7.8 ( 3.0 ; 16.4)	14.3 (-16.9 ; 56.3)
	Temperatura não ideal	11,1 ( 5,3 ; 17,9)	8,8 ( 5,1 ; 13,4)	-20,9 (-45,7 ; 31,0)	12,8 ( 6,8 ; 20,5)	9,5 ( 5,7 ; 14,9)	-25,6 (-45,0 ; 10,4)	12.0 ( 6.2 ; 18.6)	9.2 ( 5.5 ; 14.1)	-23.6 (-42.7 ; 11.3)
	Outros riscos ambientais	12,3 ( 4,7 ; 23,2)	11,6 ( 5,4 ; 19,7)	-6,2 (-32,9 ; 45,1)	20,2 ( 10,9 ; 31,6)	17,2 ( 10,1 ; 25,6)	-14,8 (-35,6 ; 19,2)	16.2 ( 8.1 ; 26.6)	14.0 ( 7.6 ; 21.8)	-13.4 (-31.9 ; 13.9)
	Tabagismo	13,1 ( 8,6 ; 18,3)	10,9 ( 7,9 ; 14,6)	-16,4 (-44,1 ; 28,2)	58,3 ( 43,6 ; 73,5)	43,8 ( 34,9 ; 55,4)	-24,9 (-45,5 ; 6,2)	35.4 ( 27.6 ; 43.9)	25.6 ( 20.2 ; 32.5)	-27.9 (-46.2 ; -1.5)
**Brasil**	Todos os fatores de risco	259,5 (237,7 ; 274,8)	118,2 (103,9 ; 128,2)	-54,4 (-57,0 ; -52,2)	352,0 (331,8 ; 368,2)	175,8 (161,3 ; 187,3)	-50,0 (-53,2 ; -47,4)	303.1 (282.6 ; 317.6)	144.3 (130.8 ; 153.5)	-52.4 (-54.5 ; -50.6)
	Poluição do ar	46,3 ( 34,4 ; 60,2)	10,8 ( 7,8 ; 13,9)	-76,7 (-83,5 ; -67,8)	65,0 ( 46,7 ; 84,7)	16,6 ( 11,9 ; 21,8)	-74,5 (-82,1 ; -64,2)	55.1 ( 40.4 ; 71.9)	13.4 ( 9.8 ; 17.6)	-75.6 (-82.9 ; -66.0)
	Consumo de álcool	-0,1 ( -1,7 ; 1,6)	0,2 ( -0,6 ; 1,2)	-315,7 (-576,3 ; 511,4)	11,5 ( 5,8 ; 18,0)	6,5 ( 3,2 ; 9,9)	-43,7 (-60,0 ; -19,6)	5.3 ( 2.4 ; 8.5)	3.0 ( 1.4 ; 4.8)	-43.5 (-62.5 ; -11.6)
	Riscos dietéticos	95,1 ( 74,8 ; 121,0)	38,4 ( 29,3 ; 50,7)	-59,6 (-63,8 ; -55,9)	144,9 (115,3 ; 181,6)	65,7 ( 50,4 ; 84,1)	-54,7 (-59,0 ; -50,5)	118.4 ( 94.6 ; 148.9)	50.7 ( 39.2 ; 65.7)	-57.2 (-60.9 ; -53.9)
	Índice de massa corporal elevado	54,7 ( 32,2 ; 81,1)	36,2 ( 25,4 ; 48,4)	-33,9 (-43,7 ; -16,7)	62,1 ( 32,4 ; 98,6)	47,9 ( 30,4 ; 66,8)	-22,8 (-35,9 ; 6,2)	58.5 ( 32.7 ; 89.7)	41.8 ( 28.1 ; 56.8)	-28.5 (-38.8 ; -8.6)
	Glicemia de jejum elevada	58,1 ( 38,4 ; 88,3)	27,2 ( 18,0 ; 41,6)	-53,2 (-59,3 ; -46,1)	85,4 ( 58,1 ; 128,3)	47,1 ( 32,1 ; 68,6)	-44,9 (-50,4 ; -37,8)	70.4 ( 47.4 ; 106.1)	35.9 ( 24.5 ; 53.0)	-49.0 (-53.4 ; -43.9)
	Colesterol LDL elevado	72,9 ( 54,2 ; 97,8)	33,8 ( 25,0 ; 45,1)	-53,7 (-56,9 ; -50,4)	105,7 ( 82,4 ; 133,6)	54,2 ( 42,3 ; 68,4)	-48,8 (-52,1 ; -44,9)	88.6 ( 67.8 ; 114.8)	43.1 ( 33.4 ; 55.9)	-51.3 (-53.8 ; -48.6)
	Pressão arterial sistólica elevada	161,6 (140,1 ; 182,2)	76,8 ( 64,8 ; 87,4)	-52,5 (-56,0 ; -49,0)	212,6 (187,4 ; 236,0)	113,0 ( 98,4 ; 126,1)	-46,8 (-50,3 ; -43,3)	186.1 (163.8 ; 206.7)	93.4 ( 80.2 ; 104.2)	-49.8 (-52.5 ; -47.1)
	Disfunção renal	21,9 ( 16,9 ; 27,2)	11,0 ( 8,6 ; 13,7)	-49,7 (-53,4 ; -46,4)	29,3 ( 23,1 ; 35,8)	16,8 ( 13,2 ; 20,5)	-42,9 (-47,0 ; -38,5)	25.5 ( 19.9 ; 31.3)	13.6 ( 10.8 ; 16.7)	-46.5 (-49.6 ; -43.4)
	Baixo nível de atividade física	25,1 ( 13,1 ; 38,0)	12,3 ( 7,4 ; 17,9)	-50,9 (-56,7 ; -39,7)	27,0 ( 11,4 ; 45,7)	15,4 ( 7,9 ; 24,5)	-42,9 (-50,3 ; -24,9)	26.1 ( 12.6 ; 41.4)	13.7 ( 7.6 ; 20.8)	-47.6 (-53.6 ; -35.0)
	Temperatura não ideal	8,7 ( 2,0 ; 13,7)	3,1 ( 0,9 ; 4,8)	-64,0 (-78,6 ; -27,2)	11,0 ( 1,4 ; 17,5)	4,4 ( 0,9 ; 6,7)	-60,3 (-83,9 ; -5,5)	9.8 ( 1.8 ; 15.4)	3.7 ( 0.8 ; 5.6)	-62.3 (-78.6 ; -19.0)
	Outros riscos ambientais	8,7 ( 3,3 ; 14,5)	3,9 ( 1,5 ; 6,7)	-55,7 (-59,4 ; -51,1)	17,5 ( 9,7 ; 25,5)	7,8 ( 4,1 ; 11,9)	-55,3 (-60,2 ; -51,4)	12.7 ( 6.2 ; 19.5)	5.6 ( 2.6 ; 8.9)	-56.1 (-60.0 ; -52.8)
	Tabagismo	68,4 ( 60,9 ; 76,3)	19,9 ( 17,9 ; 22,1)	-70,8 (-74,3 ; -67,1)	115,8 (108,7 ; 122,4)	36,9 ( 33,8 ; 39,7)	-68,1 (-70,7 ; -65,5)	90.6 ( 84.6 ; 96.5)	27.6 ( 25.5 ; 29.7)	-69.5 (-72.0 ; -67.1)
**Cabo Verde**	Todos os fatores de risco	163,7 (142,5 ; 184,5)	184,7 (152,9 ; 212,8)	12,8 ( -5,0 ; 33,2)	233,8 (204,9 ; 257,4)	274,6 (245,8 ; 303,6)	17,4 ( 3,5 ; 34,8)	192.1 (171.0 ; 209.8)	222.6 (193.8 ; 247.8)	15.9 ( 1.6 ; 32.0)
	Poluição do ar	51,0 ( 41,4 ; 62,5)	44,5 ( 34,8 ; 55,0)	-12,7 (-32,8 ; 13,7)	79,1 ( 66,8 ; 94,3)	75,6 ( 60,3 ; 90,9)	-4,4 (-23,6 ; 16,4)	62.5 ( 52.7 ; 74.6)	57.8 ( 46.6 ; 69.3)	-7.5 (-27.0 ; 15.3)
	Consumo de álcool	1,5 ( -1,0 ; 4,5)	1,9 ( -1,2 ; 5,5)	22,7 (-692,9 ; 1213,6)	6,2 ( 1,4 ; 11,2)	11,9 ( 5,4 ; 19,6)	93,5 ( -1,3 ; 463,0)	3.5 ( 0.7 ; 6.5)	6.1 ( 2.1 ; 10.8)	75.1 (-33.1 ; 490.7)
	Riscos dietéticos	62,9 ( 48,6 ; 82,7)	64,1 ( 46,1 ; 89,3)	1,8 (-16,2 ; 22,4)	105,5 ( 82,9 ; 133,2)	101,1 ( 76,7 ; 134,8)	-4,2 (-18,4 ; 13,1)	80.1 ( 63.1 ; 101.9)	79.7 ( 59.4 ; 108.0)	-0.5 (-15.6 ; 15.9)
	Índice de massa corporal elevado	24,3 ( 11,8 ; 39,0)	41,4 ( 26,5 ; 60,0)	70,3 ( 24,0 ; 174,8)	20,8 ( 7,1 ; 38,9)	53,3 ( 32,3 ; 79,4)	156,0 ( 80,8 ; 427,3)	22.8 ( 9.8 ; 38.8)	47.1 ( 29.7 ; 68.4)	107.1 ( 55.0 ; 249.1)
	Glicemia de jejum elevada	26,7 ( 15,4 ; 45,0)	59,1 ( 34,8 ; 92,6)	121,1 ( 57,8 ; 219,5)	41,5 ( 24,8 ; 69,3)	85,3 ( 54,4 ; 125,9)	105,5 ( 47,8 ; 189,0)	32.6 ( 19.4 ; 53.9)	69.5 ( 43.1 ; 106.5)	113.3 ( 61.1 ; 187.2)
	Colesterol LDL elevado	34,9 ( 23,2 ; 48,0)	43,4 ( 28,2 ; 61,3)	24,3 ( 2,9 ; 49,5)	56,5 ( 39,2 ; 76,3)	61,0 ( 41,8 ; 82,9)	8,0 ( -7,1 ; 28,2)	43.5 ( 30.0 ; 59.2)	51.1 ( 34.7 ; 69.9)	17.4 ( 1.0 ; 35.2)
	Pressão arterial sistólica elevada	111,5 ( 90,3 ; 134,6)	127,6 ( 98,1 ; 155,9)	14,4 (-10,7 ; 44,5)	149,7 (123,5 ; 177,1)	186,9 (156,5 ; 219,0)	24,9 ( 5,9 ; 48,5)	127.0 (106.9 ; 148.8)	153.2 (125.6 ; 179.6)	20.7 ( 2.9 ; 41.8)
	Disfunção renal	11,2 ( 7,7 ; 15,1)	17,7 ( 12,3 ; 23,4)	58,4 ( 29,2 ; 96,6)	16,4 ( 10,9 ; 22,3)	24,4 ( 17,4 ; 31,7)	49,0 ( 26,4 ; 79,6)	13.3 ( 9.0 ; 17.9)	20.5 ( 14.7 ; 26.8)	54.6 ( 31.5 ; 85.0)
	Baixo nível de atividade física	4,7 ( 1,7 ; 10,2)	6,7 ( 2,6 ; 14,1)	43,6 ( 16,1 ; 85,9)	5,5 ( 1,5 ; 14,5)	7,0 ( 2,1 ; 17,0)	26,3 ( -1,4 ; 74,0)	5.0 ( 1.7 ; 11.8)	6.9 ( 2.5 ; 15.9)	37.5 ( 13.5 ; 77.5)
	Temperatura não ideal	7,5 ( -0,0 ; 15,6)	7,7 ( 2,0 ; 16,1)	2,3 (-33,1 ; 63,5)	11,0 ( 0,6 ; 22,5)	11,0 ( 2,5 ; 23,3)	0,0 (-39,7 ; 68,0)	8.9 ( 0.2 ; 18.3)	9.1 ( 2.2 ; 19.1)	1.9 (-31.6 ; 66.1)
	Outros riscos ambientais	4,5 ( 1,1 ; 8,3)	4,8 ( 1,3 ; 8,7)	6,3 (-13,9 ; 42,5)	7,5 ( 2,6 ; 12,9)	8,0 ( 2,8 ; 14,0)	7,0 (-10,4 ; 27,1)	5.7 ( 1.7 ; 10.1)	6.1 ( 2.0 ; 10.9)	6.7 ( -8.7 ; 27.0)
	Tabagismo	10,6 ( 8,3 ; 13,1)	7,8 ( 6,1 ; 9,7)	-25,9 (-44,1 ; -2,7)	37,4 ( 32,5 ; 42,7)	28,9 ( 24,6 ; 33,5)	-22,9 (-35,7 ; -7,0)	21.8 ( 19.1 ; 24.5)	16.9 ( 14.5 ; 19.8)	-22.2 (-34.8 ; -6.2)
**Guiné Equatorial**	Todos os fatores de risco	312,2 (195,9 ; 439,0)	231,1 (163,4 ; 304,0)	-26,0 (-55,7 ; 25,7)	401,8 (299,2 ; 494,7)	211,0 (155,6 ; 269,7)	-47,5 (-61,9 ; -21,2)	354.2 (266.7 ; 450.9)	224.4 (171.1 ; 285.6)	-36.6 (-56.7 ; -10.7)
	Poluição do ar	110,1 ( 61,0 ; 175,4)	47,7 ( 28,7 ; 70,1)	-56,7 (-77,8 ; -18,4)	155,9 (112,9 ; 206,2)	49,0 ( 30,6 ; 70,4)	-68,6 (-81,7 ; -48,4)	130.9 ( 92.0 ; 181.6)	48.4 ( 30.5 ; 69.4)	-63.0 (-79.1 ; -41.8)
	Consumo de álcool	0,9 ( -4,5 ; 7,1)	6,4 ( -0,2 ; 15,1)	607,8 (-3342,7 ; 3175,8)	7,6 ( -3,2 ; 19,9)	13,7 ( 5,2 ; 23,5)	80,7 (-1716,7 ; 1479,2)	3.8 ( -3.0 ; 11.4)	9.3 ( 2.6 ; 17.0)	148.5 (-2409.6 ; 2496.0)
	Riscos dietéticos	97,6 ( 54,9 ; 161,4)	65,3 ( 38,5 ; 104,8)	-33,1 (-59,7 ; 15,0)	146,3 (100,8 ; 204,3)	65,3 ( 42,2 ; 98,3)	-55,4 (-69,0 ; -33,0)	119.6 ( 82.7 ; 171.8)	65.7 ( 41.8 ; 101.7)	-45.0 (-62.8 ; -20.8)
	Índice de massa corporal elevado	27,2 ( 7,2 ; 59,7)	68,2 ( 40,0 ; 104,7)	150,9 ( 20,1 ; 665,3)	24,2 ( 4,8 ; 59,4)	55,5 ( 31,8 ; 86,2)	128,9 ( 9,4 ; 813,1)	26.1 ( 6.3 ; 58.3)	63.5 ( 38.5 ; 97.1)	143.0 ( 18.3 ; 670.7)
	Glicemia de jejum elevada	33,9 ( 17,0 ; 60,7)	44,1 ( 25,0 ; 74,9)	30,0 (-29,6 ; 140,1)	74,7 ( 44,3 ; 116,1)	63,3 ( 39,1 ; 97,4)	-15,2 (-44,9 ; 36,4)	51.0 ( 32.5 ; 77.6)	51.8 ( 31.1 ; 80.7)	1.5 (-35.4 ; 50.4)
	Colesterol LDL elevado	45,1 ( 23,6 ; 74,6)	39,9 ( 21,4 ; 61,7)	-11,6 (-48,6 ; 55,4)	63,2 ( 42,2 ; 89,2)	36,1 ( 22,2 ; 54,1)	-42,9 (-60,6 ; -11,8)	53.8 ( 34.5 ; 78.9)	38.7 ( 22.3 ; 59.0)	-28.0 (-54.0 ; 5.2)
	Pressão arterial sistólica elevada	232,7 (145,3 ; 329,7)	179,8 (124,1 ; 241,0)	-22,7 (-55,2 ; 33,7)	273,3 (195,5 ; 347,1)	149,3 (105,4 ; 194,7)	-45,4 (-61,3 ; -17,6)	253.5 (187.1 ; 327.0)	168.9 (124.5 ; 218.2)	-33.4 (-54.9 ; -3.9)
	Disfunção renal	14,7 ( 8,0 ; 23,8)	16,2 ( 10,0 ; 24,0)	10,2 (-35,3 ; 95,5)	20,0 ( 13,5 ; 27,8)	14,0 ( 9,3 ; 19,5)	-30,3 (-50,9 ; 3,2)	17.2 ( 11.4 ; 24.7)	15.4 ( 10.1 ; 21.8)	-10.3 (-40.9 ; 29.9)
	Baixo nível de atividade física	8,6 ( 3,0 ; 19,0)	10,5 ( 4,4 ; 20,2)	21,1 (-27,8 ; 119,4)	6,1 ( 1,7 ; 15,4)	5,9 ( 1,9 ; 13,5)	-3,8 (-38,6 ; 63,1)	7.8 ( 2.8 ; 16.9)	8.8 ( 3.5 ; 17.4)	12.0 (-28.4 ; 76.1)
	Temperatura não ideal	7,4 ( -1,1 ; 18,0)	4,0 ( 0,6 ; 9,8)	-45,8 (-78,1 ; 9,3)	9,4 ( -0,2 ; 22,5)	3,5 ( 0,5 ; 8,9)	-62,6 (-95,2 ; -30,8)	8.4 ( -0.8 ; 19.5)	3.9 ( 0.6 ; 9.6)	-54.0 (-78.8 ; -13.0)
	Outros riscos ambientais	19,6 ( 9,4 ; 35,3)	12,8 ( 6,5 ; 21,1)	-35,1 (-61,2 ; 8,7)	35,5 ( 21,3 ; 51,7)	15,2 ( 8,9 ; 22,8)	-57,4 (-69,5 ; -38,5)	26.6 ( 15.7 ; 40.7)	13.7 ( 7.7 ; 21.3)	-48.4 (-64.0 ; -28.0)
	Tabagismo	10,3 ( 6,1 ; 16,0)	6,2 ( 3,9 ; 9,2)	-40,0 (-65,6 ; 7,2)	66,3 ( 47,2 ; 86,9)	27,3 ( 18,5 ; 38,1)	-58,9 (-72,9 ; -36,6)	35.0 ( 25.9 ; 45.4)	14.9 ( 10.3 ; 20.7)	-57.4 (-71.3 ; -36.8)
**Guiné-Bissau**	Todos os fatores de risco	284,9 (210,9 ; 356,1)	300,9 (236,2 ; 378,4)	5,6 (-22,2 ; 46,2)	377,0 (298,8 ; 461,9)	341,4 (278,3 ; 408,3)	-9,5 (-30,7 ; 19,7)	329.8 (270.3 ; 392.1)	320.6 (258.7 ; 395.1)	-2.8 (-24.5 ; 26.0)
	Poluição do ar	116,1 ( 78,7 ; 161,0)	103,8 ( 77,5 ; 134,4)	-10,6 (-35,2 ; 27,9)	161,3 (116,7 ; 216,6)	125,4 ( 98,1 ; 154,8)	-22,3 (-42,2 ; 6,5)	138.2 (103.2 ; 182.1)	114.0 ( 88.8 ; 142.8)	-17.5 (-37.7 ; 11.4)
	Consumo de álcool	2,6 ( -1,4 ; 7,3)	2,1 ( -1,8 ; 7,3)	-17,6 (-567,9 ; 611,3)	14,2 ( 5,2 ; 24,2)	11,8 ( 3,7 ; 21,9)	-16,7 (-68,9 ; 85,5)	8.2 ( 2.7 ; 14.4)	6.4 ( 1.4 ; 12.7)	-20.9 (-81.3 ; 97.5)
	Riscos dietéticos	107,8 ( 71,4 ; 153,9)	111,0 ( 77,2 ; 162,2)	3,0 (-25,6 ; 45,6)	165,2 (118,0 ; 222,6)	141,1 (103,9 ; 192,1)	-14,6 (-35,6 ; 15,1)	135.1 ( 98.5 ; 186.1)	124.6 ( 90.2 ; 173.8)	-7.8 (-29.8 ; 21.3)
	Índice de massa corporal elevado	36,3 ( 14,0 ; 67,4)	58,1 ( 30,4 ; 92,6)	59,7 ( 7,6 ; 191,7)	27,2 ( 7,6 ; 59,8)	41,7 ( 16,3 ; 74,4)	53,5 ( -0,2 ; 216,2)	32.0 ( 11.2 ; 62.9)	51.0 ( 25.4 ; 85.8)	59.3 ( 11.3 ; 181.6)
	Glicemia de jejum elevada	33,0 ( 19,5 ; 55,8)	68,8 ( 41,2 ; 109,3)	108,5 ( 33,7 ; 235,3)	51,1 ( 31,1 ; 84,5)	81,5 ( 48,9 ; 127,5)	59,5 ( 7,0 ; 145,4)	41.5 ( 26.5 ; 65.9)	74.0 ( 46.3 ; 114.7)	78.4 ( 25.1 ; 152.6)
	Colesterol LDL elevado	48,5 ( 30,5 ; 74,6)	58,8 ( 38,4 ; 84,2)	21,5 (-16,1 ; 76,4)	68,1 ( 45,1 ; 97,7)	69,3 ( 47,8 ; 94,4)	1,7 (-25,0 ; 41,2)	57.8 ( 39.1 ; 82.7)	63.9 ( 42.8 ; 88.4)	10.4 (-17.9 ; 50.7)
	Pressão arterial sistólica elevada	194,4 (137,7 ; 255,5)	212,7 (159,7 ; 275,9)	9,4 (-21,2 ; 57,1)	233,4 (176,8 ; 295,8)	225,7 (174,4 ; 280,9)	-3,3 (-28,5 ; 32,7)	214.0 (168.0 ; 264.6)	220.4 (171.5 ; 277.2)	3.0 (-22.5 ; 35.5)
	Disfunção renal	19,2 ( 13,0 ; 26,7)	24,2 ( 17,2 ; 33,5)	26,2 ( -8,1 ; 78,2)	24,4 ( 16,6 ; 33,8)	25,9 ( 18,2 ; 35,0)	6,2 (-19,7 ; 42,9)	21.7 ( 15.6 ; 29.0)	25.1 ( 18.3 ; 34.1)	15.5 (-11.5 ; 52.2)
	Baixo nível de atividade física	6,3 ( 2,3 ; 14,0)	7,5 ( 2,9 ; 16,4)	20,3 (-16,3 ; 73,0)	8,1 ( 2,2 ; 20,4)	7,8 ( 2,3 ; 19,1)	-2,8 (-26,5 ; 35,7)	7.1 ( 2.4 ; 16.7)	7.7 ( 2.8 ; 17.5)	8.4 (-17.2 ; 44.5)
	Temperatura não ideal	8,4 (-23,0 ; 18,2)	9,5 ( 2,5 ; 17,7)	13,1 (-159,3 ; 104,1)	11,5 (-34,1 ; 23,8)	10,8 ( 1,1 ; 18,2)	-5,8 (-159,8 ; 47,8)	9.9 (-27.3 ; 20.4)	10.1 ( 1.7 ; 17.8)	2.4 (-163.6 ; 60.8)
	Outros riscos ambientais	12,5 ( 5,3 ; 21,2)	14,7 ( 7,2 ; 23,9)	17,3 (-14,5 ; 71,4)	23,1 ( 12,9 ; 35,0)	22,4 ( 13,6 ; 32,9)	-2,8 (-27,3 ; 32,9)	17.6 ( 9.4 ; 26.9)	18.1 ( 10.0 ; 27.6)	3.0 (-21.8 ; 37.3)
	Tabagismo	12,2 ( 8,6 ; 16,4)	10,9 ( 7,9 ; 14,5)	-10,7 (-40,0 ; 29,7)	48,9 ( 36,7 ; 64,1)	32,8 ( 25,4 ; 40,7)	-33,0 (-52,6 ; -5,2)	29.9 ( 23.1 ; 38.5)	20.9 ( 16.2 ; 26.4)	-30.3 (-50.1 ; -4.3)
**Moçambique**	Todos os fatores de risco	251,3 (203,0 ; 301,4)	247,6 (192,6 ; 324,9)	-1,5 (-26,5 ; 31,6)	288,1 (234,5 ; 345,8)	370,1 (311,7 ; 432,8)	28,4 ( 2,6 ; 61,0)	270.8 (228.0 ; 315.3)	304.8 (246.3 ; 373.7)	12.6 (-11.7 ; 41.3)
	Poluição do ar	96,2 ( 68,5 ; 136,1)	79,0 ( 57,2 ; 109,2)	-17,9 (-41,6 ; 17,1)	119,3 ( 88,9 ; 165,1)	134,1 (107,1 ; 163,5)	12,4 (-16,9 ; 49,3)	107.7 ( 80.7 ; 146.4)	104.2 ( 80.7 ; 133.0)	-3.3 (-28.7 ; 29.2)
	Consumo de álcool	-1,0 ( -2,4 ; 0,5)	-0,8 ( -3,5 ; 2,0)	-26,8 (-844,2 ; 623,6)	-0,8 ( -5,3 ; 3,8)	3,4 ( -6,0 ; 13,8)	-507,8 (-3041,8 ; 2468,5)	-0.9 ( -3.3 ; 1.5)	1.2 ( -3.5 ; 6.3)	-226.5 (-3065.6 ; 2361.7)
	Riscos dietéticos	94,9 ( 56,2 ; 147,7)	85,8 ( 48,2 ; 140,9)	-9,6 (-35,2 ; 22,8)	123,1 ( 80,9 ; 180,3)	135,1 ( 91,4 ; 192,8)	9,8 (-18,0 ; 43,2)	108.7 ( 70.0 ; 161.6)	108.4 ( 68.2 ; 163.7)	-0.3 (-23.8 ; 28.3)
	Índice de massa corporal elevado	17,8 ( 4,5 ; 40,2)	41,5 ( 20,3 ; 70,2)	132,9 ( 35,3 ; 494,2)	15,5 ( 3,0 ; 38,3)	49,7 ( 21,6 ; 84,9)	219,9 ( 89,5 ; 870,9)	16.9 ( 3.9 ; 39.9)	46.1 ( 21.2 ; 77.5)	172.9 ( 63.8 ; 609.2)
	Glicemia de jejum elevada	23,7 ( 14,2 ; 41,7)	34,7 ( 18,7 ; 61,9)	46,0 (-20,0 ; 145,4)	43,0 ( 25,6 ; 67,2)	89,3 ( 57,9 ; 132,1)	107,8 ( 45,6 ; 204,7)	32.2 ( 20.1 ; 50.4)	57.0 ( 36.5 ; 87.5)	76.9 ( 25.5 ; 151.2)
	Colesterol LDL elevado	30,0 ( 19,1 ; 45,8)	35,1 ( 20,1 ; 54,2)	16,9 (-18,9 ; 61,2)	41,1 ( 28,1 ; 61,4)	62,4 ( 43,1 ; 88,1)	52,0 ( 14,1 ; 104,3)	35.4 ( 24.3 ; 51.8)	47.4 ( 31.1 ; 69.0)	34.0 ( 2.8 ; 74.7)
	Pressão arterial sistólica elevada	180,7 (139,6 ; 229,1)	185,7 (138,5 ; 247,4)	2,8 (-25,6 ; 42,5)	193,1 (149,7 ; 235,2)	264,3 (213,7 ; 319,3)	36,9 ( 5,2 ; 75,0)	188.8 (153.3 ; 228.7)	224.1 (177.4 ; 281.8)	18.7 ( -9.1 ; 51.3)
	Disfunção renal	11,8 ( 8,5 ; 16,3)	14,7 ( 10,1 ; 20,8)	24,3 (-10,0 ; 69,5)	14,8 ( 10,8 ; 20,1)	23,6 ( 17,7 ; 31,1)	59,8 ( 23,9 ; 103,7)	13.3 ( 9.8 ; 17.8)	18.8 ( 13.8 ; 25.3)	41.4 ( 8.8 ; 79.9)
	Baixo nível de atividade física	2,0 ( 0,7 ; 5,1)	2,4 ( 0,8 ; 6,2)	18,0 (-20,2 ; 70,2)	2,1 ( 0,7 ; 5,7)	3,2 ( 1,0 ; 8,7)	52,3 ( 9,0 ; 106,7)	2.1 ( 0.7 ; 5.4)	2.8 ( 1.0 ; 7.3)	33.0 ( -2.5 ; 77.4)
	Temperatura não ideal	8,2 ( 3,9 ; 13,0)	7,6 ( 4,2 ; 12,1)	-7,5 (-34,6 ; 39,0)	9,4 ( 4,7 ; 15,2)	11,1 ( 6,3 ; 17,5)	18,3 (-11,8 ; 70,2)	8.8 ( 4.3 ; 14.1)	9.2 ( 5.2 ; 14.7)	4.6 (-22.7 ; 52.5)
	Outros riscos ambientais	13,7 ( 6,6 ; 23,4)	14,3 ( 7,7 ; 23,9)	5,0 (-21,6 ; 43,2)	32,9 ( 22,7 ; 46,2)	36,4 ( 25,1 ; 49,3)	10,6 (-12,4 ; 38,7)	22.5 ( 14.4 ; 33.7)	23.4 ( 14.8 ; 34.6)	4.0 (-18.1 ; 30.6)
	Tabagismo	11,4 ( 8,3 ; 15,5)	10,6 ( 7,3 ; 14,8)	-7,7 (-40,2 ; 38,0)	43,8 ( 33,7 ; 55,6)	50,6 ( 39,6 ; 63,5)	15,4 (-13,7 ; 55,1)	26.7 ( 21.3 ; 32.6)	28.2 ( 21.9 ; 35.4)	5.4 (-21.1 ; 41.0)
**Portugal**	Todos os fatores de risco	255,9 (229,2 ; 276,1)	83,8 ( 71,0 ; 93,8)	-67,3 (-70,3 ; -64,6)	358,4 (336,7 ; 378,3)	124,2 (112,3 ; 133,7)	-65,3 (-67,6 ; -63,2)	299.9 (275.5 ; 319.2)	102.0 ( 89.8 ; 111.5)	-66.0 (-68.3 ; -63.9)
	Poluição do ar	21,3 ( 7,7 ; 38,2)	3,1 ( 1,8 ; 4,6)	-85,3 (-92,6 ; -64,5)	30,6 ( 10,1 ; 55,6)	5,2 ( 3,0 ; 7,6)	-82,9 (-91,5 ; -56,4)	25.4 ( 8.8 ; 46.0)	4.1 ( 2.4 ; 5.9)	-83.9 (-91.9 ; -60.0)
	Consumo de álcool	7,4 ( 1,9 ; 13,3)	1,7 ( 0,4 ; 3,3)	-76,7 (-90,7 ; -55,8)	42,1 ( 26,6 ; 57,0)	12,1 ( 7,4 ; 16,9)	-71,3 (-77,0 ; -65,3)	20.7 ( 14.0 ; 27.6)	5.9 ( 3.8 ; 8.2)	-71.4 (-77.9 ; -64.1)
	Riscos dietéticos	81,0 ( 66,4 ; 97,7)	26,4 ( 20,8 ; 33,1)	-67,5 (-70,7 ; -63,7)	123,9 (102,0 ; 150,3)	45,5 ( 36,5 ; 56,7)	-63,3 (-66,5 ; -59,8)	99.7 ( 82.6 ; 120.2)	34.9 ( 28.2 ; 43.5)	-65.0 (-67.7 ; -61.7)
	Índice de massa corporal elevado	38,0 ( 20,3 ; 58,4)	15,8 ( 9,3 ; 23,6)	-58,5 (-65,5 ; -45,3)	47,7 ( 21,5 ; 78,0)	22,1 ( 11,5 ; 34,2)	-53,6 (-60,3 ; -38,4)	42.8 ( 21.6 ; 67.6)	18.9 ( 10.6 ; 28.7)	-55.9 (-61.9 ; -42.4)
	Glicemia de jejum elevada	57,2 ( 34,6 ; 99,7)	26,7 ( 16,3 ; 43,3)	-53,3 (-68,0 ; -34,2)	72,3 ( 47,1 ; 118,4)	40,5 ( 27,1 ; 60,9)	-44,0 (-59,2 ; -25,7)	64.0 ( 40.9 ; 103.3)	32.7 ( 21.3 ; 51.3)	-48.9 (-61.3 ; -33.1)
	Colesterol LDL elevado	76,8 ( 48,5 ; 118,7)	23,8 ( 14,8 ; 36,1)	-69,0 (-72,4 ; -65,3)	112,6 ( 79,6 ; 160,9)	38,1 ( 27,7 ; 51,6)	-66,2 (-69,6 ; -62,6)	92.5 ( 62.4 ; 137.0)	30.3 ( 20.8 ; 43.1)	-67.2 (-70.3 ; -63.8)
	Pressão arterial sistólica elevada	152,8 (116,8 ; 187,4)	48,7 ( 37,7 ; 59,6)	-68,2 (-74,7 ; -60,0)	212,5 (177,4 ; 248,0)	73,0 ( 61,9 ; 85,3)	-65,7 (-70,3 ; -60,7)	179.0 (147.4 ; 210.6)	59.8 ( 49.6 ; 70.1)	-66.6 (-71.0 ; -61.2)
	Disfunção renal	23,7 ( 16,9 ; 30,3)	8,2 ( 5,7 ; 10,6)	-65,6 (-69,3 ; -61,9)	28,9 ( 22,0 ; 35,8)	10,2 ( 7,7 ; 12,8)	-64,6 (-67,6 ; -61,4)	26.1 ( 19.3 ; 32.8)	9.1 ( 6.7 ; 11.6)	-65.0 (-68.1 ; -61.8)
	Baixo nível de atividade física	20,0 ( 8,2 ; 36,7)	6,8 ( 2,9 ; 11,9)	-66,2 (-72,1 ; -57,6)	20,0 ( 6,2 ; 41,6)	7,2 ( 2,4 ; 13,7)	-64,2 (-71,1 ; -52,8)	20.4 ( 7.8 ; 38.8)	7.1 ( 2.8 ; 12.7)	-65.4 (-70.9 ; -57.5)
	Temperatura não ideal	29,1 ( 23,8 ; 34,7)	8,9 ( 7,1 ; 10,8)	-69,4 (-72,1 ; -67,3)	38,5 ( 31,6 ; 45,9)	12,3 ( 10,0 ; 14,8)	-68,1 (-70,1 ; -66,1)	33.1 ( 27.2 ; 39.5)	10.4 ( 8.4 ; 12.5)	-68.5 (-70.6 ; -66.6)
	Outros riscos ambientais	10,4 ( 5,0 ; 16,0)	3,4 ( 1,6 ; 5,4)	-67,5 (-72,3 ; -62,0)	23,6 ( 15,4 ; 32,3)	7,4 ( 4,6 ; 10,5)	-68,5 (-72,4 ; -65,1)	15.7 ( 9.3 ; 22.3)	5.0 ( 2.9 ; 7.4)	-68.0 (-71.7 ; -64.3)
	Tabagismo	26,6 ( 23,2 ; 30,4)	6,0 ( 5,2 ; 6,8)	-77,6 (-80,8 ; -74,1)	87,2 ( 81,5 ; 93,1)	24,0 ( 22,2 ; 25,9)	-72,5 (-74,8 ; -70,2)	52.2 ( 48.6 ; 55.6)	14.0 ( 12.9 ; 15.1)	-73.2 (-75.4 ; -70.8)
**São Tomé e Príncipe**	Todos os fatores de risco	240,9 (206,0 ; 272,8)	272,0 (212,5 ; 326,4)	12,9 ( -8,6 ; 39,1)	224,4 (186,5 ; 263,0)	260,1 (217,3 ; 295,7)	15,9 ( -5,5 ; 41,8)	230.6 (197.7 ; 262.7)	267.1 (219.4 ; 304.8)	15.8 ( -3.4 ; 38.6)
	Poluição do ar	87,9 ( 72,5 ; 107,3)	73,5 ( 54,9 ; 93,9)	-16,4 (-36,4 ; 10,2)	80,8 ( 64,8 ; 100,3)	72,4 ( 55,4 ; 88,2)	-10,4 (-31,9 ; 16,1)	84.0 ( 69.4 ; 101.7)	73.1 ( 56.3 ; 89.5)	-12.9 (-32.1 ; 11.2)
	Consumo de álcool	1,3 ( -2,5 ; 5,7)	3,9 ( -1,4 ; 10,3)	214,7 (-2697,6 ; 2301,0)	6,6 ( 1,5 ; 12,5)	11,9 ( 4,9 ; 20,1)	79,8 (-16,3 ; 525,4)	3.7 ( 0.0 ; 8.0)	7.7 ( 2.7 ; 13.8)	106.1 (-92.6 ; 987.4)
	Riscos dietéticos	82,0 ( 61,8 ; 114,3)	91,7 ( 62,9 ; 132,3)	11,8 (-11,2 ; 36,8)	88,5 ( 66,0 ; 119,8)	98,3 ( 71,9 ; 133,7)	11,0 ( -9,5 ; 37,2)	83.4 ( 63.4 ; 114.3)	94.9 ( 68.3 ; 130.9)	13.8 ( -6.5 ; 37.5)
	Índice de massa corporal elevado	40,8 ( 21,0 ; 65,1)	64,8 ( 40,1 ; 96,1)	59,1 ( 14,3 ; 142,9)	21,5 ( 7,3 ; 42,0)	50,7 ( 29,9 ; 79,0)	135,6 ( 58,2 ; 376,5)	31.6 ( 14.9 ; 52.7)	58.2 ( 36.2 ; 86.4)	84.2 ( 35.0 ; 190.2)
	Glicemia de jejum elevada	42,7 ( 25,3 ; 71,2)	73,4 ( 43,0 ; 115,0)	71,8 ( 21,1 ; 152,3)	47,7 ( 25,7 ; 82,7)	78,2 ( 47,0 ; 119,5)	64,0 ( 18,5 ; 146,4)	43.8 ( 25.8 ; 72.4)	75.6 ( 46.5 ; 116.9)	72.8 ( 31.4 ; 133.4)
	Colesterol LDL elevado	44,1 ( 29,9 ; 61,5)	59,2 ( 37,4 ; 83,0)	34,5 ( 6,5 ; 67,7)	43,7 ( 27,9 ; 63,6)	58,9 ( 39,4 ; 80,6)	34,9 ( 7,2 ; 68,4)	43.3 ( 28.8 ; 61.0)	59.3 ( 39.6 ; 81.4)	36.9 ( 12.1 ; 66.6)
	Pressão arterial sistólica elevada	166,4 (132,1 ; 200,7)	193,5 (142,8 ; 241,7)	16,3 (-11,9 ; 50,3)	143,7 (111,1 ; 179,0)	176,4 (139,9 ; 210,6)	22,8 ( -2,3 ; 56,2)	154.9 (125.9 ; 183.4)	186.1 (144.7 ; 223.2)	20.1 ( -3.6 ; 48.6)
	Disfunção renal	20,0 ( 15,0 ; 25,0)	30,4 ( 22,3 ; 39,7)	52,2 ( 23,1 ; 90,2)	15,5 ( 10,9 ; 20,9)	23,7 ( 17,1 ; 30,6)	53,0 ( 24,6 ; 90,1)	17.8 ( 13.0 ; 22.7)	27.3 ( 20.3 ; 35.1)	53.8 ( 28.1 ; 87.3)
	Baixo nível de atividade física	6,4 ( 2,6 ; 13,3)	9,0 ( 3,7 ; 18,3)	41,3 ( 8,6 ; 82,0)	5,5 ( 1,6 ; 13,1)	7,1 ( 2,1 ; 16,8)	29,1 ( 0,6 ; 67,7)	5.9 ( 2.2 ; 13.2)	8.2 ( 3.0 ; 17.3)	37.7 ( 12.3 ; 70.2)
	Temperatura não ideal	0,9 ( -3,0 ; 4,6)	1,3 ( -0,7 ; 4,8)	40,9 (-303,6 ; 394,2)	0,9 ( -2,7 ; 4,4)	1,2 ( -0,8 ; 4,7)	43,8 (-316,7 ; 362,9)	0.9 ( -2.8 ; 4.4)	1.2 ( -0.7 ; 4.7)	44.4 (-370.9 ; 366.8)
	Outros riscos ambientais	7,7 ( 2,4 ; 13,3)	8,8 ( 3,1 ; 15,2)	14,6 ( -8,7 ; 52,3)	9,7 ( 4,7 ; 15,9)	11,3 ( 5,8 ; 17,6)	16,7 ( -6,3 ; 46,6)	8.4 ( 3.4 ; 14.0)	10.0 ( 4.4 ; 15.9)	18.3 ( -1.9 ; 47.9)
	Tabagismo	6,1 ( 4,7 ; 7,8)	7,0 ( 5,1 ; 9,1)	14,8 (-17,7 ; 62,0)	17,7 ( 13,7 ; 22,3)	23,4 ( 18,1 ; 28,6)	32,3 ( -0,5 ; 79,4)	11.5 ( 9.1 ; 14.0)	14.9 ( 11.6 ; 18.2)	29.4 ( 0.1 ; 72.0)
**Timor-Leste**	Todos os fatores de risco	255,1 (202,6 ; 310,9)	298,2 (246,9 ; 347,9)	16,9 ( -9,0 ; 49,2)	237,9 (182,3 ; 321,3)	346,6 (263,2 ; 447,6)	45,7 ( 9,6 ; 82,9)	247.0 (202.6 ; 304.4)	322.3 (260.4 ; 389.9)	30.5 ( 2.5 ; 60.2)
	Poluição do ar	98,5 ( 73,5 ; 129,6)	89,6 ( 71,5 ; 108,6)	-9,0 (-33,0 ; 22,3)	88,4 ( 63,6 ; 123,7)	102,6 ( 71,9 ; 141,3)	16,1 (-16,8 ; 51,6)	93.6 ( 71.2 ; 121.9)	96.1 ( 73.8 ; 120.9)	2.6 (-23.0 ; 31.3)
	Consumo de álcool	-0,2 ( -0,7 ; 0,3)	0,3 ( -0,9 ; 1,9)	-233,5 (-2319,5 ; 2717,1)	0,9 ( -2,5 ; 5,0)	10,2 ( 0,6 ; 21,6)	1094,3 (-8391,8 ; 11248,0)	0.4 ( -1.4 ; 2.5)	5.2 ( 0.1 ; 11.6)	1361.0 (-5698.7 ; 5258.1)
	Riscos dietéticos	110,6 ( 75,5 ; 154,4)	121,1 ( 85,6 ; 164,7)	9,5 (-16,5 ; 42,3)	115,9 ( 78,9 ; 164,4)	158,6 (107,4 ; 222,2)	36,9 ( 2,2 ; 75,5)	113.3 ( 80.3 ; 155.5)	139.7 ( 98.5 ; 190.0)	23.3 ( -5.1 ; 52.1)
	Índice de massa corporal elevado	13,1 ( 3,0 ; 30,6)	20,5 ( 6,7 ; 42,0)	56,4 ( 4,4 ; 199,8)	8,7 ( 1,4 ; 23,7)	22,8 ( 7,1 ; 47,2)	163,1 ( 61,7 ; 605,6)	10.9 ( 2.3 ; 26.8)	21.7 ( 7.1 ; 44.2)	99.5 ( 36.3 ; 308.3)
	Glicemia de jejum elevada	30,1 ( 18,5 ; 49,0)	89,0 ( 57,0 ; 136,3)	196,0 (101,9 ; 326,6)	34,4 ( 18,9 ; 59,9)	103,4 ( 63,7 ; 163,1)	200,7 ( 99,0 ; 358,7)	32.0 ( 19.6 ; 52.7)	96.0 ( 61.3 ; 148.9)	199.7 (117.9 ; 316.8)
	Colesterol LDL elevado	48,3 ( 32,7 ; 68,4)	61,7 ( 39,9 ; 87,0)	27,9 ( -2,7 ; 65,4)	42,3 ( 27,0 ; 63,8)	65,3 ( 39,4 ; 97,4)	54,4 ( 12,5 ; 98,9)	45.6 ( 30.5 ; 64.0)	63.6 ( 41.2 ; 89.3)	39.6 ( 7.3 ; 75.0)
	Pressão arterial sistólica elevada	168,4 (127,6 ; 214,7)	190,1 (148,9 ; 236,4)	12,9 (-15,1 ; 51,4)	149,2 (109,9 ; 204,3)	230,8 (169,5 ; 308,2)	54,7 ( 14,2 ; 101,2)	159.4 (125.0 ; 204.5)	210.4 (163.4 ; 263.8)	32.0 ( 1.0 ; 67.3)
	Disfunção renal	23,4 ( 16,6 ; 31,2)	35,2 ( 25,8 ; 46,0)	50,7 ( 15,7 ; 95,4)	19,5 ( 12,7 ; 28,7)	35,5 ( 23,4 ; 50,7)	82,2 ( 36,7 ; 134,1)	21.5 ( 15.5 ; 29.4)	35.4 ( 25.2 ; 47.6)	64.5 ( 28.1 ; 103.7)
	Baixo nível de atividade física	5,7 ( 2,0 ; 13,0)	7,6 ( 2,6 ; 16,6)	32,5 ( -3,5 ; 78,3)	6,2 ( 1,8 ; 13,9)	9,7 ( 3,0 ; 22,2)	56,8 ( 16,5 ; 103,8)	6.0 ( 2.1 ; 13.3)	8.6 ( 2.8 ; 19.5)	44.9 ( 13.0 ; 81.2)
	Temperatura não ideal	4,2 ( 0,7 ; 7,9)	4,3 ( 1,4 ; 8,2)	2,5 (-47,5 ; 103,8)	3,9 ( 0,7 ; 7,5)	4,9 ( 1,6 ; 9,7)	26,9 (-34,7 ; 154,0)	4.0 ( 0.7 ; 7.6)	4.6 ( 1.5 ; 9.0)	14.0 (-40.0 ; 126.9)
	Outros riscos ambientais	8,5 ( 2,6 ; 15,7)	10,9 ( 4,3 ; 18,8)	27,9 ( -1,2 ; 83,5)	12,7 ( 6,4 ; 20,9)	19,2 ( 10,6 ; 30,2)	50,8 ( 11,8 ; 98,9)	10.6 ( 4.6 ; 17.9)	15.0 ( 7.7 ; 23.4)	41.9 ( 9.3 ; 83.4)
	Tabagismo	25,3 ( 18,6 ; 33,4)	23,5 ( 17,9 ; 30,3)	-7,0 (-32,4 ; 27,8)	69,6 ( 51,3 ; 95,4)	93,3 ( 66,3 ; 126,1)	33,9 ( -5,2 ; 76,5)	47.6 ( 36.7 ; 62.5)	58.2 ( 42.8 ; 76.5)	22.5 (-10.5 ; 59.1)

LDL: colesterol lipoproteína de baixa densidade.

Particularmente para as estimativas do Brasil, mais de 200 fontes de dados foram incluídas, desde inquéritos nacionais, como a Pesquisa Nacional de Saúde (PNS), a Vigilância de Fatores de Risco e Proteção para Doenças Crônicas por Inquérito telefônico (VIGITEL), a Pesquisa Nacional por Amostra de Domicílios, até a Pesquisa Nacional de Saúde do Escolar e estudos de coorte.^9-16^ Diferentes fontes de dados foram empregadas de acordo com as particularidades de cada PLP.^2,4^

Para estimar a carga de doença atribuível aos FR, o GBD segue a estrutura estabelecida para avaliação comparada de risco (*Comparative Risk Assessment* (CRA)). Sumariamente, a CRA processa-se através de 5 passos: 1) estimar o nível de exposição a partir de fontes disponíveis, como inquéritos domiciliares, dados administrativos, censos, registros vitais e medidas ambientais. Após a identificação dos dados, são feitas padronizações das diferentes definições, além de ajustes por sexo e grupos etários padronizados – etapa chamada de *Crosswalking*. Em seguida, realiza-se análises de suavização espaço-temporal para estimar dados no tempo, grupo etário e área e, por fim, os intervalos de confiança a 95% (IC 95%) das estimativas são calculados; 2) identificar pares de risco-desfecho, conforme evidências disponíveis; 3) calcular o risco relativo (RR), identificado por meio dos estudos de coorte publicados, sintetizados por métodos de meta-análise e meta-regressão. Os RR utilizados pelo GBD são universais, os mesmos para morbidade e mortalidade, e aplicados para homens e mulheres e para todos os países e regiões geográficas; 4) estimar o nível mínimo teórico de exposição ao risco [*Theoretical Minimum Risk Exposure Level* (TMREL)], definido como o nível mínimo de exposição para cada FR que resultaria na menor probabilidade possível de determinado evento clínico ser a ele atribuído. O TMREL é utilizado para o cálculo da fração atribuível populacional (PAF, *population attributable factor*) para diferentes causas de morte, doenças ou incapacidades; 5) calcular a fração atribuível populacional, definida como a proporção do número de casos que pode ser independentemente atribuída a uma determinada exposição.^4,15^

De acordo com o estudo GBD 2019, o TMREL estimado para os FR avaliados no presente estudo são:1) Pressão Arterial Sistólica (PAS): 110 a 115 mm Hg; 2) Glicemia de jejum: 85 a 99 mg/dL; 3) Colesterol LDL: entre 27 e 50 mg/dL; 4) IMC: 20 a 25 kg/m^2^ para adultos; 5) função renal: relação albumina/creatinina <30 mg/g ou taxa de filtração glomerular >60 mL/min por 1·73 m^2^; 6) poluição do ar ambiental: 2,4 a 5,9 μg/m3; 7) tabagismo: nenhuma exposição, incluindo fumo passivo; 8) hábitos dietéticos, incluindo consumo de 1 a 5 g de sal e 200 a 400 g de frutas e vegetais diariamente, entre outros; 9) atividade física: 8000 METs ao dia; 10) uso de álcool: nenhum consumo; 11) temperatura ideal: 25,6ºC. Neste estudo, também foi considerado o 12º grupo de outros fatores de risco ambientais, que não inclui a poluição do ar, a temperatura ambiente e a exposição à fumaça do cigarro.^4^

Para a estimativa da exposição aos fatores de risco, o GBD utiliza a medida síntese de exposição de risco [*Summary Exposure Value* (SEV)], que representa a prevalência ponderada pelo risco. A escala para o SEV varia de 0 a 100%, sendo que 0% reflete nenhuma exposição ao risco e 100% indica exposição máxima. O declínio no SEV indica uma exposição reduzida, e o aumento no SEV, o oposto. A SEV é estimada para cada idade, sexo, localização e ano. A metodologia detalhada para estimação do SEV foi previamente publicada.^4,15,6^

### Definições das doenças cardiovasculares

Definições padronizadas para as DCVs foram usadas no estudo.^2^ Doenças isquêmicas do coração incluem infarto agudo do miocárdio,^17^ angina estável (definida pelo *Rose Angina Questionnaire*), doença isquêmica do coração crônica e insuficiência cardíaca secundária à isquemia. Para acidente vascular cerebral (AVC), foram considerados sinais clínicos agudos e persistentes de disfunção cerebral que duraram mais de 24 horas ou causaram óbito (Organização Mundial da Saúde). Doença arterial periférica dos membros inferiores foi definida como índice tornozelo-braquial <0,9, e para o aneurisma de aorta, considerou-se a presença de aneurismas torácicos e abdominais. Fibrilação e *flutter* atriais foram diagnosticados por eletrocardiograma. Para a doença cardíaca hipertensiva, considerou-se insuficiência cardíaca sintomática devido aos efeitos diretos e indiretos a longo prazo atribuíveis à hipertensão arterial sistêmica. A miocardiopatia foi definida como insuficiência cardíaca sintomática causada por doença primária do miocárdio ou exposição a toxinas, enquanto a miocardite aguda foi definida como uma condição aguda e autolimitada secundária à inflamação. Para endocardite e doença cardíaca reumática, utilizou-se o diagnóstico clínico, sendo que estimativas para doença cardíaca reumática incluíram casos identificados pela história clínica, exame físico ou critérios ecocardiográficos padronizados para doença definitiva (inclusive em caso de doença subclínica). Para as doenças valvares não reumáticas, foram consideradas calcificação da valva aórtica, doença degenerativa da válvula mitral, entre outras.^2,8^

### Análise estatística

Foram empregados os modelos estatísticos do estudo GBD 2019 (Suplemento 1: Métodos Suplementares).^2,4,7^ As fontes de dados para modelos estão disponíveis *online* na página do Global Health Data Exchange (http://ghdx.healthdata.org/).^8^

### Métricas

No presente estudo, as métricas utilizadas para estimativa da carga de doença atribuível aos FR foram mortalidade e anos de vida ajustados por incapacidade – *Disability-Adjusted Life Years* (DALYs) – de 1990 a 2019.

Para o Brasil, as estimativas para mortalidade do GBD têm algumas particularidades. A mortalidade foi estimada utilizando dados do Sistema de Informação sobre Mortalidade (SIM) codificado de acordo com a Classificação Internacional de Doenças.^18^ Para ajustes de qualidade dos registros das causas de morte, foram feitas correções para sub-registro dos óbitos e para causas consideradas pouco úteis para a saúde pública, denominadas *garbage codes*, ou causas inespecíficas. Algoritmos de redistribuição dos *garbage codes* foram desenvolvidos pelo estudo GBD, considerando-se evidências de várias fontes, tais como literatura médica, opinião de especialistas e técnicas estatísticas.^7^

Para cálculo dos DALYs, somam-se os anos de vida perdidos por morte prematura (*Years of Life Lost*, YLLs), tendo como referência a expectativa de vida máxima observada, aos anos vividos com incapacidade (*Years Lived with Disability*, YLD). Os YLD representam a carga de doença não fatal e são determinados pela prevalência da condição multiplicada pelo grau de incapacidade (*disability weight*) causado pela condição. As prevalências das condições foram estimadas por meio de dados representativos de populações, incluindo estudos de coortes, registros, inquéritos populacionais e dados administrativos, utilizando métodos estatísticos que ajustam para diferenças nas definições e métodos dos estudos. *Disability weights* refletem a gravidade de diferentes condições e foram desenvolvidos por meio de entrevistas com o público geral, previamente validadas.^8^

Nas comparações no tempo e entre os PLP, consideraram-se as taxas padronizadas por idade por meio do método direto, utilizando a composição etária global do GBD 2019. Para as outras análises, foram apresentadas as taxas não padronizadas. Para cada um dos FR analisados, foi estimada a carga atribuível para DCVs total e para cada doença separadamente, quando aplicável. Construiu-se o *ranking* dos FR para avaliação das mudanças ocorridas entre 1990 e 2019, segundo sexo, bem como o *ranking* dos FR para cada um dos PLP em 2019. O II 95% foi calculado e descrito cada estimativa, conforme previamente descrito na metodologia do GBD.^2^

### Índice sociodemográfico

O índice sociodemográfico (*Sociodemographic Index*, SDI) é utilizado pelo GBD como estimativa do nível socioeconômico de cada país para avaliação de sua associação com as métricas de fatores de risco e carga de DCV, como uma função da transição epidemiológica global.^4,7^ O SDI foi calculado para cada país ou território de 1990 a 2019 e representa a média geométrica ponderada da renda *per capita*, nível de escolaridade e taxa de fecundidade total, permitindo comparar o desempenho de cada país com o de outros com nível socioeconômico semelhante.

Adicionalmente, foi utilizado o software SPSS versão 23.0 para Mac OSX (*SPSS Inc., Chicago, Illinois*) para realização de correlação (método de *Spearman*) entre a variação percentual das taxas de mortalidade e SEV padronizadas por idade entre 1990 e 2019 e o SDI de cada PLP em 2019. Um valor p<0.05 foi considerado estatisticamente significativo.

## Resultados

As características geográficas e sociodemográficas de cada um dos PLP podem ser vistas na Tabela S1.

A contribuição percentual das DCVs atribuíveis aos FR para a mortalidade em 2019 nos diferentes PLP foi heterogênea, variando de 32,1%, 31,7%, 30,7% e 28,2% em Portugal, Timor Leste, Cabo Verde e Brasil, respectivamente, até índices baixos como 12% a 13,9% nos demais países (Figura S1). O percentual atribuível aos FR é elevado (>75%) em todos os PLP, sendo mais baixo em Portugal (78.8%) e Brasil (82.6%). A Tabela S2 apresenta as taxas de SEV padronizadas por idade para cada FR cardiovascular, com II95%, segundo sexo, para 1990 e 2019, e o percentual de mudança no período. Foi observado aumento expressivo dos SEV relacionados a consumo de álcool e IMC elevado em todos os países. Para PAS elevada, foi observada redução significativa em Portugal e uma tendência à estabilidade no Brasil e Timor Leste, contrastando com tendência à elevação nos demais países, especialmente Guiné Equatorial (Tabela S2).

**Figura 1:**
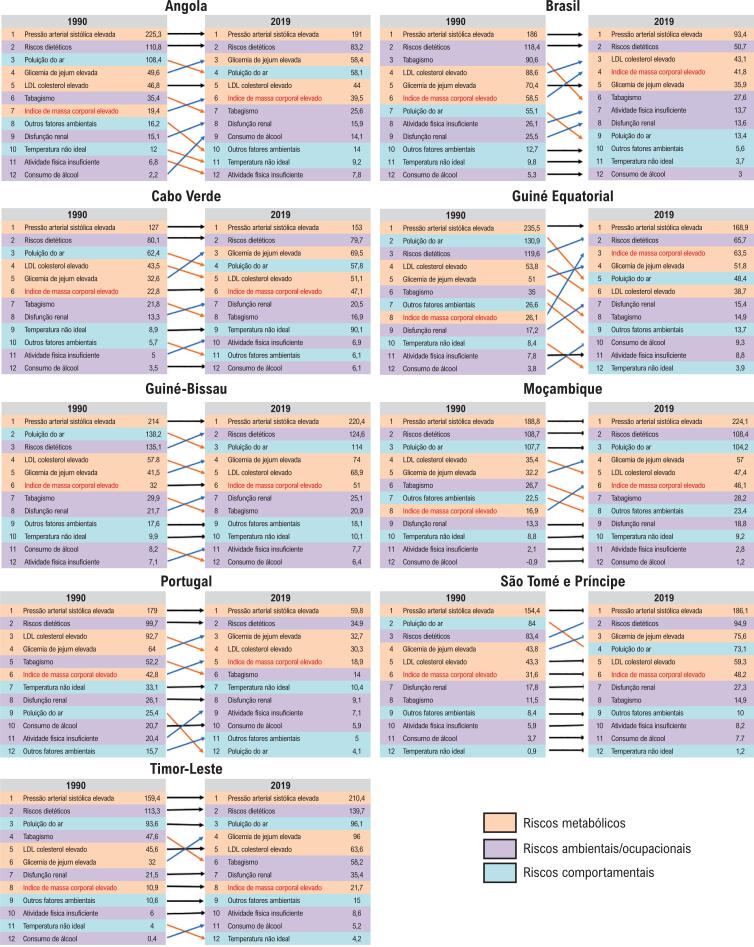
Ranking das taxas de mortalidade (/100 000 habitantes) por doenças cardiovasculares padronizadas por idade atribuíveis aos fatores de risco nos países de língua portuguesa em 1990 e 2019.

A Figura 1 mostra o *ranking* das taxas de mortalidade por DCV padronizadas por idade atribuíveis aos FR nos PLP, segundo sexo, em 1990 e 2019. Observa-se que a PAS elevada manteve-se em todos os países como principal fator de risco para DCV no período. Houve um aumento da importância da glicemia de jejum elevada – exceto no Brasil – e este fator ocupou entre a 3ᵃ e a 4ᵃ posição em todos os PLP em 2019. Por outro lado, o tabagismo caiu no ranking de mortalidade atribuível em todos os países, exceto São Tomé e Príncipe (onde se manteve na 8ᵃ posição). Houve uma redução mais expressiva no Brasil (3° para 6°) e Guiné Equatorial (6ᵃ para 8ᵃ). Colesterol LDL elevado apresentou padrão estável ou de redução em todos os países, exceto em Portugal e Brasil. A Figura S2 mostra padrão semelhante para as taxas de DALYs atribuíveis aos FR cardiovasculares.

A Figura S3 mostra as taxas de mortalidade e DALYs por DCVs brutos e padronizados por idade atribuíveis aos FR selecionados entre 1990 e 2019. Verifica-se aumento nos números absolutos de óbitos e DALYs por DCVs atribuíveis a todos os FR, exceto para algumas tendências em Portugal, com um declínio para fatores dietéticos, colesterol LDL elevado e PAS elevada, e estabilidade para IMC elevado e glicemia de jejum elevada. Por outro lado, ao analisar as taxas de mortalidade e DALYs padronizadas por idade, observa-se um contraste entre Brasil e Portugal – que apresentaram declínio para todos os FR – e os demais PLP, que apresentaram uma tendência de estabilidade ou aumento. A exceção foi o tabagismo, que apresentou declínio em todos os PLP, exceto em Moçambique, São Tomé e Príncipe e Timor Leste (Figura S3, Tabelas 1 e 2, Tabelas Suplementares 3 e 4).

**Tabela 2:** Anos de vida perdidos por incapacidade (DALYs, Disability-Adjusted Life Years) padronizadas por idade, por 100 000 habitantes

Países	Fatores de risco	Mulheres			Homens			Both		
		1990	2019	Porcentagem de variação %	1990	2019	Percent Change %	1990	2019	Percent Change %
**Angola**	Todos os fatores de risco	5999,5 (4395,2 ; 7665,4)	4685,7 (3830,6 ; 5842,6)	-21,9 (-42,3 ; 9,6)	7806,8 (6071,9 ; 9648,6)	5927,2 (4931,1 ; 7175,3)	-24,1 (-42,7 ; 4,1)	6929.8 (5616.7 ; 8339.7)	5274.4 (4415.4 ; 6432.1)	-23.9 (-40.1 ; -0.7)
	Poluição do ar	2127,2 (1414,2 ; 2901,9)	1115,8 (806,5 ; 1503,7)	-47,5 (-63,4 ; -22,7)	3009,4 (2271,4 ; 3794,0)	1590,1 (1196,9 ; 2069,5)	-47,2 (-62,6 ; -24,9)	2577.7 (1987.6 ; 3287.7)	1336.5 (992.5 ; 1759.4)	-48.2 (-62.4 ; -28.8)
	Consumo de álcool	15,3 (-77,1 ; 114,3)	198,8 ( 70,0 ; 347,4)	1202,9 (-11035,7 ; 5853,2)	134,6 (-44,1 ; 339,4)	503,4 (303,1 ; 728,6)	273,9 (-2894,5 ; 3150,6)	74.5 (-35.3 ; 193.4)	335.1 (209.2 ; 493.8)	349.7 (-3664.4 ; 3172.1)
	Riscos dietéticos	2014,8 (1306,8 ; 2966,1)	1425,8 (965,2 ; 2141,5)	-29,2 (-49,7 ; 2,1)	3077,2 (2228,5 ; 4197,0)	2070,4 (1501,1 ; 2877,1)	-32,7 (-50,8 ; -4,7)	2556.4 (1894.9 ; 3468.2)	1726.4 (1214.6 ; 2489.6)	-32.5 (-49.2 ; -8.7)
	Índice de massa corporal elevado	509,4 (110,9 ; 1175,3)	1039,4 (534,9 ; 1624,8)	104,0 ( 12,2 ; 528,9)	551,6 (108,5 ; 1342,1)	1064,7 (505,3 ; 1729,3)	93,0 ( 8,1 ; 500,2)	532.7 (113.3 ; 1263.7)	1055.2 (545.3 ; 1649.2)	98.1 ( 13.9 ; 486.0)
	Glicemia de jejum elevada	616,7 (366,8 ; 994,2)	732,7 (425,4 ; 1157,0)	18,8 (-23,8 ; 92,8)	1328,9 (862,0 ; 2000,9)	1562,0 (1032,1 ; 2231,3)	17,5 (-21,1 ; 83,9)	963.5 (652.6 ; 1428.2)	1101.7 (739.8 ; 1595.8)	14.3 (-18.0 ; 66.0)
	Colesterol LDL elevado	863,5 (551,8 ; 1271,1)	791,9 (523,5 ; 1110,4)	-8,3 (-37,2 ; 33,9)	1329,1 (921,5 ; 1815,8)	1108,4 (789,7 ; 1522,1)	-16,6 (-41,3 ; 20,7)	1105.2 (792.6 ; 1493.0)	943.7 (663.9 ; 1292.7)	-14.6 (-37.7 ; 17.8)
	Pressão arterial sistólica elevada	4547,3 (3319,4 ; 5912,5)	3629,9 (2857,3 ; 4543,0)	-20,2 (-41,9 ; 14,2)	5449,8 (4097,4 ; 6949,2)	4190,3 (3338,0 ; 5147,4)	-23,1 (-43,4 ; 8,2)	5025.9 (3962.3 ; 6245.9)	3912.6 (3189.4 ; 4828.3)	-22.2 (-40.0 ; 2.6)
	Disfunção renal	290,8 (188,4 ; 422,6)	284,8 (203,9 ; 389,4)	-2,1 (-28,8 ; 39,4)	399,0 (275,5 ; 537,0)	364,0 (270,1 ; 491,8)	-8,8 (-32,1 ; 27,1)	346.4 (248.9 ; 471.7)	322.6 (236.2 ; 437.1)	-6.9 (-28.4 ; 22.6)
	Baixo nível de atividade física	131,1 ( 49,5 ; 276,6)	136,6 ( 55,2 ; 283,5)	4,2 (-26,1 ; 48,7)	99,7 ( 26,1 ; 252,5)	100,8 ( 29,7 ; 250,9)	1,1 (-26,5 ; 47,9)	117.8 ( 40.1 ; 266.5)	123.0 ( 46.0 ; 267.5)	4.4 (-23.3 ; 41.0)
	Temperatura não ideal	215,6 (108,2 ; 345,1)	153,6 ( 90,7 ; 237,8)	-28,7 (-50,8 ; 17,6)	281,9 (149,3 ; 452,1)	192,5 (112,2 ; 302,1)	-31,7 (-51,8 ; 2,2)	249.8 (134.5 ; 392.5)	172.2 (102.1 ; 268.5)	-31.1 (-49.4 ; 2.4)
	Outros riscos ambientais	267,9 (100,7 ; 489,2)	209,8 ( 90,4 ; 363,2)	-21,7 (-42,7 ; 17,1)	485,8 (257,2 ; 760,0)	354,1 (196,6 ; 537,9)	-27,1 (-46,1 ; 1,6)	377.5 (184.9 ; 618.3)	275.0 (141.5 ; 437.0)	-27.1 (-43.4 ; -3.3)
	Tabagismo	346,2 (233,2 ; 481,0)	272,9 (195,7 ; 369,1)	-21,2 (-47,4 ; 20,3)	1602,4 (1208,8 ; 2044,9)	1163,6 (921,0 ; 1489,6)	-27,4 (-47,9 ; 4,4)	977.9 (757.7 ; 1229.1)	678.9 (539.0 ; 876.8)	-30.6 (-49.5 ; -2.7)
**Brasil**	Todos os fatores de risco	5140,9 (4871,7 ; 5380,8)	2380,3 (2200,5 ; 2544,9)	-53,7 (-55,9 ; -51,4)	7756,5 (7453,1 ; 8034,5)	3888,3 (3646,9 ; 4112,3)	-49,9 (-52,8 ; -47,3)	6385.9 (6112.9 ; 6619.3)	3075.2 (2881.5 ; 3230.7)	-51.8 (-53.9 ; -50.0)
	Poluição do ar	1074,6 (805,7 ; 1372,7)	261,8 (189,5 ; 335,6)	-75,6 (-82,6 ; -66,5)	1617,5 (1168,8 ; 2094,1)	421,0 (304,6 ; 550,7)	-74,0 (-81,6 ; -63,1)	1333.9 (972.1 ; 1717.8)	335.8 (245.2 ; 436.8)	-74.8 (-82.3 ; -65.2)
	Consumo de álcool	24,8 (-16,1 ; 70,3)	14,8 ( -6,7 ; 38,8)	-40,2 (-202,7 ; 184,0)	369,0 (227,5 ; 534,8)	183,5 (103,5 ; 267,1)	-50,3 (-62,5 ; -35,6)	189.0 (114.0 ; 272.5)	92.6 ( 53.3 ; 134.8)	-51.0 (-64.3 ; -34.6)
	Riscos dietéticos	1976,8 (1603,5 ; 2462,7)	830,8 (654,2 ; 1067,4)	-58,0 (-62,0 ; -54,2)	3373,7 (2700,5 ; 4120,9)	1546,0 (1214,4 ; 1948,9)	-54,2 (-58,3 ; -50,0)	2641.3 (2140.9 ; 3252.7)	1160.8 (913.6 ; 1466.2)	-56.1 (-59.5 ; -52.8)
	Índice de massa corporal elevado	1462,1 (907,2 ; 2094,3)	924,3 (682,4 ; 1181,6)	-36,8 (-45,0 ; -22,2)	1768,1 (955,9 ; 2704,4)	1315,5 (882,4 ; 1774,0)	-25,6 (-37,9 ; 2,2)	1611.6 (936.5 ; 2376.0)	1108.9 (778.3 ; 1460.7)	-31.2 (-40.5 ; -12.4)
	Glicemia de jejum elevada	1007,6 (715,9 ; 1432,7)	483,6 (345,7 ; 673,9)	-52,0 (-58,5 ; -44,5)	1593,2 (1141,6 ; 2253,9)	906,1 (635,8 ; 1274,9)	-43,1 (-49,4 ; -35,1)	1279.7 (922.2 ; 1800.5)	673.5 (485.1 ; 947.7)	-47.4 (-52.2 ; -41.9)
	Colesterol LDL elevado	1425,8 (1165,2 ; 1745,1)	692,2 (567,0 ; 842,5)	-51,5 (-54,8 ; -47,9)	2496,0 (2090,2 ; 2980,2)	1310,6 (1097,4 ; 1543,0)	-47,5 (-50,9 ; -43,8)	1940.1 (1614.4 ; 2322.9)	981.3 (817.1 ; 1162.4)	-49.4 (-52.0 ; -46.8)
	Pressão arterial sistólica elevada	3264,0 (2911,5 ; 3596,7)	1551,6 (1365,7 ; 1731,8)	-52,5 (-55,6 ; -49,0)	4825,1 (4330,2 ; 5295,9)	2560,7 (2294,0 ; 2810,7)	-46,9 (-50,3 ; -43,5)	4011.3 (3600.8 ; 4407.4)	2019.3 (1806.5 ; 2216.0)	-49.7 (-52.0 ; -47.1)
	Disfunção renal	405,4 (330,0 ; 485,6)	204,4 (166,3 ; 247,4)	-49,6 (-53,4 ; -46,4)	625,9 (507,2 ; 754,1)	351,7 (284,7 ; 423,0)	-43,8 (-47,6 ; -39,9)	510.8 (414.8 ; 611.6)	272.1 (221.5 ; 325.5)	-46.7 (-49.5 ; -43.9)
	Baixo nível de atividade física	387,4 (187,6 ; 624,2)	197,2 (110,7 ; 303,3)	-49,1 (-54,9 ; -38,3)	483,4 (191,0 ; 878,6)	275,7 (127,9 ; 478,4)	-43,0 (-49,8 ; -27,6)	434.5 (193.2 ; 747.3)	233.1 (118.2 ; 375.7)	-46.4 (-52.1 ; -34.5)
	Temperatura não ideal	160,5 ( 44,4 ; 251,2)	55,1 ( 15,0 ; 84,8)	-65,7 (-81,5 ; -34,3)	233,7 ( 53,3 ; 364,4)	87,6 ( 15,8 ; 135,2)	-62,5 (-86,1 ; -17,9)	195.7 ( 47.7 ; 304.9)	70.1 ( 13.4 ; 108.1)	-64.2 (-84.6 ; -30.1)
	Outros riscos ambientais	180,0 ( 62,9 ; 301,9)	68,7 ( 23,0 ; 123,4)	-61,9 (-66,9 ; -58,2)	387,6 (201,3 ; 572,2)	150,1 ( 70,8 ; 237,3)	-61,3 (-67,0 ; -57,0)	277.4 (127.9 ; 428.4)	105.1 ( 44.6 ; 175.8)	-62.1 (-67.2 ; -58.3)
	Tabagismo	1720,6 (1562,0 ; 1894,4)	524,3 (473,0 ; 579,1)	-69,5 (-72,8 ; -66,1)	3008,3 (2835,8 ; 3176,8)	972,3 (899,1 ; 1048,2)	-67,7 (-70,1 ; -65,1)	2332.2 (2200.7 ; 2463.5)	731.3 (681.2 ; 782.2)	-68.6 (-70.9 ; -66.4)
**Cabo Verde**	Todos os fatores de risco	3232,1 (2881,9 ; 3588,7)	3229,7 (2736,6 ; 3742,1)	-0,1 (-16,2 ; 19,6)	4980,5 (4462,4 ; 5466,7)	5375,2 (4739,6 ; 6059,0)	7,9 ( -6,4 ; 24,7)	3949.1 (3575.7 ; 4282.9)	4178.4 (3663.4 ; 4722.8)	5.8 ( -8.7 ; 22.2)
	Poluição do ar	1085,5 (916,9 ; 1284,9)	873,8 (686,1 ; 1074,6)	-19,5 (-37,3 ; 3,1)	1872,6 (1601,9 ; 2162,1)	1641,8 (1300,4 ; 2001,5)	-12,3 (-30,3 ; 7,9)	1409.1 (1221.4 ; 1628.4)	1218.3 (971.7 ; 1472.0)	-13.5 (-31.5 ; 6.8)
	Consumo de álcool	56,4 ( 1,2 ; 123,5)	53,8 ( -5,9 ; 122,8)	-4,5 (-146,8 ; 342,6)	209,7 ( 95,7 ; 329,3)	296,6 (160,6 ; 461,8)	41,5 (-14,5 ; 145,4)	120.1 ( 54.2 ; 195.2)	161.2 ( 78.9 ; 260.9)	34.2 (-30.0 ; 158.2)
	Riscos dietéticos	1200,0 (929,4 ; 1610,7)	1100,3 (800,4 ; 1528,9)	-8,3 (-25,5 ; 10,9)	2226,2 (1763,9 ; 2828,4)	1984,2 (1477,5 ; 2687,2)	-10,9 (-26,2 ; 6,6)	1620.7 (1283.0 ; 2085.9)	1494.1 (1100.1 ; 2041.3)	-7.8 (-23.1 ; 8.9)
	Índice de massa corporal elevado	667,6 (350,3 ; 1006,8)	996,0 (686,6 ; 1370,1)	49,2 ( 10,9 ; 132,8)	634,4 (231,2 ; 1133,9)	1415,4 (895,0 ; 2019,2)	123,1 ( 55,0 ; 364,7)	651.1 (305.9 ; 1050.2)	1197.0 (801.8 ; 1682.0)	83.8 ( 35.4 ; 201.0)
	Glicemia de jejum elevada	424,0 (272,3 ; 638,9)	891,1 (569,5 ; 1292,8)	110,2 ( 53,8 ; 192,3)	709,3 (474,4 ; 1049,6)	1390,1 (950,8 ; 1954,4)	96,0 ( 47,5 ; 165,4)	540.6 (356.6 ; 799.3)	1100.1 (744.7 ; 1552.5)	103.5 ( 58.5 ; 165.0)
	Colesterol LDL elevado	641,5 (478,6 ; 832,2)	754,7 (542,3 ; 998,2)	17,6 ( -4,5 ; 41,4)	1211,2 (921,3 ; 1525,0)	1227,4 (907,3 ; 1582,9)	1,3 (-15,5 ; 20,9)	872.9 (669.5 ; 1097.6)	971.8 (717.6 ; 1245.1)	11.3 ( -4.9 ; 31.5)
	Pressão arterial sistólica elevada	2304,0 (1921,5 ; 2702,3)	2322,6 (1862,6 ; 2791,3)	0,8 (-20,1 ; 25,0)	3430,1 (2927,3 ; 3948,6)	3862,3 (3257,9 ; 4532,1)	12,6 ( -5,7 ; 34,3)	2764.5 (2413.2 ; 3140.2)	3014.4 (2510.3 ; 3542.8)	9.0 ( -7.2 ; 28.5)
	Disfunção renal	204,5 (150,6 ; 268,1)	286,8 (208,8 ; 371,1)	40,2 ( 15,4 ; 72,9)	322,2 (228,8 ; 420,9)	445,8 (330,5 ; 564,7)	38,4 ( 16,5 ; 63,7)	252.7 (185.4 ; 328.1)	356.8 (267.5 ; 455.0)	41.2 ( 21.0 ; 67.0)
	Baixo nível de atividade física	66,9 ( 25,1 ; 147,8)	90,3 ( 35,4 ; 195,0)	35,0 ( 9,3 ; 70,9)	85,2 ( 23,4 ; 234,3)	107,9 ( 30,4 ; 270,0)	26,6 ( 1,0 ; 64,6)	74.3 ( 23.7 ; 184.2)	98.6 ( 34.5 ; 227.6)	32.6 ( 10.5 ; 63.0)
	Temperatura não ideal	130,6 ( -1,5 ; 271,7)	119,7 ( 31,3 ; 249,1)	-8,4 (-44,8 ; 44,2)	209,7 ( 14,1 ; 434,8)	197,0 ( 43,0 ; 415,5)	-6,1 (-40,8 ; 38,0)	163.1 ( 11.3 ; 338.4)	153.8 ( 37.2 ; 320.2)	-5.7 (-37.9 ; 45.2)
	Outros riscos ambientais	89,7 ( 16,5 ; 174,7)	75,0 ( 15,3 ; 145,3)	-16,4 (-33,1 ; 9,6)	162,8 ( 49,3 ; 289,0)	142,2 ( 40,7 ; 261,4)	-12,6 (-30,3 ; 3,3)	119.8 ( 29.9 ; 216.5)	103.6 ( 26.8 ; 192.6)	-13.5 (-29.3 ; 0.0)
	Tabagismo	255,0 (200,8 ; 309,6)	177,5 (140,3 ; 219,7)	-30,4 (-47,4 ; -8,8)	1025,1 (887,5 ; 1168,5)	730,9 (610,8 ; 861,7)	-28,7 (-41,4 ; -13,2)	573.3 (506.5 ; 645.6)	427.3 (360.6 ; 500.8)	-25.5 (-38.1 ; -8.9)
**Guné Equatorial**	Todos os fatores de risco	6569,8 (4287,1 ; 9089,7)	4163,8 (2926,5 ; 5799,9)	-36,6 (-60,5 ; 4,9)	9527,0 (7147,6 ; 11927,0)	4260,3 (3067,7 ; 5613,6)	-55,3 (-68,4 ; -33,0)	7918.4 (5983.2 ; 9983.4)	4227.0 (3165.8 ; 5693.3)	-46.6 (-62.5 ; -23.9)
	Poluição do ar	2482,8 (1481,8 ; 3765,3)	982,2 (596,0 ; 1473,7)	-60,4 (-79,0 ; -27,6)	3909,8 (2833,2 ; 5128,4)	1101,5 (689,6 ; 1597,4)	-71,8 (-83,0 ; -54,0)	3128.9 (2266.2 ; 4171.6)	1036.8 (645.1 ; 1528.6)	-66.9 (-80.4 ; -48.9)
	Consumo de álcool	40,9 (-78,3 ; 193,9)	141,0 ( 13,8 ; 305,7)	244,4 (-3080,2 ; 2575,4)	216,4 (-57,0 ; 539,2)	307,6 (129,9 ; 516,4)	42,1 (-1112,9 ; 1274,7)	118.2 (-46.4 ; 314.2)	210.5 ( 79.5 ; 381.6)	78.1 (-1552.1 ; 1339.9)
	Riscos dietéticos	2125,4 (1242,0 ; 3354,3)	1184,7 (689,2 ; 1888,4)	-44,3 (-67,0 ; -7,2)	3671,8 (2554,0 ; 5092,8)	1360,5 (874,5 ; 2048,1)	-62,9 (-74,7 ; -43,6)	2823.4 (1987.6 ; 3980.8)	1266.3 (821.3 ; 1949.9)	-55.2 (-69.8 ; -34.4)
	Índice de massa corporal elevado	732,6 (198,2 ; 1561,9)	1507,2 (908,0 ; 2310,4)	105,7 ( -0,1 ; 524,7)	705,4 (137,8 ; 1707,1)	1398,5 (815,0 ; 2158,8)	98,3 ( -8,0 ; 714,5)	723.5 (187.0 ; 1594.7)	1471.1 (904.3 ; 2214.8)	103.3 ( -1.7 ; 549.0)
	Glicemia de jejum elevada	658,2 (357,3 ; 1088,6)	744,7 (434,3 ; 1193,3)	13,1 (-36,4 ; 99,5)	1553,6 (973,5 ; 2331,5)	1175,5 (754,0 ; 1744,0)	-24,3 (-50,4 ; 20,2)	1046.1 (698.0 ; 1551.0)	921.0 (596.3 ; 1372.7)	-12.0 (-42.2 ; 31.2)
	Colesterol LDL elevado	972,1 (552,2 ; 1559,5)	722,0 (428,7 ; 1124,7)	-25,7 (-57,0 ; 26,1)	1683,6 (1153,8 ; 2312,9)	782,6 (492,7 ; 1176,6)	-53,5 (-68,8 ; -26,2)	1298.4 (875.7 ; 1817.4)	754.8 (478.0 ; 1133.3)	-41.9 (-61.9 ; -13.4)
	Pressão arterial sistólica elevada	4934,3 (3200,0 ; 6958,9)	3270,3 (2258,5 ; 4577,1)	-33,7 (-59,8 ; 11,2)	6611,8 (4760,9 ; 8476,4)	3093,9 (2140,7 ; 4141,8)	-53,2 (-67,7 ; -29,1)	5714.8 (4252.3 ; 7288.3)	3218.6 (2341.0 ; 4384.1)	-43.7 (-61.2 ; -18.4)
	Disfunção renal	321,0 (187,7 ; 508,2)	286,4 (183,8 ; 427,6)	-10,8 (-46,3 ; 51,5)	493,2 (334,7 ; 677,9)	281,3 (190,3 ; 403,4)	-43,0 (-59,7 ; -15,8)	399.3 (278.4 ; 561.6)	286.1 (190.9 ; 411.9)	-28.4 (-51.0 ; 2.9)
	Baixo nível de atividade física	143,2 ( 49,4 ; 319,9)	152,2 ( 62,1 ; 308,1)	6,2 (-36,3 ; 82,9)	122,1 ( 32,9 ; 318,6)	97,6 ( 28,6 ; 233,5)	-20,1 (-50,0 ; 33,6)	136.9 ( 46.4 ; 310.4)	131.4 ( 50.6 ; 274.1)	-4.0 (-37.4 ; 50.0)
	Temperatura não ideal	145,8 (-15,2 ; 347,3)	65,4 ( 9,8 ; 163,8)	-55,1 (-82,2 ; -10,3)	215,1 ( -4,8 ; 506,9)	65,3 ( 8,9 ; 169,8)	-69,7 (-95,6 ; -42,1)	177.5 (-11.7 ; 418.0)	65.9 ( 9.9 ; 164.6)	-62.9 (-85.6 ; -30.0)
	Outros riscos ambientais	435,3 (213,2 ; 748,2)	212,3 (102,9 ; 355,0)	-51,2 (-70,5 ; -21,8)	879,4 (538,8 ; 1293,4)	280,7 (154,4 ; 437,1)	-68,1 (-78,2 ; -53,3)	633.6 (374.6 ; 952.0)	240.9 (126.7 ; 382.1)	-62.0 (-73.4 ; -46.5)
	Tabagismo	263,5 (160,7 ; 408,5)	138,5 ( 84,6 ; 219,1)	-47,4 (-70,5 ; -5,7)	1885,9 (1341,1 ; 2486,9)	702,2 (473,2 ; 1003,9)	-62,8 (-75,6 ; -41,8)	990.3 (726.1 ; 1299.4)	377.1 (258.1 ; 542.7)	-61.9 (-74.9 ; -42.9)
**Guiné-Bissau**	Todos os fatores de risco	6230,3 (4699,3 ; 7828,9)	6284,9 (4934,5 ; 7962,6)	0,9 (-26,3 ; 39,6)	8677,3 (6803,9 ; 10844,6)	7626,5 (6117,8 ; 9356,4)	-12,1 (-34,9 ; 19,9)	7415.5 (5951.3 ; 9059.8)	6919.5 (5515.9 ; 8586.3)	-6.7 (-29.8 ; 23.5)
	Poluição do ar	2680,9 (1920,2 ; 3605,5)	2375,9 (1785,9 ; 3066,2)	-11,4 (-36,9 ; 24,9)	3918,3 (2878,4 ; 5226,8)	3047,4 (2349,2 ; 3813,7)	-22,2 (-43,8 ; 9,1)	3279.7 (2470.8 ; 4272.8)	2692.3 (2078.8 ; 3414.8)	-17.9 (-39.5 ; 11.6)
	Consumo de álcool	90,2 (-11,3 ; 210,3)	76,1 (-23,7 ; 209,3)	-15,6 (-242,3 ; 303,3)	431,4 (195,9 ; 707,7)	350,1 (140,1 ; 607,3)	-18,8 (-62,9 ; 60,8)	254.1 (109.4 ; 424.2)	202.5 ( 68.1 ; 373.0)	-20.3 (-69.4 ; 68.7)
	Riscos dietéticos	2447,2 (1684,4 ; 3503,7)	2406,1 (1665,2 ; 3521,1)	-1,7 (-30,0 ; 38,5)	3923,7 (2770,2 ; 5444,2)	3266,2 (2369,6 ; 4473,7)	-16,8 (-40,0 ; 15,6)	3155.9 (2275.0 ; 4403.3)	2804.6 (1995.1 ; 3895.0)	-11.1 (-34.2 ; 20.6)
	Índice de massa corporal elevado	1065,0 (416,6 ; 1923,0)	1665,5 (942,3 ; 2572,9)	56,4 ( 2,1 ; 186,9)	824,4 (233,4 ; 1772,1)	1257,4 (510,7 ; 2209,9)	52,5 ( -1,7 ; 212,7)	950.3 (330.9 ; 1810.6)	1480.4 (763.7 ; 2401.0)	55.8 ( 6.2 ; 186.6)
	Glicemia de jejum elevada	609,5 (387,3 ; 951,1)	1235,5 (745,1 ; 1946,8)	102,7 ( 31,1 ; 221,6)	969,6 (628,3 ; 1475,4)	1460,9 (933,3 ; 2188,9)	50,7 ( 0,5 ; 132,0)	782.3 (518.7 ; 1164.3)	1332.4 (863.9 ; 2007.8)	70.3 ( 18.8 ; 144.0)
	Colesterol LDL elevado	1079,7 (717,8 ; 1574,2)	1276,2 (872,9 ; 1764,5)	18,2 (-20,2 ; 72,0)	1665,4 (1122,7 ; 2384,7)	1657,0 (1154,2 ; 2188,0)	-0,5 (-30,4 ; 41,9)	1358.3 (940.5 ; 1914.3)	1456.2 (1018.8 ; 1943.0)	7.2 (-23.2 ; 51.4)
	Pressão arterial sistólica elevada	4332,4 (3146,7 ; 5614,9)	4529,6 (3409,1 ; 5890,7)	4,6 (-25,3 ; 47,8)	5534,3 (4184,5 ; 7156,4)	5216,6 (3984,4 ; 6616,8)	-5,7 (-32,5 ; 31,7)	4916.5 (3848.8 ; 6174.5)	4866.1 (3790.0 ; 6146.4)	-1.0 (-26.6 ; 32.7)
	Disfunção renal	414,3 (293,9 ; 562,3)	490,9 (352,1 ; 667,9)	18,5 (-15,8 ; 65,4)	549,5 (381,0 ; 759,5)	558,5 (400,8 ; 742,1)	1,6 (-25,7 ; 37,9)	479.7 (349.3 ; 634.7)	523.4 (379.7 ; 701.7)	9.1 (-18.4 ; 46.1)
	Baixo nível de atividade física	102,1 ( 35,2 ; 236,2)	118,9 ( 42,5 ; 276,8)	16,5 (-17,9 ; 66,6)	145,9 ( 39,6 ; 377,6)	137,4 ( 40,0 ; 350,7)	-5,8 (-31,5 ; 32,2)	123.4 ( 38.7 ; 302.8)	128.1 ( 42.0 ; 307.6)	3.9 (-20.9 ; 40.1)
	Temperatura não ideal	174,0 (-486,0 ; 390,6)	185,3 ( 28,6 ; 332,2)	6,5 (-163,7 ; 90,3)	253,7 (-731,3 ; 532,3)	227,8 ( 12,2 ; 389,2)	-10,2 (-157,2 ; 40,9)	212.6 (-593.7 ; 433.0)	205.4 ( 35.6 ; 361.4)	-3.4 (-168.6 ; 52.4)
	Outros riscos ambientais	286,6 (115,1 ; 489,2)	292,8 (126,2 ; 493,2)	2,2 (-26,4 ; 49,3)	559,3 (303,4 ; 866,5)	484,1 (277,2 ; 732,4)	-13,4 (-37,2 ; 21,5)	417.5 (208.7 ; 652.4)	379.9 (197.8 ; 600.9)	-9.0 (-33.8 ; 22.7)
	Tabagismo	328,4 (227,6 ; 452,0)	287,5 (204,4 ; 385,8)	-12,4 (-42,4 ; 28,9)	1388,6 (1014,8 ; 1840,9)	918,1 (700,6 ; 1162,9)	-33,9 (-54,5 ; -4,6)	837.9 (637.7 ; 1089.7)	580.5 (446.5 ; 734.0)	-30.7 (-51.5 ; -3.9)
**Moçambique**	Todos os fatores de risco	4984,7 (4089,4 ; 5939,3)	4771,3 (3678,2 ; 6235,3)	-4,3 (-29,1 ; 28,7)	6407,0 (5226,9 ; 7754,2)	8455,5 (6898,2 ; 10077,4)	32,0 ( 3,3 ; 67,2)	5688.1 (4798.3 ; 6665.6)	6479.1 (5166.2 ; 8024.3)	13.9 (-11.7 ; 44.9)
	Poluição do ar	2035,6 (1527,2 ; 2728,0)	1691,8 (1227,1 ; 2338,9)	-16,9 (-40,8 ; 16,5)	2776,1 (2089,8 ; 3714,1)	3312,6 (2608,0 ; 4064,1)	19,3 (-11,4 ; 59,6)	2397.5 (1852.1 ; 3150.0)	2440.8 (1876.1 ; 3110.1)	1.8 (-24.4 ; 36.3)
	Consumo de álcool	-20,9 (-52,7 ; 14,1)	-10,9 (-69,6 ; 54,9)	-47,6 (-935,3 ; 689,0)	-7,5 (-117,8 ; 110,2)	118,4 (-120,0 ; 385,4)	-1688,0 (-4458,0 ; 4236,8)	-14.2 (-73.8 ; 48.0)	49.0 (-69.1 ; 186.7)	-444.1 (-2464.3 ; 3711.1)
	Riscos dietéticos	1965,6 (1178,6 ; 3032,0)	1714,4 (977,2 ; 2743,3)	-12,8 (-37,5 ; 20,2)	2806,2 (1846,8 ; 4085,1)	3164,3 (2127,9 ; 4520,4)	12,8 (-16,0 ; 51,4)	2373.1 (1546.7 ; 3505.3)	2383.0 (1518.4 ; 3527.0)	0.4 (-23.6 ; 32.4)
	Índice de massa corporal elevado	470,9 (131,6 ; 997,6)	1098,0 (579,1 ; 1761,4)	133,2 ( 32,4 ; 484,0)	437,0 ( 89,7 ; 1040,9)	1477,1 (679,9 ; 2453,0)	238,0 ( 98,9 ; 908,9)	457.0 (115.5 ; 1038.7)	1283.8 (634.4 ; 2077.3)	180.9 ( 67.8 ; 605.3)
	Glicemia de jejum elevada	415,2 (266,7 ; 674,7)	611,2 (351,5 ; 1029,6)	47,2 (-14,4 ; 136,1)	755,9 (475,7 ; 1128,2)	1745,4 (1139,4 ; 2522,4)	130,9 ( 61,8 ; 241,8)	571.2 (381.3 ; 824.4)	1101.3 (720.8 ; 1622.2)	92.8 ( 37.9 ; 173.5)
	Colesterol LDL elevado	616,5 (432,7 ; 878,1)	694,6 (447,4 ; 1017,6)	12,7 (-20,9 ; 56,5)	949,1 (676,3 ; 1329,1)	1496,6 (1065,4 ; 2013,3)	57,7 ( 17,1 ; 116,3)	777.2 (566.7 ; 1090.9)	1062.9 (745.3 ; 1448.8)	36.7 ( 2.9 ; 82.7)
	Pressão arterial sistólica elevada	3718,3 (2937,6 ; 4645,2)	3668,6 (2693,3 ; 4911,8)	-1,3 (-28,8 ; 37,3)	4566,7 (3562,9 ; 5600,0)	6288,4 (5000,2 ; 7703,7)	37,7 ( 6,4 ; 78,1)	4147.7 (3396.3 ; 5038.5)	4901.3 (3813.0 ; 6156.2)	18.2 ( -9.7 ; 52.3)
	Disfunção renal	246,8 (184,3 ; 333,0)	290,4 (200,8 ; 408,5)	17,7 (-14,5 ; 61,2)	339,5 (250,3 ; 455,6)	547,5 (407,3 ; 713,9)	61,3 ( 23,7 ; 108,8)	292.0 (221.2 ; 384.1)	408.8 (302.2 ; 543.5)	40.0 ( 7.2 ; 78.8)
	Baixo nível de atividade física	31,2 ( 10,9 ; 86,8)	35,6 ( 12,3 ; 95,7)	14,0 (-19,7 ; 59,8)	38,3 ( 11,9 ; 109,6)	59,7 ( 18,2 ; 168,5)	55,9 ( 13,5 ; 107,4)	34.9 ( 11.8 ; 96.3)	46.8 ( 15.5 ; 130.9)	34.0 ( 0.9 ; 75.9)
	Temperatura não ideal	149,8 ( 70,6 ; 247,3)	132,2 ( 71,2 ; 216,2)	-11,8 (-39,2 ; 35,3)	194,7 ( 95,0 ; 318,5)	237,2 (131,7 ; 383,7)	21,9 (-10,6 ; 77,9)	171.9 ( 84.7 ; 278.4)	180.8 ( 98.6 ; 290.4)	5.2 (-23.0 ; 56.2)
	Outros riscos ambientais	261,7 (121,8 ; 446,7)	250,6 (125,9 ; 417,4)	-4,2 (-29,7 ; 32,6)	718,6 (493,5 ; 992,0)	755,6 (486,2 ; 1051,0)	5,1 (-19,0 ; 35,2)	478.1 (302.8 ; 698.4)	472.2 (285.2 ; 698.0)	-1.2 (-24.1 ; 26.3)
	Tabagismo	281,5 (206,0 ; 381,2)	259,9 (176,3 ; 362,1)	-7,7 (-39,2 ; 35,4)	1144,0 (886,4 ; 1461,9)	1362,9 (1056,3 ; 1739,2)	19,1 (-11,8 ; 59,9)	693.8 (552.7 ; 858.8)	761.4 (587.0 ; 968.6)	9.7 (-18.3 ; 46.7)
**Portugal**	Todos os fatores de risco	3975,2 (3678,9 ; 4220,2)	1259,7 (1125,8 ; 1379,5)	-68,3 (-70,5 ; -66,0)	6467,6 (6199,8 ; 6737,6)	2307,9 (2164,6 ; 2458,8)	-64,3 (-66,1 ; -62,4)	5089.2 (4813.4 ; 5329.9)	1742.1 (1601.3 ; 1867.5)	-65.8 (-67.4 ; -63.9)
	Poluição do ar	383,5 (140,9 ; 684,6)	58,6 ( 34,2 ; 84,3)	-84,7 (-92,1 ; -63,2)	635,5 (214,7 ; 1135,0)	115,6 ( 67,1 ; 168,5)	-81,8 (-90,8 ; -53,6)	498.6 (173.3 ; 889.3)	85.1 ( 49.3 ; 123.3)	-82.9 (-91.3 ; -58.4)
	Consumo de álcool	156,7 ( 65,6 ; 250,4)	34,5 ( 11,6 ; 59,6)	-78,0 (-87,6 ; -66,2)	817,7 (545,1 ; 1084,0)	244,2 (159,3 ; 335,1)	-70,1 (-75,0 ; -65,3)	434.4 (315.3 ; 557.0)	126.3 ( 85.6 ; 172.2)	-70.9 (-76.6 ; -65.2)
	Riscos dietéticos	1284,8 (1060,2 ; 1551,1)	421,2 (341,8 ; 527,1)	-67,2 (-69,9 ; -63,8)	2418,6 (1986,6 ; 2921,4)	928,3 (751,5 ; 1140,0)	-61,6 (-64,6 ; -58,1)	1799.3 (1477.3 ; 2165.0)	655.6 (535.7 ; 806.8)	-63.6 (-66.1 ; -60.4)
	Índice de massa corporal elevado	826,0 (466,4 ; 1206,6)	327,5 (208,2 ; 459,9)	-60,4 (-65,1 ; -51,2)	1186,0 (563,5 ; 1876,8)	554,2 (305,8 ; 830,1)	-53,3 (-59,7 ; -39,5)	997.8 (527.0 ; 1519.4)	435.5 (255.2 ; 633.1)	-56.4 (-61.4 ; -45.4)
	Glicemia de jejum elevada	835,3 (539,4 ; 1328,9)	370,1 (253,2 ; 541,8)	-55,7 (-69,3 ; -37,9)	1211,2 (832,5 ; 1830,0)	664,7 (475,1 ; 935,0)	-45,1 (-59,1 ; -28,6)	1005.5 (687.6 ; 1530.6)	503.2 (364.3 ; 711.2)	-50.0 (-61.6 ; -36.2)
	Colesterol LDL elevado	1128,6 (811,9 ; 1602,0)	346,0 (247,0 ; 476,9)	-69,3 (-72,0 ; -66,3)	2115,0 (1689,9 ; 2725,8)	750,7 (613,5 ; 924,6)	-64,5 (-67,6 ; -61,4)	1579.1 (1216.6 ; 2105.8)	534.5 (422.8 ; 687.7)	-66.1 (-68.9 ; -63.2)
	Pressão arterial sistólica elevada	2545,2 (2121,7 ; 2988,7)	755,9 (614,3 ; 894,2)	-70,3 (-75,6 ; -64,2)	4088,5 (3569,3 ; 4595,6)	1437,8 (1255,6 ; 1611,7)	-64,8 (-68,7 ; -60,6)	3243.3 (2797.8 ; 3673.3)	1072.5 (938.7 ; 1209.2)	-66.9 (-70.3 ; -62.8)
	Disfunção renal	331,4 (255,8 ; 406,1)	107,3 ( 81,9 ; 134,3)	-67,6 (-70,4 ; -64,7)	472,2 (378,5 ; 570,1)	163,3 (129,3 ; 199,9)	-65,4 (-67,8 ; -62,6)	395.9 (312.8 ; 478.4)	133.4 (105.0 ; 162.6)	-66.3 (-68.6 ; -64.0)
	Baixo nível de atividade física	241,0 ( 96,4 ; 454,2)	79,2 ( 34,1 ; 143,3)	-67,2 (-71,9 ; -59,5)	287,0 ( 86,6 ; 611,0)	105,6 ( 34,2 ; 210,8)	-63,2 (-70,7 ; -52,7)	265.6 ( 98.9 ; 524.9)	92.4 ( 35.7 ; 173.5)	-65.2 (-70.6 ; -58.3)
	Temperatura não ideal	402,4 (331,0 ; 480,2)	111,3 ( 89,0 ; 133,4)	-72,3 (-74,4 ; -70,3)	629,7 (517,6 ; 747,5)	195,3 (158,8 ; 235,2)	-69,0 (-70,9 ; -66,8)	504.2 (414.7 ; 600.5)	150.0 (121.7 ; 179.8)	-70.2 (-72.0 ; -68.3)
	Outros riscos ambientais	163,0 ( 77,1 ; 253,2)	44,3 ( 18,6 ; 72,6)	-72,8 (-77,5 ; -69,2)	439,9 (284,8 ; 596,2)	119,0 ( 67,6 ; 171,9)	-72,9 (-77,6 ; -69,6)	282.9 (169.0 ; 398.2)	76.9 ( 39.9 ; 115.5)	-72.8 (-77.1 ; -69.7)
	Tabagismo	644,0 (564,1 ; 726,7)	167,6 (147,9 ; 189,1)	-74,0 (-77,6 ; -70,0)	2087,3 (1971,8 ; 2207,3)	638,6 (591,6 ; 685,4)	-69,4 (-71,8 ; -67,0)	1285.1 (1205.3 ; 1361.9)	384.2 (355.8 ; 414.3)	-70.1 (-72.5 ; -67.6)
**São Tomé e Príncipe**	Todos os fatores de risco	4903,8 (4146,2 ; 5592,8)	5285,6 (4171,6 ; 6407,3)	7,8 (-14,3 ; 34,7)	4424,0 (3548,5 ; 5303,5)	5220,0 (4291,5 ; 6097,6)	18,0 ( -7,1 ; 49,6)	4655.3 (3913.5 ; 5399.0)	5262.6 (4320.2 ; 6132.4)	13.0 ( -8.0 ; 39.6)
	Poluição do ar	1927,4 (1580,0 ; 2328,9)	1612,4 (1214,5 ; 2072,8)	-16,3 (-36,9 ; 11,7)	1749,2 (1367,1 ; 2168,1)	1628,6 (1240,5 ; 2014,2)	-6,9 (-29,8 ; 23,8)	1838.3 (1482.7 ; 2214.2)	1622.8 (1253.5 ; 2017.9)	-11.7 (-32.2 ; 15.9)
	Consumo de álcool	54,3 (-35,1 ; 156,7)	122,0 ( 0,2 ; 271,2)	124,8 (-1878,3 ; 1478,3)	202,3 ( 87,7 ; 336,6)	317,8 (168,3 ; 499,0)	57,1 (-11,5 ; 224,3)	124.8 ( 38.8 ; 226.5)	216.5 (109.1 ; 349.9)	73.5 (-19.7 ; 372.5)
	Riscos dietéticos	1651,5 (1207,6 ; 2333,8)	1742,7 (1174,3 ; 2546,6)	5,5 (-19,0 ; 34,5)	1720,3 (1250,9 ; 2386,2)	1940,4 (1375,8 ; 2690,4)	12,8 (-11,6 ; 45,3)	1671.0 (1244.1 ; 2325.2)	1840.1 (1297.4 ; 2582.2)	10.1 (-12.4 ; 39.8)
	Índice de massa corporal elevado	1149,6 (634,4 ; 1783,9)	1748,3 (1147,1 ; 2490,6)	52,1 ( 8,2 ; 132,7)	610,1 (217,9 ; 1135,0)	1407,7 (866,1 ; 2120,3)	130,7 ( 51,9 ; 367,7)	889.6 (438.9 ; 1451.8)	1584.0 (1035.0 ; 2249.3)	78.0 ( 28.9 ; 180.7)
	Glicemia de jejum elevada	691,4 (450,7 ; 1043,4)	1163,6 (723,9 ; 1714,4)	68,3 ( 21,0 ; 139,6)	699,6 (424,2 ; 1103,9)	1239,2 (795,4 ; 1801,9)	77,1 ( 28,4 ; 151,8)	685.2 (451.9 ; 1025.6)	1198.8 (779.4 ; 1699.3)	75.0 ( 33.9 ; 136.3)
	Colesterol LDL elevado	888,6 (640,0 ; 1165,8)	1140,1 (795,8 ; 1498,6)	28,3 ( -1,5 ; 65,5)	869,3 (588,7 ; 1186,0)	1198,7 (848,3 ; 1572,6)	37,9 ( 7,1 ; 79,1)	874.7 (622.5 ; 1152.5)	1171.4 (844.4 ; 1517.1)	33.9 ( 6.0 ; 69.4)
	Pressão arterial sistólica elevada	3539,8 (2811,3 ; 4228,3)	3934,8 (3001,7 ; 4860,0)	11,2 (-14,1 ; 44,8)	3045,7 (2333,9 ; 3761,0)	3759,9 (2962,7 ; 4521,8)	23,5 ( -4,0 ; 60,7)	3297.0 (2654.7 ; 3911.1)	3859.2 (3065.5 ; 4637.7)	17.0 ( -6.3 ; 47.6)
	Disfunção renal	393,4 (304,1 ; 483,5)	556,2 (418,5 ; 716,7)	41,4 ( 12,3 ; 81,0)	290,2 (209,9 ; 388,4)	437,6 (323,3 ; 566,3)	50,8 ( 20,5 ; 90,3)	343.4 (259.3 ; 432.0)	500.6 (379.5 ; 629.2)	45.8 ( 19.3 ; 82.2)
	Baixo nível de atividade física	97,0 ( 36,6 ; 212,3)	129,9 ( 51,2 ; 274,9)	33,9 ( 5,2 ; 72,7)	79,8 ( 22,8 ; 194,3)	109,0 ( 31,4 ; 268,5)	36,7 ( 4,9 ; 78,2)	88.6 ( 30.7 ; 202.8)	120.6 ( 41.9 ; 276.0)	36.1 ( 9.4 ; 69.8)
	Temperatura não ideal	16,3 (-55,9 ; 81,4)	21,7 (-12,1 ; 84,2)	33,3 (-278,6 ; 367,3)	15,4 (-47,6 ; 78,9)	22,3 (-13,0 ; 81,8)	45,4 (-384,5 ; 329,1)	15.8 (-53.4 ; 78.8)	22.0 (-13.1 ; 82.7)	39.9 (-334.0 ; 336.1)
	Outros riscos ambientais	159,0 ( 42,7 ; 283,2)	155,8 ( 45,4 ; 289,4)	-2,0 (-24,1 ; 28,9)	200,0 ( 88,9 ; 325,3)	210,9 ( 96,8 ; 347,0)	5,5 (-18,7 ; 34,9)	177.1 ( 66.0 ; 300.8)	182.2 ( 70.6 ; 311.0)	2.9 (-17.5 ; 28.5)
	Tabagismo	154,4 (119,1 ; 196,4)	173,9 (126,4 ; 230,7)	12,6 (-19,9 ; 64,0)	470,5 (354,8 ; 599,5)	615,5 (474,0 ; 768,4)	30,8 ( -1,8 ; 78,6)	306.2 (237.3 ; 377.2)	389.8 (300.0 ; 482.8)	27.3 ( -3.4 ; 72.3)
**Timor-Leste**	Todos os fatores de risco	5245,2 (4237,9 ; 6318,1)	5727,3 (4650,7 ; 6805,8)	9,2 (-16,5 ; 38,6)	5157,0 (3969,3 ; 7028,7)	7221,6 (5334,1 ; 9597,5)	40,0 ( 2,1 ; 77,9)	5205.8 (4272.1 ; 6425.2)	6476.7 (5147.2 ; 7999.9)	24.4 ( -5.1 ; 54.9)
	Poluição do ar	2196,8 (1686,5 ; 2845,8)	1967,9 (1518,9 ; 2435,5)	-10,4 (-34,7 ; 19,2)	2080,5 (1505,1 ; 2932,2)	2410,1 (1650,4 ; 3329,1)	15,8 (-19,6 ; 51,9)	2140.0 (1646.1 ; 2762.8)	2190.6 (1631.0 ; 2828.2)	2.4 (-25.5 ; 33.2)
	Consumo de álcool	-2,7 (-13,5 ; 10,5)	11,9 (-16,3 ; 50,8)	-547,6 (-3649,5 ; 3536,9)	39,0 (-49,8 ; 142,4)	295,6 ( 57,7 ; 583,8)	657,3 (-5045,9 ; 6221,4)	18.9 (-28.1 ; 74.7)	154.3 ( 28.3 ; 318.1)	717.6 (-5256.3 ; 9199.6)
	Riscos dietéticos	2380,6 (1638,0 ; 3255,7)	2418,4 (1677,1 ; 3291,1)	1,6 (-24,7 ; 31,4)	2645,0 (1804,2 ; 3825,0)	3452,2 (2270,2 ; 4875,1)	30,5 ( -6,9 ; 71,7)	2516.4 (1794.0 ; 3435.5)	2936.2 (2011.2 ; 4031.7)	16.7 (-13.8 ; 51.1)
	Índice de massa corporal elevado	380,3 ( 96,7 ; 847,5)	590,8 (211,4 ; 1136,1)	55,4 ( 0,9 ; 210,6)	254,1 ( 43,6 ; 675,6)	668,8 (212,5 ; 1339,0)	163,2 ( 55,4 ; 577,8)	315.6 ( 71.8 ; 766.0)	630.9 (223.9 ; 1210.9)	99.9 ( 30.9 ; 303.3)
	Glicemia de jejum elevada	543,6 (359,3 ; 818,3)	1493,1 (986,7 ; 2191,2)	174,7 ( 94,2 ; 292,1)	597,0 (359,7 ; 941,4)	1832,7 (1179,3 ; 2821,4)	207,0 (110,8 ; 347,6)	569.1 (370.6 ; 852.3)	1659.8 (1121.0 ; 2421.9)	191.6 (113.4 ; 300.2)
	Colesterol LDL elevado	994,8 (710,9 ; 1343,8)	1180,1 (820,5 ; 1595,4)	18,6 (-12,4 ; 55,0)	976,6 (652,3 ; 1471,2)	1447,2 (885,4 ; 2125,4)	48,2 ( 1,6 ; 97,6)	988.2 (704.4 ; 1351.8)	1317.3 (894.6 ; 1800.3)	33.3 ( -4.0 ; 73.7)
	Pressão arterial sistólica elevada	3595,3 (2758,9 ; 4466,5)	3782,6 (2933,5 ; 4685,8)	5,2 (-24,2 ; 39,2)	3442,5 (2548,8 ; 4786,6)	5095,6 (3629,2 ; 6895,6)	48,0 ( 5,8 ; 92,4)	3523.1 (2794.6 ; 4533.6)	4442.1 (3386.4 ; 5642.3)	26.1 ( -6.5 ; 61.4)
	Disfunção renal	502,7 (370,5 ; 653,6)	685,8 (504,1 ; 882,6)	36,4 ( 2,4 ; 75,0)	446,5 (305,9 ; 651,6)	761,5 (504,6 ; 1077,3)	70,5 ( 23,6 ; 121,4)	474.9 (343.5 ; 633.8)	724.4 (519.6 ; 965.7)	52.5 ( 15.9 ; 93.5)
	Baixo nível de atividade física	83,0 ( 29,5 ; 195,3)	102,4 ( 34,9 ; 241,0)	23,4 ( -7,2 ; 61,0)	93,5 ( 26,9 ; 217,3)	138,8 ( 42,0 ; 334,3)	48,5 ( 9,8 ; 91,8)	88.3 ( 28.9 ; 204.7)	120.4 ( 39.1 ; 285.0)	36.3 ( 7.3 ; 68.7)
	Temperatura não ideal	76,8 ( 12,6 ; 147,1)	73,8 ( 24,3 ; 141,0)	-3,9 (-49,4 ; 92,4)	75,7 ( 12,9 ; 149,9)	94,1 ( 30,3 ; 188,7)	24,3 (-35,6 ; 148,0)	76.4 ( 13.7 ; 146.5)	84.0 ( 27.0 ; 164.3)	10.0 (-40.5 ; 113.1)
	Outros riscos ambientais	181,8 ( 50,0 ; 334,9)	199,7 ( 70,9 ; 355,6)	9,8 (-15,7 ; 55,3)	289,1 (141,3 ; 480,4)	384,0 (193,6 ; 622,3)	32,8 ( -3,9 ; 73,2)	236.2 ( 97.4 ; 394.6)	291.8 (131.7 ; 468.7)	23.6 ( -6.1 ; 59.4)
	Tabagismo	607,6 (445,7 ; 793,9)	545,8 (400,2 ; 711,8)	-10,2 (-36,7 ; 24,1)	1775,7 (1295,2 ; 2464,1)	2338,8 (1659,3 ; 3187,1)	31,7 ( -9,1 ; 76,7)	1203.3 (923.6 ; 1583.0)	1445.2 (1053.0 ; 1920.8)	20.1 (-14.0 ; 59.0)

LDL: colesterol lipoproteína de baixa densidade.

**Figura 2:**
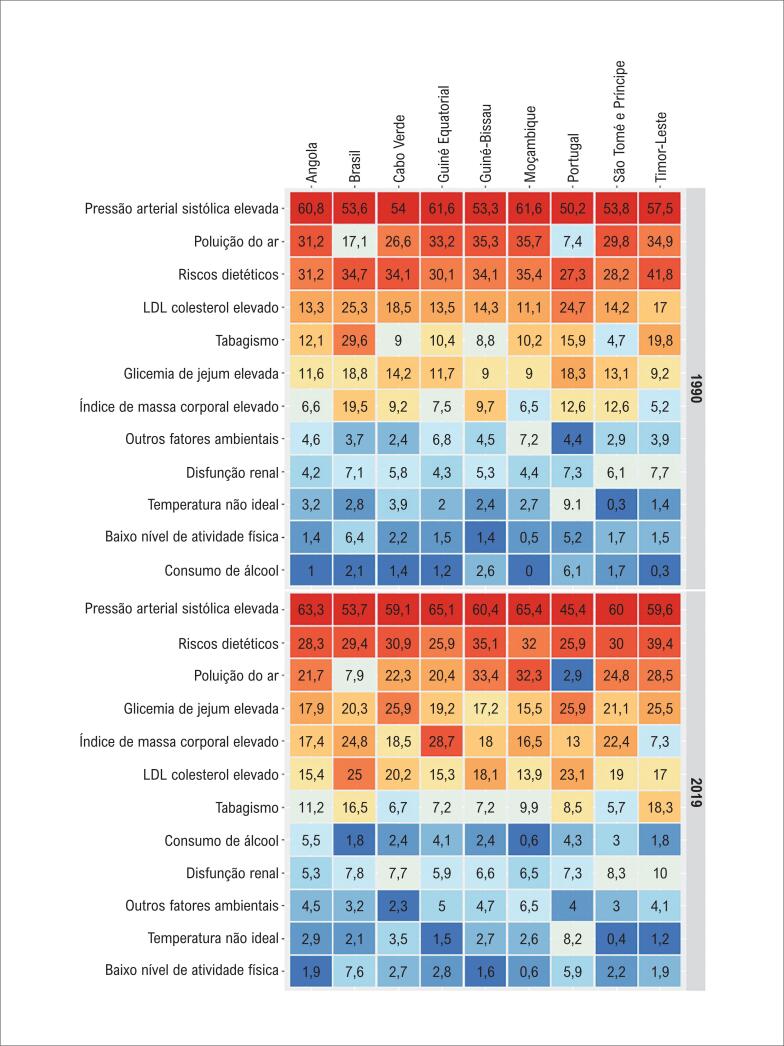
Percentual do total de mortes por doenças cardiovasculares atribuíveis a cada fator de risco cardiovascular, por país de língua portuguesa, em 1990 e 2019.

A Figura 2 apresenta o percentual das mortes por DCVs atribuíveis a cada FR cardiovascular, por país, em 1990 e 2019. A PAS elevada permaneceu com o maior percentual, inclusive com aumento em todos os PLP, exceto Portugal. Observa-se ainda um aumento global da contribuição dos riscos dietéticos entre 1990 e 2019 (do 3° para o 2° lugar), assim como do consumo de álcool, da glicemia de jejum alterada e do IMC elevado. Por outro lado, houve uma redução percentual do colesterol LDL elevado e principalmente do tabagismo, apesar deste último ainda ter contribuição mais expressiva em Portugal, Brasil e Timor Leste.

**Figura 3:**
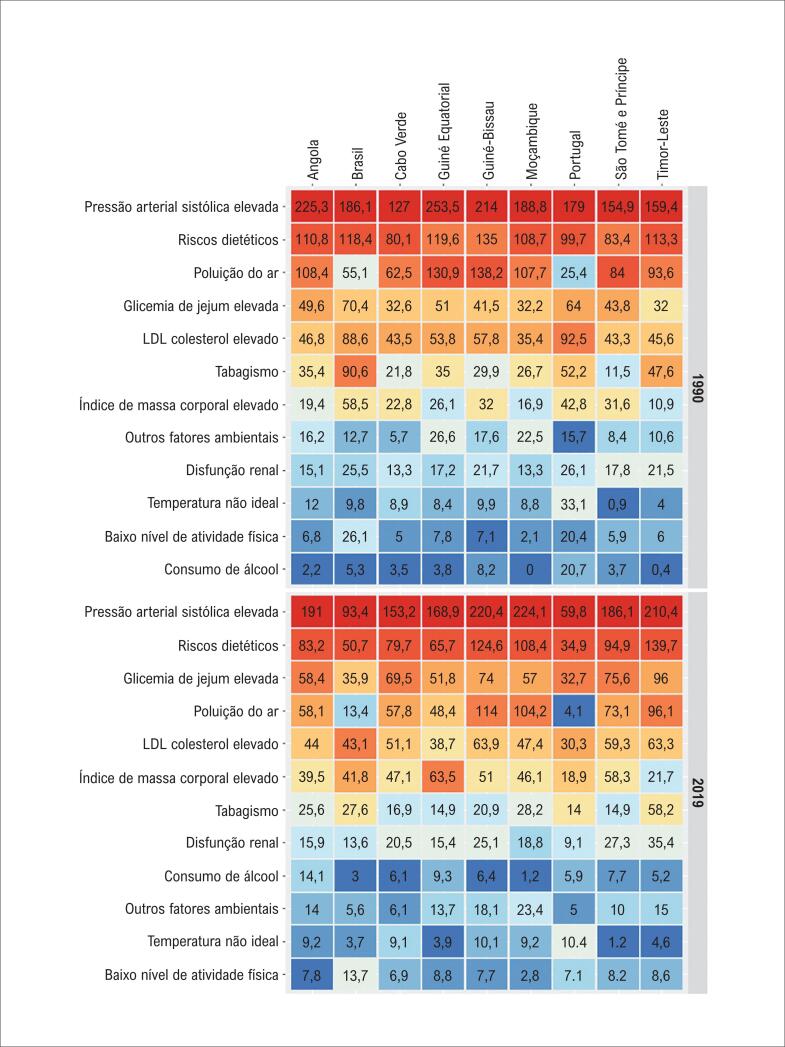
Taxas de mortalidade (por 100 000 habitantes) por doenças cardiovasculares ajustada por idade, atribuíveis a fatores de risco cardiovascular, por país de língua portuguesa, em 1990 e 2019.

Na análise da taxa de mortalidade por DCVs atribuíveis aos FR selecionados, por PLP (Figura 3), observou-se que a PAS elevada ocupou o primeiro lugar em todos os PLP em 1990 e em 2019. Em 1990, as taxas de mortalidade por DCVs por 100 000 habitantes atribuída a PAS foram mais altas em Guiné Equatorial (253.5), Angola (225.3) e Guine Bissau (214.0), enquanto em 2019, essas taxas foram mais altas em Moçambique (224.1), Guiné Bissau (220.4) e Timor Leste (210.4), sendo que as reduções mais expressivas foram observadas em Portugal (-66.6%, II 95% -71.0 - -61.2) e Brasil (-49.8%, II 95% -52.5 - -47.1). Riscos dietéticos, glicemia de jejum elevada, LDL-colesterol elevado e poluição do ar estiveram entre os cinco FR mais importantes na maioria dos PLP em 1990 e em 2019, com exceção às taxas atribuíveis à poluição do ar marcadamente menores no Brasil e em Portugal tanto em 1990 quanto em 2019, com redução mais expressiva nesses países no período. Salienta-se ainda o aumento das taxas de mortalidade atribuíveis ao consumo de álcool em praticamente todos os PLP, exceto Brasil e Portugal, e a redução do tabagismo (também notadamente no Brasil (-69.5%) e Portugal (-73.2%)), apesar das taxas ainda relativamente mais altas em 2019 nesses dois países e no Timor Leste (Figura 3, Tabela 1). A Tabela 2 mostra padrões semelhantes para as taxas de DALYs atribuíveis aos FR para os PLP.

**Figura 4:**
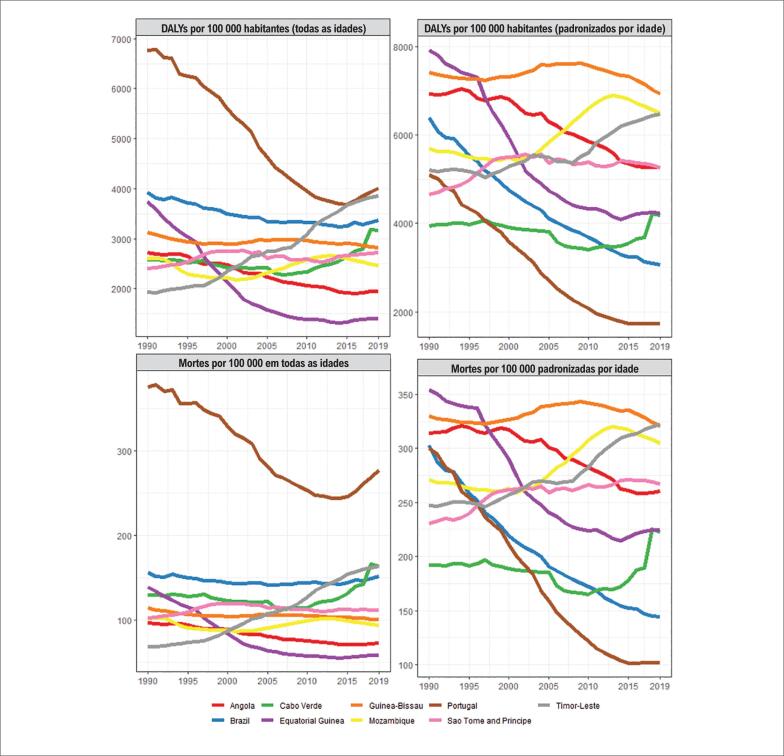
Taxas de mortalidade e anos de vida perdidos por incapacidade (DALYs, Disability-Adjusted Life Years) por doenças cardiovasculares atribuíveis aos fatores de risco cardiovasculares agrupados, nos países de língua portuguesa entre 1990 e 2019.

Avaliando as taxas de mortalidade e DALYs por DCV atribuíveis aos FR cardiovasculares agrupados, na Figura 4, observa-se uma tendência à estabilidade para as taxas brutas entre 1990 e 2019 na maioria dos PLP, com uma tendência decrescente em Portugal e Guiné Equatorial, e ascendente no Timor Leste. Já para as taxas ajustadas por idade, Portugal e Brasil apresentaram forte tendência à redução, contrastando com os demais países, que demonstraram um padrão de relativa estabilidade, ou aumento no caso de Moçambique e Timor Leste (PLP nos limites inferiores do SDI).

**Figura 5:**
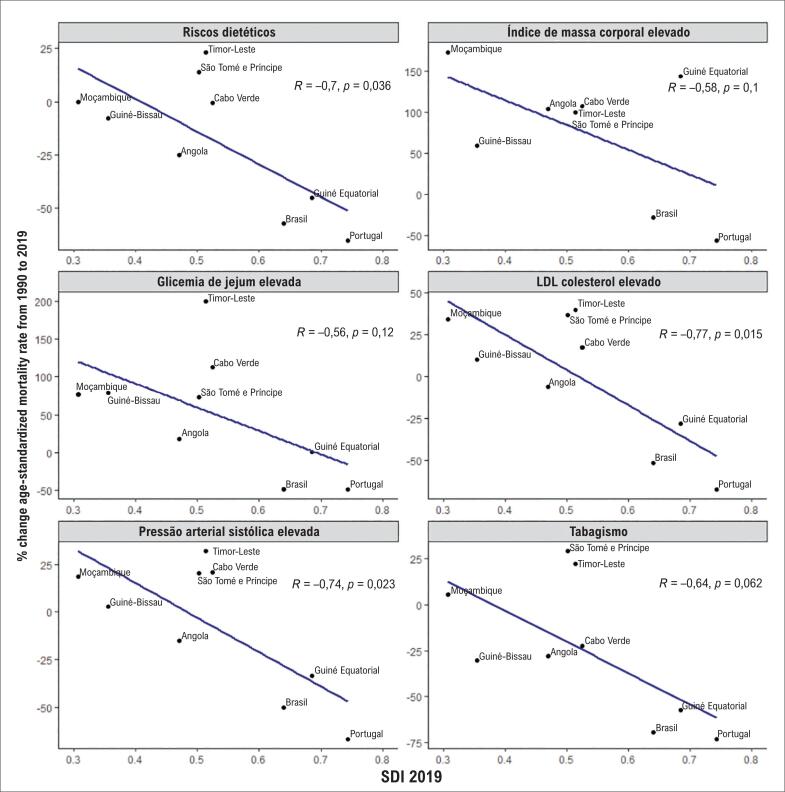
Correlação entre o índice sociodemográfico (SDI, sociodemographic index) e a variação percentual nas taxas de mortalidade por doença cardiovascular atribuível aos fatores de risco, padronizadas por idade, em países de língua portuguesa de 1990 a 2019.

A Figura 5 apresenta o percentual de mudança na taxa de mortalidade atribuível a FR selecionados segundo o SDI em 2019 de cada PLP. Para todos os FR, houve uma tendência à correlação inversa entre SDI e o percentual de mudança, com significância estatística para os riscos dietéticos, LDL colesterol elevado e PAS elevados. Nos três PLP com maiores SDI (Portugal, Guiné Equatorial e Brasil), observou-se redução considerável da mortalidade atribuível a todos os FR, exceto para glicemia de jejum elevada e IMC elevado, que tiveram tendência, respectivamente de estabilidade e aumento apenas na Guiné Equatorial. Para as taxas de SEV padronizadas por idade (Figura S4) o padrão observado foi diferente, com uma tendência à correlação negativa entre variação percentual de taxas de SEV atribuíveis ao tabagismo e SDI, com correlação positiva significativa observada apenas para os fatores dietéticos.

## Discussão

Os PLP partilham laços socioculturais derivados da colonização portuguesa em comum, embora em diferentes graus, e muitas vezes coexistindo com traços de outras culturas participantes do processo de colonização e composição populacional. Existem aproximadamente 280 milhões de falantes da língua portuguesa no mundo (aproximadamente 216 milhões no Brasil), sendo a quinta língua mais falada no mundo, e a mais falada no hemisfério sul.^3^ Os PLP têm realidades socioeconômicas diferentes, desigualdades nos sistemas de saúde, mas etnias semelhantes, os quais são fatores determinantes para as DCVs.^3,6,7^ Nossa análise de FR cardiovasculares nos PLP reforça essa heterogeneidade, demonstrando uma redução mais expressiva das DCVs atribuíveis aos FR em países com sistemas de saúde mais estruturados, e uma estreita relação entre as tendências de mortalidade e SDI, especialmente para os fatores dietéticos, colesterol LDL elevado e PAS elevada.

Um estudo transversal retrospectivo de pacientes nascidos em Portugal, Brasil e África, entre outros, atendidos em clínicas gerais em Lambeth, no sul de Londres, observou que falantes de português (o maior grupo de indivíduos cuja língua de preferência era outro senão inglês) tinham maior probabilidade de apresentar hipertensão (OR=1,43, IC 95% 1,30 - 1,57); diabetes melitus (OR=1,74, IC 95% = 1,50 - 2,02); AVC (OR = 1,40, IC 95% = 1,08-1,81); obesidade (OR=1,53, IC 95%=1,36-1,73); e tabagismo (OR=1,13, IC 95% = 1,02 a 1,25) comparados com os demais grupos étnicos. Os autores discutiram se essas diferenças poderiam ser explicadas pelas barreiras da língua, ou se derivariam de determinantes genéticos comuns, além de (e sobretudo) fatores sociais e culturais.^19^

Em nosso estudo, encontramos que as DCVs atribuíveis aos FR cardiovasculares foram responsáveis por aproximadamente 30% do total das mortes na maioria dos PLP em 2019, embora nos países com SDI menor que 0,5, esse percentual foi inferior a 15%, com exceção da Guiné Equatorial (0,69) que tinha o segundo maior SDI entre os PLP. Tal tendência está associada a uma transição epidemiológica tardia, ou seja, os países com piores marcadores socioeconômicos ainda tendem a apresentar proporções aumentadas dessas doenças e, assim, podem aplicar estratégias de sucesso previamente usadas em outros PLP para tentar mitigar essa tendência.^3^ O percentual atribuível aos FR foi elevado (>75%) em todos os PLP, e a PAS elevada foi o principal fator de risco para DCV no período analisado. Em todos os PLP houve redução das taxas de mortalidade por DCV, padronizadas por idade, atribuídas aos FR no período, especialmente nos países com maior SDI (Portugal, Guiné Equatorial e Brasil). Destaca-se que embora a Guiné Equatorial tenha o maior produto interno bruto per capita no continente Africano, os recursos são distribuídos de forma desigual, beneficiando pouco a população em geral, coexistindo a mortalidade proporcional por doenças crônicas e infecciosas.^6^

Hipertensão, diabetes mellitus, hipercolesterolemia, obesidade e tabagismo foram os cinco principais FR cardiovascular tradicionais modificáveis observados na África em 2019.^4^ Pelo menos um desses cinco FR está presente em 80% a 95% dos indivíduos que sofrem um evento cardiovascular fatal ou não fatal nesse continente.^4,20^ O mesmo foi observado para o Brasil e Portugal,^3,21^ exceto para o tabagismo, que apresentou reduções significativas nesses países nesse período, conforme relatado em outro estudo,^4^ em decorrência de políticas públicas e campanhas de enfrentamento.^4^ Por outro lado, um aumento das taxas de mortalidade por DCV atribuíveis ao consumo de álcool foi observada nos PLP da África e nas Guinés Equatorial e Guiné Bissau, provavelmente refletindo a tendência mundial de aumento do consumo de álcool com impacto nas DCV.^22^

Ressalta-se que as taxas de mortalidade por DCV atribuível à PAS elevada permaneceram no primeiro lugar em todos os PLP entre 1990 e 2019. Como mencionado, reduções mais expressivas foram observadas em Portugal e no Brasil,^3,21^ provavelmente associadas com os maiores SDIs, mas também com medidas populacionais para a redução da ingestão de sal, especialmente em Portugal, onde observou-se redução do infarto do miocárdio e AVC atribuíveis à PAS elevada.^21^ Esses dados denotam uma mudança no perfil dos países com maiores taxas de mortalidade por DCV atribuída a PAS, com declínio naqueles com melhores índices socioeconômicos e transição epidemiológica mais precoce, com tendência inversa nos de menor SDI.

Os PLP apresentaram incremento dos riscos dietéticos e fatores metabólicos atribuíveis à mortalidade por DCVs. O estudo “*Prospective Urban Rural Epidemiology*” (PURE), realizado em 21 países, com 148 858 participantes e seguimento médio de 9,5 anos, demonstrou que ingestões mais alta de grãos refinados, que representaram 70% da ingestão energética dos países da África, foram associados a maior PAS, e maior risco de mortalidade total e por DCVs.^23^ Em nossa análise, os riscos dietéticos, associados a glicemia de jejum e LDL-colesterol elevados, estiveram entre os cinco FR mais importantes na maioria dos PLP em 1990 e em 2019, e estiveram correlacionados com os PLP com menor SDI. Esses achados foram também observados em um sub-estudo do GBD que analisou a mortalidade e a carga de doenças associados com DCVs no mundo.^2^

Um estudo que analisou a carga de DCV para 194 países do mundo, de 1990 a 2019, demostrou tendência de queda dos DALYs, YLL e YLD, com taxas mais altas de YLD nas mulheres em comparação com os homens,^24^ com o mesmo ocorrendo em relação à carga de DCV atribuível aos FR cardiovasculares.^4^ Esses dados ressaltam a heterogeneidade dos PLP em relação à mortalidade e carga de doença, cujas variações não podem ser explicadas somente pelo SDI, com potencial contribuição de múltiplos fatores, como sexo, etnia e até mesmo diferenças culturais e ambientais.

O estudo PURE sugeriu que uma grande proporção de mortes prematuras por DCVs poderiam ser evitadas diminuindo alguns FR modificáveis com políticas globais, tais como controle da hipertensão e do tabagismo e melhoria da educação em saúde.^25^ O impacto da redução de outros FR como riscos dietéticos e poluição ambiental pode variar de acordo com nível socioeconômico de cada país, e com o desenvolvimento das regulamentações internas das atividades econômicas (como a emissão de poluentes e reparo de danos ambientais).^26,27^ Desta forma, estratégias para o enfrentamento das mortes e da carga de DCV nos PLP poderiam focar, neste momento, nos FR mais prevalentes, com medidas populacionais de baixo custo e alto impacto, como a redução de consumo dietético de sal e calorias da dieta, diminuição do tabagismo e consumo de álcool, e controle da PAS.

### Limitações e pontos fortes do estudo

Limitações relacionadas à metodologia do estudo GBD foram previamente detalhadas,^4,7^ e se relacionam principalmente à heterogeneidade das fontes primárias dos dados entre os PLP, dados estatísticos completos de mortalidade e limitações da extrapolação de estimativas para territórios com baixa qualidade de dados – condição observada para alguns PLP. Tem havido melhora progressiva na completude dos dados de prevalência e morbidade; entretanto, a integridade e a qualidade para alguns PLP ainda são limitadas, de acordo com dados do GBD 2019.^4^ Como exemplo, citam-se índices muito baixos ou dados inexistentes sobre mortalidade em PLP da África subsaariana.^7,8^ É possível ter também ocorrido uma inadequação dos modelos do estudo GBD para os diferentes países em alguns grupos de doenças sujeitos a menor vigilância epidemiológica, principalmente os FR cardiovasculares não notificáveis. Ademais, para alguns FR, não há inquéritos ou programas de saúde específicos em vários PLP. Especificamente sobre estimativas para os FR, o GBD 2019 assume uma distribuição uniforme dos RR em todos os países, para uma mesma idade e sexo,^4^ o que pode aumentar a incerteza dos resultados. Os estudos primários, quando existentes, reportam dados de prevalência como uma medida de exposição a um fator de risco, o que limita a comparabilidade com as medidas de exposição de risco (SEV) do GBD. Ademais, a metodologia do GBD desconsidera FR distais, que podem ser mediadores da prevalência e mortalidade dos FR intermediários, afetando os seus efeitos enquanto determinantes sociais de saúde.^28,29^ Outro aspecto metodológico é a limitação da modelagem para coexistência de FR simultâneos, que sabidamente resulta em risco superior à soma de fatores individuais (p.ex.: hipertensão, na presença de diabetes e tabagismo, potencializando a doença isquêmica do coração).^2,30^ Adicionalmente, a metodologia de ajuste dos FR para definições padronizadas aplicada pelo GBD pode ser uma fonte adicional de viés.^4,15^ Finalmente, a despeito da colonização similar, a heterogeneidade sociocultural, demográfica, econômica e étnica dos PLP – influenciando hábitos de vida, comportamentos de saúde, conhecimento e controle dos FR – pode não ser adequadamente capturada pelos modelos analíticos.^6^

Entretanto, apesar dessas limitações, o GBD constitui-se em uma metodologia robusta, abrangente e validada do ponto de vista epidemiológico para a estimação da carga de doença atribuível aos FR cardiovasculares, pela produção de métricas comparáveis entre os PLP – inclusive aqueles com escassez ou inexistência de dados primários. Além disso, diante da realidade dos sistemas de saúde locais, nossos achados podem auxiliar na reformulação de políticas de saúde.

## Conclusões

O conjunto de 12 FR cardiovasculares incluídos nesta análise do GBD 2019 são responsáveis por mais de 75% da carga de DCVs nos nove PLP, com um maior impacto dessas doenças sobre a mortalidade em Portugal, Timor Leste, Cabo Verde e Brasil. A PAS elevada permaneceu como principal fator de risco para mortalidade e DALYs por DCVs entre 1990 e 2019. Houve uma redução expressiva das taxas de mortalidade cardiovascular padronizadas por idade atribuíveis aos FR, notadamente nos PLP com melhores índices socioeconômicos, como Brasil, Portugal e Guiné Equatorial. Em geral, tem havido um impacto crescente dos FR dietéticos e metabólicos, em paralelo com redução de taxas de tabagismo na maioria dos PLP. Além disso, observou-se uma correlação negativa marcante entre a variação das taxas de mortalidade por DCVs atribuíveis aos FR e o SDI. Esses resultados mostram a heterogeneidade entre os PLP em relação à epidemiologia dos FR avaliados, sugerindo a necessidade de políticas de saúde e ações governamentais adaptadas à realidade de cada país, e da colaboração entre essas nações para reduzir o impacto das DCVs.

Estes dados podem ajudar os países a identificar problemas comuns, sendo um estímulo importante para a troca de experiências entre pesquisadores e comunidades acadêmicas. Os PLP devem avançar neste engajamento e solidariedade entre eles,^31^ em especial aqueles com mais recursos e capacidades técnicas, apoiando os processos de formação de recursos humanos e parcerias.

## *Material suplementar

Para informação adicional, por favor, clique aqui.
