# Supplementary material for: Carga de Doenças Cardiovasculares Atribuível aos Fatores de Risco nos Países de Língua Portuguesa: Dados do Estudo “Global Burden of Disease 2019”
Source: Arq Bras Cardiol. 2022 Jun 6;118(6):1028–48. [Article in Portuguese] doi: 10.36660/abc.20210680 (PMC9345142; doi:10.36660/abc.20210680)
Supplement: Translation

# Translation: Burden of Cardiovascular Diseases Attributable to Risk Factors in Portuguese-Speaking Countries: Data from the “Global Burden of Disease 2019” Study

**Authors:** Bruno Ramos Nascimento, Luisa Campos Caldeira Brant, André Dias Nassar Naback, Guilherme Augusto Veloso, Carisi Anne Polanczyk, Antonio Luiz Pinho Ribeiro, Deborah Carvalho Malta, Albano Vicente Lopes Ferreira, Gláucia Maria Moraes de Oliveira

**Affiliations:** 1 Belo Horizonte MG Brazil Faculdade de Medicina e Hospital das Clínicas, Universidade Federal de Minas Gerais, Belo Horizonte, MG – Brazil; 2 Belo Horizonte MG Brazil Programa de Pós-Graduação em Estatística, Departamento de Estatística, Universidade Federal de Minas Gerais, Belo Horizonte, MG – Brazil; 3 Porto Alegre RS Brazil Instituto Nacional de Avaliação de Tecnologias em Saúde, IATS/CNPq, Porto Alegre, RS – Brazil; 4 Porto Alegre RS Brazil Faculdade de Medicina - Universidade Federal do Rio Grande do Sul, Porto Alegre, RS – Brazil; 5 Porto Alegre RS Brazil Hospital Moinhos de Vento, Porto Alegre, RS – Brazil; 6 Belo Horizonte MG Brazil Escola de Enfermagem, Universidade Federal de Minas Gerais, Belo Horizonte, MG – Brazil; 7 Benguela Angola Faculdade de Medicina, Universidade Katyavala Bwila, Benguela – Angola; 8 Rio de Janeiro RJ Brazil Universidade Federal do Rio de Janeiro, Rio de Janeiro, RJ – Brazil

**Keywords:** Cardiovascular Disease, Risk Factors, Global Burden of Disease, Epidemiology, Community of Portuguese-Speaking Countries

## Abstract

**Background:**

The impact of risk factors (RF) on morbidity and mortality from cardiovascular disease (CVD) for most Portuguese-speaking countries (PSC) is little known.

**Objectives:**

We aimed to analyze the morbidity and mortality from CVD attributable to RF and its variation, from 1990 to 2019, in PSC, based on estimates from the Global Burden of Disease (GBD) 2019 study.

**Methods:**

We evaluated changes in cardiovascular RF, mortality rates and age-standardized disability-adjusted life years (DALYs) between 1990 and 2019. The correlation between percentage changes in mortality rates and the sociodemographic index (SDI) of each PSC was evaluated by the Spearman method. A p-value <0.05 was considered statistically significant.

**Results:**

Elevated systolic blood pressure (SBP) was the main RF for mortality and DALYs for CVD for all PSC. Mortality from CVD showed a downward trend in 2019, more accentuated in Portugal (-66.6%, 95%CI -71.0 - -61.2) and in Brazil (-49.8%, 95%CI -52.5 - -47.1). There was a trend towards an inverse correlation between SDI and the percent change in mortality, which was significant for dietary risks (r=-0.70, p=0.036), high LDL cholesterol (r=-0.77, p=0.015) and high SBP (r=-0.74, p=0.023).

**Conclusions:**

In addition to SBP, dietary and metabolic RF justified a greater variation in the burden of CVD correlated with SDI in the PSC, suggesting the need to adopt health policies adapted to the reality of each country, aiming to reduce their impact on population.

## Introduction

Cardiovascular diseases (CVDs) are the leading causes of death worldwide, although they are not yet the main cause in many low- and middle-income countries, where the epidemiological transition occurred later.^1^ However, with the control of infectious and maternal and child diseases, in addition to the increase in life expectancy and urbanization, the importance of CVDs tends to grow even in these countries, requiring adaptation of health systems. Many of these countries already show an increase in the proportion of CVDs in the total number of all-cause deaths.^2,3^

In addition, CVDs have had a significant impact on morbidity, as an important cause of disability and, consequently, loss of healthy life-years.^2,4^ In order to establish CVD control and prevention strategies, it is essential to determine the main cardiovascular risk factors (RF) and their prevalence. Hypertension and dietary factors continue to be the main RF for CVDs in the world.^4,5^ However, in recent years, other factors have played a greater role in the development of CVDs, such as high body mass index (BMI), high fasting plasma glucose and LDL-cholesterol, alcohol use, and renal disease.^4^

Portuguese-speaking countries (PSC) have been culturally influenced by Portugal in different levels, with the type of colonization and political-economic models being important determinants of heterogeneity among them.^6^ Despite several sociocultural similarities, they are countries with different socioeconomic backgrounds, which has a direct impact on the pattern and temporal trends of the disease burden. Data presented in a study^3^ of trends in morbidity and mortality from CVDs showed differences in the relative importance of CVD burden in these countries. However, the most relevant RF attributable to CVD (hypertension and dietary factors) are shared among most PSC.^4^ A detailed analysis of these data may provide information exchange between the countries, regarding successful actions to fight CVD, especially related to the control of the main RF and reduction of their impact on cardiovascular morbidity and mortality.

The “*Global Burden of Disease Study*” (GBD) is an important observational epidemiological study that uses morbidity and mortality metrics related to the main diseases and risk factors at global, national, and regional levels. One of the GBD’s objectives is to understand, through the assessment of trends, the changes in the profile of diseases that affect populations in the 21st century, and serve as a tool for decision-making in the development of health policies.^4,7^ The aim of this study was to analyze the trends of cardiovascular RF and the burden of CVDs attributable to these RF from 1990 to 2019 in PSC, based on the estimates of the GBD 2019 study by the Institute of Health Metrics and Evaluation (IHME).^8^

## Methods

### Portuguese-speaking countries

PSC are those officially members of the Community of Portuguese Language Countries: Angola, Brazil, Cape Verde, Guinea-Bissau, Mozambique, Portugal and São Tomé and Príncipe, East Timor and Equatorial Guinea.^6^ Equatorial Guinea, originally a Portuguese colony, has three official languages (Spanish, French and Portuguese), and is the most recent member of the Community, since 2014. Considering the Portuguese influence – with different magnitudes – on sociocultural characteristics, habits, health behaviors, and on the organization of health systems, contrasting with the heterogeneity in socioeconomic development, we considered relevant the study of cardiovascular RF in the group of PSC.

### Attributable burden estimates and exposure to RF

GBD uses a hierarchical list of RF that are analyzed at four levels. Level 1 stratifies the RF into three groups: metabolic, behavioral and environmental RF. Level 1 FR are detailed at level 2, resulting in 20 FR. Levels 3 and 4 go further into the detail and, at total, the GBD study analyzed 87 RF in 2019.^4^ In the current study, we analyzed 12 RF, as shown in Table 1. The choice for this group of RF was due to its more robust and well established epidemiological association in the literature, with the burden of disease and mortality from CVD, objects of this study.

**Table 1:** Age-standardized mortality rates

Country	Risk Factor	Female			Male			Both		
		1990	2019	Percent Change %	1990	2019	Percent Change %	1990	2019	Percent Change %
**Angola**	All risk factors	287.8 (201.2 ; 369.9)	245.7 (200.3 ; 300.0)	-14.6 (-37.4 ; 21.5)	338.1 (257.8 ; 411.9)	274.3 (231.3 ; 327.8)	-18.9 (-37.2 ; 11.9)	314.4 (255.6 ; 375.7)	260.2 (219.5 ; 310.4)	-17.2 (-34.3 ; 6.1)
	Air pollution	94.2 ( 58.6 ; 133.9)	51.3 ( 36.5 ; 69.0)	-45.5 (-63.0 ; -16.2)	122.0 ( 91.7 ; 156.3)	66.0 ( 49.5 ; 85.3)	-45.9 (-60.8 ; -22.3)	108.4 ( 82.6 ; 140.8)	58.1 ( 43.1 ; 75.7)	-46.4 (-61.2 ; -27.3)
	Alcohol use	-0.1 ( -4.3 ; 4.2)	8.7 ( 2.4 ; 16.4)	-11607.2 (-5407.9 ; 5127.9)	4.5 ( -2.8 ; 13.0)	21.3 ( 12.4 ; 31.2)	367.8 (-3677.7 ; 4350.4)	2.2 ( -2.5 ; 6.9)	14.1 ( 8.4 ; 21.3)	555.0 (-4697.5 ; 4693.6)
	Dietary risks	94.2 ( 59.0 ; 141.1)	74.6 ( 50.1 ; 112.6)	-20.8 (-44.1 ; 16.8)	126.7 ( 91.0 ; 174.1)	92.8 ( 67.3 ; 131.2)	-26.7 (-45.7 ; 3.1)	110.8 ( 81.0 ; 154.2)	83.2 ( 58.6 ; 120.5)	-24.9 (-43.4 ; -0.5)
	High body mass index	19.2 ( 4.1 ; 46.4)	40.1 ( 19.8 ; 64.9)	108.5 ( 17.6 ; 551.7)	19.2 ( 3.7 ; 48.5)	38.0 ( 17.7 ; 62.7)	97.8 ( 12.6 ; 533.9)	19.4 ( 4.0 ; 47.3)	39.5 ( 19.6 ; 63.8)	103.6 ( 20.2 ; 517.9)
	High fasting plasma glucose	33.2 ( 17.6 ; 59.3)	42.4 ( 23.6 ; 70.1)	28.0 (-22.5 ; 116.4)	67.8 ( 41.9 ; 102.0)	79.6 ( 51.9 ; 115.9)	17.3 (-18.7 ; 85.7)	49.6 ( 32.2 ; 75.4)	58.4 ( 37.8 ; 87.3)	17.8 (-14.6 ; 71.0)
	High LDL cholesterol	40.9 ( 24.9 ; 63.3)	40.8 ( 25.0 ; 60.4)	-0.2 (-32.5 ; 46.7)	51.8 ( 35.4 ; 73.3)	46.8 ( 31.4 ; 66.4)	-9.7 (-34.4 ; 28.5)	46.8 ( 32.1 ; 66.3)	44.0 ( 28.6 ; 62.8)	-6.0 (-31.5 ; 26.1)
	High systolic blood pressure	216.1 (153.1 ; 284.2)	188.7 (148.2 ; 236.2)	-12.7 (-37.4 ; 27.4)	230.7 (171.4 ; 291.4)	188.9 (154.6 ; 230.9)	-18.1 (-38.1 ; 15.3)	225.3 (178.2 ; 279.6)	191.0 (156.9 ; 233.6)	-15.2 (-33.8 ; 10.8)
	Kidney dysfunction	13.4 ( 8.2 ; 20.4)	15.0 ( 10.3 ; 20.8)	11.4 (-20.0 ; 64.3)	16.6 ( 11.2 ; 22.9)	16.7 ( 12.1 ; 22.3)	0.3 (-25.0 ; 39.7)	15.1 ( 10.4 ; 20.9)	15.9 ( 11.3 ; 21.6)	5.1 (-20.1 ; 37.9)
	Low physical activity	8.0 ( 3.1 ; 16.6)	9.0 ( 3.8 ; 18.0)	12.8 (-20.7 ; 67.8)	5.2 ( 1.4 ; 13.0)	5.7 ( 1.8 ; 14.1)	10.4 (-20.5 ; 64.7)	6.8 ( 2.6 ; 15.1)	7.8 ( 3.0 ; 16.4)	14.3 (-16.9 ; 56.3)
	Non-optimal temperature	11.1 ( 5.3 ; 17.9)	8.8 ( 5.1 ; 13.4)	-20.9 (-45.7 ; 31.0)	12.8 ( 6.8 ; 20.5)	9.5 ( 5.7 ; 14.9)	-25.6 (-45.0 ; 10.4)	12.0 ( 6.2 ; 18.6)	9.2 ( 5.5 ; 14.1)	-23.6 (-42.7 ; 11.3)
	Other environmental risks	12.3 ( 4.7 ; 23.2)	11.6 ( 5.4 ; 19.7)	-6.2 (-32.9 ; 45.1)	20.2 ( 10.9 ; 31.6)	17.2 ( 10.1 ; 25.6)	-14.8 (-35.6 ; 19.2)	16.2 ( 8.1 ; 26.6)	14.0 ( 7.6 ; 21.8)	-13.4 (-31.9 ; 13.9)
	Smoking	13.1 ( 8.6 ; 18.3)	10.9 ( 7.9 ; 14.6)	-16.4 (-44.1 ; 28.2)	58.3 ( 43.6 ; 73.5)	43.8 ( 34.9 ; 55.4)	-24.9 (-45.5 ; 6.2)	35.4 ( 27.6 ; 43.9)	25.6 ( 20.2 ; 32.5)	-27.9 (-46.2 ; -1.5)
**Brazil**	All risk factors	259.5 (237.7 ; 274.8)	118.2 (103.9 ; 128.2)	-54.4 (-57.0 ; -52.2)	352.0 (331.8 ; 368.2)	175.8 (161.3 ; 187.3)	-50.0 (-53.2 ; -47.4)	303.1 (282.6 ; 317.6)	144.3 (130.8 ; 153.5)	-52.4 (-54.5 ; -50.6)
	Air pollution	46.3 ( 34.4 ; 60.2)	10.8 ( 7.8 ; 13.9)	-76.7 (-83.5 ; -67.8)	65.0 ( 46.7 ; 84.7)	16.6 ( 11.9 ; 21.8)	-74.5 (-82.1 ; -64.2)	55.1 ( 40.4 ; 71.9)	13.4 ( 9.8 ; 17.6)	-75.6 (-82.9 ; -66.0)
	Alcohol use	-0.1 ( -1.7 ; 1.6)	0.2 ( -0.6 ; 1.2)	-315.7 (-576.3 ; 511.4)	11.5 ( 5.8 ; 18.0)	6.5 ( 3.2 ; 9.9)	-43.7 (-60.0 ; -19.6)	5.3 ( 2.4 ; 8.5)	3.0 ( 1.4 ; 4.8)	-43.5 (-62.5 ; -11.6)
	Dietary risks	95.1 ( 74.8 ; 121.0)	38.4 ( 29.3 ; 50.7)	-59.6 (-63.8 ; -55.9)	144.9 (115.3 ; 181.6)	65.7 ( 50.4 ; 84.1)	-54.7 (-59.0 ; -50.5)	118.4 ( 94.6 ; 148.9)	50.7 ( 39.2 ; 65.7)	-57.2 (-60.9 ; -53.9)
	High body mass index	54.7 ( 32.2 ; 81.1)	36.2 ( 25.4 ; 48.4)	-33.9 (-43.7 ; -16.7)	62.1 ( 32.4 ; 98.6)	47.9 ( 30.4 ; 66.8)	-22.8 (-35.9 ; 6.2)	58.5 ( 32.7 ; 89.7)	41.8 ( 28.1 ; 56.8)	-28.5 (-38.8 ; -8.6)
	High fasting plasma glucose	58.1 ( 38.4 ; 88.3)	27.2 ( 18.0 ; 41.6)	-53.2 (-59.3 ; -46.1)	85.4 ( 58.1 ; 128.3)	47.1 ( 32.1 ; 68.6)	-44.9 (-50.4 ; -37.8)	70.4 ( 47.4 ; 106.1)	35.9 ( 24.5 ; 53.0)	-49.0 (-53.4 ; -43.9)
	High LDL cholesterol	72.9 ( 54.2 ; 97.8)	33.8 ( 25.0 ; 45.1)	-53.7 (-56.9 ; -50.4)	105.7 ( 82.4 ; 133.6)	54.2 ( 42.3 ; 68.4)	-48.8 (-52.1 ; -44.9)	88.6 ( 67.8 ; 114.8)	43.1 ( 33.4 ; 55.9)	-51.3 (-53.8 ; -48.6)
	High systolic blood pressure	161.6 (140.1 ; 182.2)	76.8 ( 64.8 ; 87.4)	-52.5 (-56.0 ; -49.0)	212.6 (187.4 ; 236.0)	113.0 ( 98.4 ; 126.1)	-46.8 (-50.3 ; -43.3)	186.1 (163.8 ; 206.7)	93.4 ( 80.2 ; 104.2)	-49.8 (-52.5 ; -47.1)
	Kidney dysfunction	21.9 ( 16.9 ; 27.2)	11.0 ( 8.6 ; 13.7)	-49.7 (-53.4 ; -46.4)	29.3 ( 23.1 ; 35.8)	16.8 ( 13.2 ; 20.5)	-42.9 (-47.0 ; -38.5)	25.5 ( 19.9 ; 31.3)	13.6 ( 10.8 ; 16.7)	-46.5 (-49.6 ; -43.4)
	Low physical activity	25.1 ( 13.1 ; 38.0)	12.3 ( 7.4 ; 17.9)	-50.9 (-56.7 ; -39.7)	27.0 ( 11.4 ; 45.7)	15.4 ( 7.9 ; 24.5)	-42.9 (-50.3 ; -24.9)	26.1 ( 12.6 ; 41.4)	13.7 ( 7.6 ; 20.8)	-47.6 (-53.6 ; -35.0)
	Non-optimal temperature	8.7 ( 2.0 ; 13.7)	3.1 ( 0.9 ; 4.8)	-64.0 (-78.6 ; -27.2)	11.0 ( 1.4 ; 17.5)	4.4 ( 0.9 ; 6.7)	-60.3 (-83.9 ; -5.5)	9.8 ( 1.8 ; 15.4)	3.7 ( 0.8 ; 5.6)	-62.3 (-78.6 ; -19.0)
	Other environmental risks	8.7 ( 3.3 ; 14.5)	3.9 ( 1.5 ; 6.7)	-55.7 (-59.4 ; -51.1)	17.5 ( 9.7 ; 25.5)	7.8 ( 4.1 ; 11.9)	-55.3 (-60.2 ; -51.4)	12.7 ( 6.2 ; 19.5)	5.6 ( 2.6 ; 8.9)	-56.1 (-60.0 ; -52.8)
	Smoking	68.4 ( 60.9 ; 76.3)	19.9 ( 17.9 ; 22.1)	-70.8 (-74.3 ; -67.1)	115.8 (108.7 ; 122.4)	36.9 ( 33.8 ; 39.7)	-68.1 (-70.7 ; -65.5)	90.6 ( 84.6 ; 96.5)	27.6 ( 25.5 ; 29.7)	-69.5 (-72.0 ; -67.1)
**Cabo Verde**	All risk factors	163.7 (142.5 ; 184.5)	184.7 (152.9 ; 212.8)	12.8 ( -5.0 ; 33.2)	233.8 (204.9 ; 257.4)	274.6 (245.8 ; 303.6)	17.4 ( 3.5 ; 34.8)	192.1 (171.0 ; 209.8)	222.6 (193.8 ; 247.8)	15.9 ( 1.6 ; 32.0)
	Air pollution	51.0 ( 41.4 ; 62.5)	44.5 ( 34.8 ; 55.0)	-12.7 (-32.8 ; 13.7)	79.1 ( 66.8 ; 94.3)	75.6 ( 60.3 ; 90.9)	-4.4 (-23.6 ; 16.4)	62.5 ( 52.7 ; 74.6)	57.8 ( 46.6 ; 69.3)	-7.5 (-27.0 ; 15.3)
	Alcohol use	1.5 ( -1.0 ; 4.5)	1.9 ( -1.2 ; 5.5)	22.7 (-692.9 ; 1213.6)	6.2 ( 1.4 ; 11.2)	11.9 ( 5.4 ; 19.6)	93.5 ( -1.3 ; 463.0)	3.5 ( 0.7 ; 6.5)	6.1 ( 2.1 ; 10.8)	75.1 (-33.1 ; 490.7)
	Dietary risks	62.9 ( 48.6 ; 82.7)	64.1 ( 46.1 ; 89.3)	1.8 (-16.2 ; 22.4)	105.5 ( 82.9 ; 133.2)	101.1 ( 76.7 ; 134.8)	-4.2 (-18.4 ; 13.1)	80.1 ( 63.1 ; 101.9)	79.7 ( 59.4 ; 108.0)	-0.5 (-15.6 ; 15.9)
	High body mass index	24.3 ( 11.8 ; 39.0)	41.4 ( 26.5 ; 60.0)	70.3 ( 24.0 ; 174.8)	20.8 ( 7.1 ; 38.9)	53.3 ( 32.3 ; 79.4)	156.0 ( 80.8 ; 427.3)	22.8 ( 9.8 ; 38.8)	47.1 ( 29.7 ; 68.4)	107.1 ( 55.0 ; 249.1)
	High fasting plasma glucose	26.7 ( 15.4 ; 45.0)	59.1 ( 34.8 ; 92.6)	121.1 ( 57.8 ; 219.5)	41.5 ( 24.8 ; 69.3)	85.3 ( 54.4 ; 125.9)	105.5 ( 47.8 ; 189.0)	32.6 ( 19.4 ; 53.9)	69.5 ( 43.1 ; 106.5)	113.3 ( 61.1 ; 187.2)
	High LDL cholesterol	34.9 ( 23.2 ; 48.0)	43.4 ( 28.2 ; 61.3)	24.3 ( 2.9 ; 49.5)	56.5 ( 39.2 ; 76.3)	61.0 ( 41.8 ; 82.9)	8.0 ( -7.1 ; 28.2)	43.5 ( 30.0 ; 59.2)	51.1 ( 34.7 ; 69.9)	17.4 ( 1.0 ; 35.2)
	High systolic blood pressure	111.5 ( 90.3 ; 134.6)	127.6 ( 98.1 ; 155.9)	14.4 (-10.7 ; 44.5)	149.7 (123.5 ; 177.1)	186.9 (156.5 ; 219.0)	24.9 ( 5.9 ; 48.5)	127.0 (106.9 ; 148.8)	153.2 (125.6 ; 179.6)	20.7 ( 2.9 ; 41.8)
	Kidney dysfunction	11.2 ( 7.7 ; 15.1)	17.7 ( 12.3 ; 23.4)	58.4 ( 29.2 ; 96.6)	16.4 ( 10.9 ; 22.3)	24.4 ( 17.4 ; 31.7)	49.0 ( 26.4 ; 79.6)	13.3 ( 9.0 ; 17.9)	20.5 ( 14.7 ; 26.8)	54.6 ( 31.5 ; 85.0)
	Low physical activity	4.7 ( 1.7 ; 10.2)	6.7 ( 2.6 ; 14.1)	43.6 ( 16.1 ; 85.9)	5.5 ( 1.5 ; 14.5)	7.0 ( 2.1 ; 17.0)	26.3 ( -1.4 ; 74.0)	5.0 ( 1.7 ; 11.8)	6.9 ( 2.5 ; 15.9)	37.5 ( 13.5 ; 77.5)
	Non-optimal temperature	7.5 ( -0.0 ; 15.6)	7.7 ( 2.0 ; 16.1)	2.3 (-33.1 ; 63.5)	11.0 ( 0.6 ; 22.5)	11.0 ( 2.5 ; 23.3)	0.0 (-39.7 ; 68.0)	8.9 ( 0.2 ; 18.3)	9.1 ( 2.2 ; 19.1)	1.9 (-31.6 ; 66.1)
	Other environmental risks	4.5 ( 1.1 ; 8.3)	4.8 ( 1.3 ; 8.7)	6.3 (-13.9 ; 42.5)	7.5 ( 2.6 ; 12.9)	8.0 ( 2.8 ; 14.0)	7.0 (-10.4 ; 27.1)	5.7 ( 1.7 ; 10.1)	6.1 ( 2.0 ; 10.9)	6.7 ( -8.7 ; 27.0)
	Smoking	10.6 ( 8.3 ; 13.1)	7.8 ( 6.1 ; 9.7)	-25.9 (-44.1 ; -2.7)	37.4 ( 32.5 ; 42.7)	28.9 ( 24.6 ; 33.5)	-22.9 (-35.7 ; -7.0)	21.8 ( 19.1 ; 24.5)	16.9 ( 14.5 ; 19.8)	-22.2 (-34.8 ; -6.2)
**Equatorial Guinea**	All risk factors	312.2 (195.9 ; 439.0)	231.1 (163.4 ; 304.0)	-26.0 (-55.7 ; 25.7)	401.8 (299.2 ; 494.7)	211.0 (155.6 ; 269.7)	-47.5 (-61.9 ; -21.2)	354.2 (266.7 ; 450.9)	224.4 (171.1 ; 285.6)	-36.6 (-56.7 ; -10.7)
	Air pollution	110.1 ( 61.0 ; 175.4)	47.7 ( 28.7 ; 70.1)	-56.7 (-77.8 ; -18.4)	155.9 (112.9 ; 206.2)	49.0 ( 30.6 ; 70.4)	-68.6 (-81.7 ; -48.4)	130.9 ( 92.0 ; 181.6)	48.4 ( 30.5 ; 69.4)	-63.0 (-79.1 ; -41.8)
	Alcohol use	0.9 ( -4.5 ; 7.1)	6.4 ( -0.2 ; 15.1)	607.8 (-3342.7 ; 3175.8)	7.6 ( -3.2 ; 19.9)	13.7 ( 5.2 ; 23.5)	80.7 (-1716.7 ; 1479.2)	3.8 ( -3.0 ; 11.4)	9.3 ( 2.6 ; 17.0)	148.5 (-2409.6 ; 2496.0)
	Dietary risks	97.6 ( 54.9 ; 161.4)	65.3 ( 38.5 ; 104.8)	-33.1 (-59.7 ; 15.0)	146.3 (100.8 ; 204.3)	65.3 ( 42.2 ; 98.3)	-55.4 (-69.0 ; -33.0)	119.6 ( 82.7 ; 171.8)	65.7 ( 41.8 ; 101.7)	-45.0 (-62.8 ; -20.8)
	High body mass index	27.2 ( 7.2 ; 59.7)	68.2 ( 40.0 ; 104.7)	150.9 ( 20.1 ; 665.3)	24.2 ( 4.8 ; 59.4)	55.5 ( 31.8 ; 86.2)	128.9 ( 9.4 ; 813.1)	26.1 ( 6.3 ; 58.3)	63.5 ( 38.5 ; 97.1)	143.0 ( 18.3 ; 670.7)
	High fasting plasma glucose	33.9 ( 17.0 ; 60.7)	44.1 ( 25.0 ; 74.9)	30.0 (-29.6 ; 140.1)	74.7 ( 44.3 ; 116.1)	63.3 ( 39.1 ; 97.4)	-15.2 (-44.9 ; 36.4)	51.0 ( 32.5 ; 77.6)	51.8 ( 31.1 ; 80.7)	1.5 (-35.4 ; 50.4)
	High LDL cholesterol	45.1 ( 23.6 ; 74.6)	39.9 ( 21.4 ; 61.7)	-11.6 (-48.6 ; 55.4)	63.2 ( 42.2 ; 89.2)	36.1 ( 22.2 ; 54.1)	-42.9 (-60.6 ; -11.8)	53.8 ( 34.5 ; 78.9)	38.7 ( 22.3 ; 59.0)	-28.0 (-54.0 ; 5.2)
	High systolic blood pressure	232.7 (145.3 ; 329.7)	179.8 (124.1 ; 241.0)	-22.7 (-55.2 ; 33.7)	273.3 (195.5 ; 347.1)	149.3 (105.4 ; 194.7)	-45.4 (-61.3 ; -17.6)	253.5 (187.1 ; 327.0)	168.9 (124.5 ; 218.2)	-33.4 (-54.9 ; -3.9)
	Kidney dysfunction	14.7 ( 8.0 ; 23.8)	16.2 ( 10.0 ; 24.0)	10.2 (-35.3 ; 95.5)	20.0 ( 13.5 ; 27.8)	14.0 ( 9.3 ; 19.5)	-30.3 (-50.9 ; 3.2)	17.2 ( 11.4 ; 24.7)	15.4 ( 10.1 ; 21.8)	-10.3 (-40.9 ; 29.9)
	Low physical activity	8.6 ( 3.0 ; 19.0)	10.5 ( 4.4 ; 20.2)	21.1 (-27.8 ; 119.4)	6.1 ( 1.7 ; 15.4)	5.9 ( 1.9 ; 13.5)	-3.8 (-38.6 ; 63.1)	7.8 ( 2.8 ; 16.9)	8.8 ( 3.5 ; 17.4)	12.0 (-28.4 ; 76.1)
	Non-optimal temperature	7.4 ( -1.1 ; 18.0)	4.0 ( 0.6 ; 9.8)	-45.8 (-78.1 ; 9.3)	9.4 ( -0.2 ; 22.5)	3.5 ( 0.5 ; 8.9)	-62.6 (-95.2 ; -30.8)	8.4 ( -0.8 ; 19.5)	3.9 ( 0.6 ; 9.6)	-54.0 (-78.8 ; -13.0)
	Other environmental risks	19.6 ( 9.4 ; 35.3)	12.8 ( 6.5 ; 21.1)	-35.1 (-61.2 ; 8.7)	35.5 ( 21.3 ; 51.7)	15.2 ( 8.9 ; 22.8)	-57.4 (-69.5 ; -38.5)	26.6 ( 15.7 ; 40.7)	13.7 ( 7.7 ; 21.3)	-48.4 (-64.0 ; -28.0)
	Smoking	10.3 ( 6.1 ; 16.0)	6.2 ( 3.9 ; 9.2)	-40.0 (-65.6 ; 7.2)	66.3 ( 47.2 ; 86.9)	27.3 ( 18.5 ; 38.1)	-58.9 (-72.9 ; -36.6)	35.0 ( 25.9 ; 45.4)	14.9 ( 10.3 ; 20.7)	-57.4 (-71.3 ; -36.8)
**Guinea-Bissau**	All risk factors	284.9 (210.9 ; 356.1)	300.9 (236.2 ; 378.4)	5.6 (-22.2 ; 46.2)	377.0 (298.8 ; 461.9)	341.4 (278.3 ; 408.3)	-9.5 (-30.7 ; 19.7)	329.8 (270.3 ; 392.1)	320.6 (258.7 ; 395.1)	-2.8 (-24.5 ; 26.0)
	Air pollution	116.1 ( 78.7 ; 161.0)	103.8 ( 77.5 ; 134.4)	-10.6 (-35.2 ; 27.9)	161.3 (116.7 ; 216.6)	125.4 ( 98.1 ; 154.8)	-22.3 (-42.2 ; 6.5)	138.2 (103.2 ; 182.1)	114.0 ( 88.8 ; 142.8)	-17.5 (-37.7 ; 11.4)
	Alcohol use	2.6 ( -1.4 ; 7.3)	2.1 ( -1.8 ; 7.3)	-17.6 (-567.9 ; 611.3)	14.2 ( 5.2 ; 24.2)	11.8 ( 3.7 ; 21.9)	-16.7 (-68.9 ; 85.5)	8.2 ( 2.7 ; 14.4)	6.4 ( 1.4 ; 12.7)	-20.9 (-81.3 ; 97.5)
	Dietary risks	107.8 ( 71.4 ; 153.9)	111.0 ( 77.2 ; 162.2)	3.0 (-25.6 ; 45.6)	165.2 (118.0 ; 222.6)	141.1 (103.9 ; 192.1)	-14.6 (-35.6 ; 15.1)	135.1 ( 98.5 ; 186.1)	124.6 ( 90.2 ; 173.8)	-7.8 (-29.8 ; 21.3)
	High body mass index	36.3 ( 14.0 ; 67.4)	58.1 ( 30.4 ; 92.6)	59.7 ( 7.6 ; 191.7)	27.2 ( 7.6 ; 59.8)	41.7 ( 16.3 ; 74.4)	53.5 ( -0.2 ; 216.2)	32.0 ( 11.2 ; 62.9)	51.0 ( 25.4 ; 85.8)	59.3 ( 11.3 ; 181.6)
	High fasting plasma glucose	33.0 ( 19.5 ; 55.8)	68.8 ( 41.2 ; 109.3)	108.5 ( 33.7 ; 235.3)	51.1 ( 31.1 ; 84.5)	81.5 ( 48.9 ; 127.5)	59.5 ( 7.0 ; 145.4)	41.5 ( 26.5 ; 65.9)	74.0 ( 46.3 ; 114.7)	78.4 ( 25.1 ; 152.6)
	High LDL cholesterol	48.5 ( 30.5 ; 74.6)	58.8 ( 38.4 ; 84.2)	21.5 (-16.1 ; 76.4)	68.1 ( 45.1 ; 97.7)	69.3 ( 47.8 ; 94.4)	1.7 (-25.0 ; 41.2)	57.8 ( 39.1 ; 82.7)	63.9 ( 42.8 ; 88.4)	10.4 (-17.9 ; 50.7)
	High systolic blood pressure	194.4 (137.7 ; 255.5)	212.7 (159.7 ; 275.9)	9.4 (-21.2 ; 57.1)	233.4 (176.8 ; 295.8)	225.7 (174.4 ; 280.9)	-3.3 (-28.5 ; 32.7)	214.0 (168.0 ; 264.6)	220.4 (171.5 ; 277.2)	3.0 (-22.5 ; 35.5)
	Kidney dysfunction	19.2 ( 13.0 ; 26.7)	24.2 ( 17.2 ; 33.5)	26.2 ( -8.1 ; 78.2)	24.4 ( 16.6 ; 33.8)	25.9 ( 18.2 ; 35.0)	6.2 (-19.7 ; 42.9)	21.7 ( 15.6 ; 29.0)	25.1 ( 18.3 ; 34.1)	15.5 (-11.5 ; 52.2)
	Low physical activity	6.3 ( 2.3 ; 14.0)	7.5 ( 2.9 ; 16.4)	20.3 (-16.3 ; 73.0)	8.1 ( 2.2 ; 20.4)	7.8 ( 2.3 ; 19.1)	-2.8 (-26.5 ; 35.7)	7.1 ( 2.4 ; 16.7)	7.7 ( 2.8 ; 17.5)	8.4 (-17.2 ; 44.5)
	Non-optimal temperature	8.4 (-23.0 ; 18.2)	9.5 ( 2.5 ; 17.7)	13.1 (-159.3 ; 104.1)	11.5 (-34.1 ; 23.8)	10.8 ( 1.1 ; 18.2)	-5.8 (-159.8 ; 47.8)	9.9 (-27.3 ; 20.4)	10.1 ( 1.7 ; 17.8)	2.4 (-163.6 ; 60.8)
	Other environmental risks	12.5 ( 5.3 ; 21.2)	14.7 ( 7.2 ; 23.9)	17.3 (-14.5 ; 71.4)	23.1 ( 12.9 ; 35.0)	22.4 ( 13.6 ; 32.9)	-2.8 (-27.3 ; 32.9)	17.6 ( 9.4 ; 26.9)	18.1 ( 10.0 ; 27.6)	3.0 (-21.8 ; 37.3)
	Smoking	12.2 ( 8.6 ; 16.4)	10.9 ( 7.9 ; 14.5)	-10.7 (-40.0 ; 29.7)	48.9 ( 36.7 ; 64.1)	32.8 ( 25.4 ; 40.7)	-33.0 (-52.6 ; -5.2)	29.9 ( 23.1 ; 38.5)	20.9 ( 16.2 ; 26.4)	-30.3 (-50.1 ; -4.3)
**Mozambique**	All risk factors	251.3 (203.0 ; 301.4)	247.6 (192.6 ; 324.9)	-1.5 (-26.5 ; 31.6)	288.1 (234.5 ; 345.8)	370.1 (311.7 ; 432.8)	28.4 ( 2.6 ; 61.0)	270.8 (228.0 ; 315.3)	304.8 (246.3 ; 373.7)	12.6 (-11.7 ; 41.3)
	Air pollution	96.2 ( 68.5 ; 136.1)	79.0 ( 57.2 ; 109.2)	-17.9 (-41.6 ; 17.1)	119.3 ( 88.9 ; 165.1)	134.1 (107.1 ; 163.5)	12.4 (-16.9 ; 49.3)	107.7 ( 80.7 ; 146.4)	104.2 ( 80.7 ; 133.0)	-3.3 (-28.7 ; 29.2)
	Alcohol use	-1.0 ( -2.4 ; 0.5)	-0.8 ( -3.5 ; 2.0)	-26.8 (-844.2 ; 623.6)	-0.8 ( -5.3 ; 3.8)	3.4 ( -6.0 ; 13.8)	-507.8 (-3041.8 ; 2468.5)	-0.9 ( -3.3 ; 1.5)	1.2 ( -3.5 ; 6.3)	-226.5 (-3065.6 ; 2361.7)
	Dietary risks	94.9 ( 56.2 ; 147.7)	85.8 ( 48.2 ; 140.9)	-9.6 (-35.2 ; 22.8)	123.1 ( 80.9 ; 180.3)	135.1 ( 91.4 ; 192.8)	9.8 (-18.0 ; 43.2)	108.7 ( 70.0 ; 161.6)	108.4 ( 68.2 ; 163.7)	-0.3 (-23.8 ; 28.3)
	High body mass index	17.8 ( 4.5 ; 40.2)	41.5 ( 20.3 ; 70.2)	132.9 ( 35.3 ; 494.2)	15.5 ( 3.0 ; 38.3)	49.7 ( 21.6 ; 84.9)	219.9 ( 89.5 ; 870.9)	16.9 ( 3.9 ; 39.9)	46.1 ( 21.2 ; 77.5)	172.9 ( 63.8 ; 609.2)
	High fasting plasma glucose	23.7 ( 14.2 ; 41.7)	34.7 ( 18.7 ; 61.9)	46.0 (-20.0 ; 145.4)	43.0 ( 25.6 ; 67.2)	89.3 ( 57.9 ; 132.1)	107.8 ( 45.6 ; 204.7)	32.2 ( 20.1 ; 50.4)	57.0 ( 36.5 ; 87.5)	76.9 ( 25.5 ; 151.2)
	High LDL cholesterol	30.0 ( 19.1 ; 45.8)	35.1 ( 20.1 ; 54.2)	16.9 (-18.9 ; 61.2)	41.1 ( 28.1 ; 61.4)	62.4 ( 43.1 ; 88.1)	52.0 ( 14.1 ; 104.3)	35.4 ( 24.3 ; 51.8)	47.4 ( 31.1 ; 69.0)	34.0 ( 2.8 ; 74.7)
	High systolic blood pressure	180.7 (139.6 ; 229.1)	185.7 (138.5 ; 247.4)	2.8 (-25.6 ; 42.5)	193.1 (149.7 ; 235.2)	264.3 (213.7 ; 319.3)	36.9 ( 5.2 ; 75.0)	188.8 (153.3 ; 228.7)	224.1 (177.4 ; 281.8)	18.7 ( -9.1 ; 51.3)
	Kidney dysfunction	11.8 ( 8.5 ; 16.3)	14.7 ( 10.1 ; 20.8)	24.3 (-10.0 ; 69.5)	14.8 ( 10.8 ; 20.1)	23.6 ( 17.7 ; 31.1)	59.8 ( 23.9 ; 103.7)	13.3 ( 9.8 ; 17.8)	18.8 ( 13.8 ; 25.3)	41.4 ( 8.8 ; 79.9)
	Low physical activity	2.0 ( 0.7 ; 5.1)	2.4 ( 0.8 ; 6.2)	18.0 (-20.2 ; 70.2)	2.1 ( 0.7 ; 5.7)	3.2 ( 1.0 ; 8.7)	52.3 ( 9.0 ; 106.7)	2.1 ( 0.7 ; 5.4)	2.8 ( 1.0 ; 7.3)	33.0 ( -2.5 ; 77.4)
	Non-optimal temperature	8.2 ( 3.9 ; 13.0)	7.6 ( 4.2 ; 12.1)	-7.5 (-34.6 ; 39.0)	9.4 ( 4.7 ; 15.2)	11.1 ( 6.3 ; 17.5)	18.3 (-11.8 ; 70.2)	8.8 ( 4.3 ; 14.1)	9.2 ( 5.2 ; 14.7)	4.6 (-22.7 ; 52.5)
	Other environmental risks	13.7 ( 6.6 ; 23.4)	14.3 ( 7.7 ; 23.9)	5.0 (-21.6 ; 43.2)	32.9 ( 22.7 ; 46.2)	36.4 ( 25.1 ; 49.3)	10.6 (-12.4 ; 38.7)	22.5 ( 14.4 ; 33.7)	23.4 ( 14.8 ; 34.6)	4.0 (-18.1 ; 30.6)
	Smoking	11.4 ( 8.3 ; 15.5)	10.6 ( 7.3 ; 14.8)	-7.7 (-40.2 ; 38.0)	43.8 ( 33.7 ; 55.6)	50.6 ( 39.6 ; 63.5)	15.4 (-13.7 ; 55.1)	26.7 ( 21.3 ; 32.6)	28.2 ( 21.9 ; 35.4)	5.4 (-21.1 ; 41.0)
**Portugal**	All risk factors	255.9 (229.2 ; 276.1)	83.8 ( 71.0 ; 93.8)	-67.3 (-70.3 ; -64.6)	358.4 (336.7 ; 378.3)	124.2 (112.3 ; 133.7)	-65.3 (-67.6 ; -63.2)	299.9 (275.5 ; 319.2)	102.0 ( 89.8 ; 111.5)	-66.0 (-68.3 ; -63.9)
	Air pollution	21.3 ( 7.7 ; 38.2)	3.1 ( 1.8 ; 4.6)	-85.3 (-92.6 ; -64.5)	30.6 ( 10.1 ; 55.6)	5.2 ( 3.0 ; 7.6)	-82.9 (-91.5 ; -56.4)	25.4 ( 8.8 ; 46.0)	4.1 ( 2.4 ; 5.9)	-83.9 (-91.9 ; -60.0)
	Alcohol use	7.4 ( 1.9 ; 13.3)	1.7 ( 0.4 ; 3.3)	-76.7 (-90.7 ; -55.8)	42.1 ( 26.6 ; 57.0)	12.1 ( 7.4 ; 16.9)	-71.3 (-77.0 ; -65.3)	20.7 ( 14.0 ; 27.6)	5.9 ( 3.8 ; 8.2)	-71.4 (-77.9 ; -64.1)
	Dietary risks	81.0 ( 66.4 ; 97.7)	26.4 ( 20.8 ; 33.1)	-67.5 (-70.7 ; -63.7)	123.9 (102.0 ; 150.3)	45.5 ( 36.5 ; 56.7)	-63.3 (-66.5 ; -59.8)	99.7 ( 82.6 ; 120.2)	34.9 ( 28.2 ; 43.5)	-65.0 (-67.7 ; -61.7)
	High body mass index	38.0 ( 20.3 ; 58.4)	15.8 ( 9.3 ; 23.6)	-58.5 (-65.5 ; -45.3)	47.7 ( 21.5 ; 78.0)	22.1 ( 11.5 ; 34.2)	-53.6 (-60.3 ; -38.4)	42.8 ( 21.6 ; 67.6)	18.9 ( 10.6 ; 28.7)	-55.9 (-61.9 ; -42.4)
	High fasting plasma glucose	57.2 ( 34.6 ; 99.7)	26.7 ( 16.3 ; 43.3)	-53.3 (-68.0 ; -34.2)	72.3 ( 47.1 ; 118.4)	40.5 ( 27.1 ; 60.9)	-44.0 (-59.2 ; -25.7)	64.0 ( 40.9 ; 103.3)	32.7 ( 21.3 ; 51.3)	-48.9 (-61.3 ; -33.1)
	High LDL cholesterol	76.8 ( 48.5 ; 118.7)	23.8 ( 14.8 ; 36.1)	-69.0 (-72.4 ; -65.3)	112.6 ( 79.6 ; 160.9)	38.1 ( 27.7 ; 51.6)	-66.2 (-69.6 ; -62.6)	92.5 ( 62.4 ; 137.0)	30.3 ( 20.8 ; 43.1)	-67.2 (-70.3 ; -63.8)
	High systolic blood pressure	152.8 (116.8 ; 187.4)	48.7 ( 37.7 ; 59.6)	-68.2 (-74.7 ; -60.0)	212.5 (177.4 ; 248.0)	73.0 ( 61.9 ; 85.3)	-65.7 (-70.3 ; -60.7)	179.0 (147.4 ; 210.6)	59.8 ( 49.6 ; 70.1)	-66.6 (-71.0 ; -61.2)
	Kidney dysfunction	23.7 ( 16.9 ; 30.3)	8.2 ( 5.7 ; 10.6)	-65.6 (-69.3 ; -61.9)	28.9 ( 22.0 ; 35.8)	10.2 ( 7.7 ; 12.8)	-64.6 (-67.6 ; -61.4)	26.1 ( 19.3 ; 32.8)	9.1 ( 6.7 ; 11.6)	-65.0 (-68.1 ; -61.8)
	Low physical activity	20.0 ( 8.2 ; 36.7)	6.8 ( 2.9 ; 11.9)	-66.2 (-72.1 ; -57.6)	20.0 ( 6.2 ; 41.6)	7.2 ( 2.4 ; 13.7)	-64.2 (-71.1 ; -52.8)	20.4 ( 7.8 ; 38.8)	7.1 ( 2.8 ; 12.7)	-65.4 (-70.9 ; -57.5)
	Non-optimal temperature	29.1 ( 23.8 ; 34.7)	8.9 ( 7.1 ; 10.8)	-69.4 (-72.1 ; -67.3)	38.5 ( 31.6 ; 45.9)	12.3 ( 10.0 ; 14.8)	-68.1 (-70.1 ; -66.1)	33.1 ( 27.2 ; 39.5)	10.4 ( 8.4 ; 12.5)	-68.5 (-70.6 ; -66.6)
	Other environmental risks	10.4 ( 5.0 ; 16.0)	3.4 ( 1.6 ; 5.4)	-67.5 (-72.3 ; -62.0)	23.6 ( 15.4 ; 32.3)	7.4 ( 4.6 ; 10.5)	-68.5 (-72.4 ; -65.1)	15.7 ( 9.3 ; 22.3)	5.0 ( 2.9 ; 7.4)	-68.0 (-71.7 ; -64.3)
	Smoking	26.6 ( 23.2 ; 30.4)	6.0 ( 5.2 ; 6.8)	-77.6 (-80.8 ; -74.1)	87.2 ( 81.5 ; 93.1)	24.0 ( 22.2 ; 25.9)	-72.5 (-74.8 ; -70.2)	52.2 ( 48.6 ; 55.6)	14.0 ( 12.9 ; 15.1)	-73.2 (-75.4 ; -70.8)
**Sao Tome and Principe**	All risk factors	240.9 (206.0 ; 272.8)	272.0 (212.5 ; 326.4)	12.9 ( -8.6 ; 39.1)	224.4 (186.5 ; 263.0)	260.1 (217.3 ; 295.7)	15.9 ( -5.5 ; 41.8)	230.6 (197.7 ; 262.7)	267.1 (219.4 ; 304.8)	15.8 ( -3.4 ; 38.6)
	Air pollution	87.9 ( 72.5 ; 107.3)	73.5 ( 54.9 ; 93.9)	-16.4 (-36.4 ; 10.2)	80.8 ( 64.8 ; 100.3)	72.4 ( 55.4 ; 88.2)	-10.4 (-31.9 ; 16.1)	84.0 ( 69.4 ; 101.7)	73.1 ( 56.3 ; 89.5)	-12.9 (-32.1 ; 11.2)
	Alcohol use	1.3 ( -2.5 ; 5.7)	3.9 ( -1.4 ; 10.3)	214.7 (-2697.6 ; 2301.0)	6.6 ( 1.5 ; 12.5)	11.9 ( 4.9 ; 20.1)	79.8 (-16.3 ; 525.4)	3.7 ( 0.0 ; 8.0)	7.7 ( 2.7 ; 13.8)	106.1 (-92.6 ; 987.4)
	Dietary risks	82.0 ( 61.8 ; 114.3)	91.7 ( 62.9 ; 132.3)	11.8 (-11.2 ; 36.8)	88.5 ( 66.0 ; 119.8)	98.3 ( 71.9 ; 133.7)	11.0 ( -9.5 ; 37.2)	83.4 ( 63.4 ; 114.3)	94.9 ( 68.3 ; 130.9)	13.8 ( -6.5 ; 37.5)
	High body mass index	40.8 ( 21.0 ; 65.1)	64.8 ( 40.1 ; 96.1)	59.1 ( 14.3 ; 142.9)	21.5 ( 7.3 ; 42.0)	50.7 ( 29.9 ; 79.0)	135.6 ( 58.2 ; 376.5)	31.6 ( 14.9 ; 52.7)	58.2 ( 36.2 ; 86.4)	84.2 ( 35.0 ; 190.2)
	High fasting plasma glucose	42.7 ( 25.3 ; 71.2)	73.4 ( 43.0 ; 115.0)	71.8 ( 21.1 ; 152.3)	47.7 ( 25.7 ; 82.7)	78.2 ( 47.0 ; 119.5)	64.0 ( 18.5 ; 146.4)	43.8 ( 25.8 ; 72.4)	75.6 ( 46.5 ; 116.9)	72.8 ( 31.4 ; 133.4)
	High LDL cholesterol	44.1 ( 29.9 ; 61.5)	59.2 ( 37.4 ; 83.0)	34.5 ( 6.5 ; 67.7)	43.7 ( 27.9 ; 63.6)	58.9 ( 39.4 ; 80.6)	34.9 ( 7.2 ; 68.4)	43.3 ( 28.8 ; 61.0)	59.3 ( 39.6 ; 81.4)	36.9 ( 12.1 ; 66.6)
	High systolic blood pressure	166.4 (132.1 ; 200.7)	193.5 (142.8 ; 241.7)	16.3 (-11.9 ; 50.3)	143.7 (111.1 ; 179.0)	176.4 (139.9 ; 210.6)	22.8 ( -2.3 ; 56.2)	154.9 (125.9 ; 183.4)	186.1 (144.7 ; 223.2)	20.1 ( -3.6 ; 48.6)
	Kidney dysfunction	20.0 ( 15.0 ; 25.0)	30.4 ( 22.3 ; 39.7)	52.2 ( 23.1 ; 90.2)	15.5 ( 10.9 ; 20.9)	23.7 ( 17.1 ; 30.6)	53.0 ( 24.6 ; 90.1)	17.8 ( 13.0 ; 22.7)	27.3 ( 20.3 ; 35.1)	53.8 ( 28.1 ; 87.3)
	Low physical activity	6.4 ( 2.6 ; 13.3)	9.0 ( 3.7 ; 18.3)	41.3 ( 8.6 ; 82.0)	5.5 ( 1.6 ; 13.1)	7.1 ( 2.1 ; 16.8)	29.1 ( 0.6 ; 67.7)	5.9 ( 2.2 ; 13.2)	8.2 ( 3.0 ; 17.3)	37.7 ( 12.3 ; 70.2)
	Non-optimal temperature	0.9 ( -3.0 ; 4.6)	1.3 ( -0.7 ; 4.8)	40.9 (-303.6 ; 394.2)	0.9 ( -2.7 ; 4.4)	1.2 ( -0.8 ; 4.7)	43.8 (-316.7 ; 362.9)	0.9 ( -2.8 ; 4.4)	1.2 ( -0.7 ; 4.7)	44.4 (-370.9 ; 366.8)
	Other environmental risks	7.7 ( 2.4 ; 13.3)	8.8 ( 3.1 ; 15.2)	14.6 ( -8.7 ; 52.3)	9.7 ( 4.7 ; 15.9)	11.3 ( 5.8 ; 17.6)	16.7 ( -6.3 ; 46.6)	8.4 ( 3.4 ; 14.0)	10.0 ( 4.4 ; 15.9)	18.3 ( -1.9 ; 47.9)
	Smoking	6.1 ( 4.7 ; 7.8)	7.0 ( 5.1 ; 9.1)	14.8 (-17.7 ; 62.0)	17.7 ( 13.7 ; 22.3)	23.4 ( 18.1 ; 28.6)	32.3 ( -0.5 ; 79.4)	11.5 ( 9.1 ; 14.0)	14.9 ( 11.6 ; 18.2)	29.4 ( 0.1 ; 72.0)
**Timor-Leste**	All risk factors	255.1 (202.6 ; 310.9)	298.2 (246.9 ; 347.9)	16.9 ( -9.0 ; 49.2)	237.9 (182.3 ; 321.3)	346.6 (263.2 ; 447.6)	45.7 ( 9.6 ; 82.9)	247.0 (202.6 ; 304.4)	322.3 (260.4 ; 389.9)	30.5 ( 2.5 ; 60.2)
	Air pollution	98.5 ( 73.5 ; 129.6)	89.6 ( 71.5 ; 108.6)	-9.0 (-33.0 ; 22.3)	88.4 ( 63.6 ; 123.7)	102.6 ( 71.9 ; 141.3)	16.1 (-16.8 ; 51.6)	93.6 ( 71.2 ; 121.9)	96.1 ( 73.8 ; 120.9)	2.6 (-23.0 ; 31.3)
	Alcohol use	-0.2 ( -0.7 ; 0.3)	0.3 ( -0.9 ; 1.9)	-233.5 (-2319.5 ; 2717.1)	0.9 ( -2.5 ; 5.0)	10.2 ( 0.6 ; 21.6)	1094.3 (-8391.8 ; 11248.0)	0.4 ( -1.4 ; 2.5)	5.2 ( 0.1 ; 11.6)	1361.0 (-5698.7 ; 5258.1)
	Dietary risks	110.6 ( 75.5 ; 154.4)	121.1 ( 85.6 ; 164.7)	9.5 (-16.5 ; 42.3)	115.9 ( 78.9 ; 164.4)	158.6 (107.4 ; 222.2)	36.9 ( 2.2 ; 75.5)	113.3 ( 80.3 ; 155.5)	139.7 ( 98.5 ; 190.0)	23.3 ( -5.1 ; 52.1)
	High body mass index	13.1 ( 3.0 ; 30.6)	20.5 ( 6.7 ; 42.0)	56.4 ( 4.4 ; 199.8)	8.7 ( 1.4 ; 23.7)	22.8 ( 7.1 ; 47.2)	163.1 ( 61.7 ; 605.6)	10.9 ( 2.3 ; 26.8)	21.7 ( 7.1 ; 44.2)	99.5 ( 36.3 ; 308.3)
	High fasting plasma glucose	30.1 ( 18.5 ; 49.0)	89.0 ( 57.0 ; 136.3)	196.0 (101.9 ; 326.6)	34.4 ( 18.9 ; 59.9)	103.4 ( 63.7 ; 163.1)	200.7 ( 99.0 ; 358.7)	32.0 ( 19.6 ; 52.7)	96.0 ( 61.3 ; 148.9)	199.7 (117.9 ; 316.8)
	High LDL cholesterol	48.3 ( 32.7 ; 68.4)	61.7 ( 39.9 ; 87.0)	27.9 ( -2.7 ; 65.4)	42.3 ( 27.0 ; 63.8)	65.3 ( 39.4 ; 97.4)	54.4 ( 12.5 ; 98.9)	45.6 ( 30.5 ; 64.0)	63.6 ( 41.2 ; 89.3)	39.6 ( 7.3 ; 75.0)
	High systolic blood pressure	168.4 (127.6 ; 214.7)	190.1 (148.9 ; 236.4)	12.9 (-15.1 ; 51.4)	149.2 (109.9 ; 204.3)	230.8 (169.5 ; 308.2)	54.7 ( 14.2 ; 101.2)	159.4 (125.0 ; 204.5)	210.4 (163.4 ; 263.8)	32.0 ( 1.0 ; 67.3)
	Kidney dysfunction	23.4 ( 16.6 ; 31.2)	35.2 ( 25.8 ; 46.0)	50.7 ( 15.7 ; 95.4)	19.5 ( 12.7 ; 28.7)	35.5 ( 23.4 ; 50.7)	82.2 ( 36.7 ; 134.1)	21.5 ( 15.5 ; 29.4)	35.4 ( 25.2 ; 47.6)	64.5 ( 28.1 ; 103.7)
	Low physical activity	5.7 ( 2.0 ; 13.0)	7.6 ( 2.6 ; 16.6)	32.5 ( -3.5 ; 78.3)	6.2 ( 1.8 ; 13.9)	9.7 ( 3.0 ; 22.2)	56.8 ( 16.5 ; 103.8)	6.0 ( 2.1 ; 13.3)	8.6 ( 2.8 ; 19.5)	44.9 ( 13.0 ; 81.2)
	Non-optimal temperature	4.2 ( 0.7 ; 7.9)	4.3 ( 1.4 ; 8.2)	2.5 (-47.5 ; 103.8)	3.9 ( 0.7 ; 7.5)	4.9 ( 1.6 ; 9.7)	26.9 (-34.7 ; 154.0)	4.0 ( 0.7 ; 7.6)	4.6 ( 1.5 ; 9.0)	14.0 (-40.0 ; 126.9)
	Other environmental risks	8.5 ( 2.6 ; 15.7)	10.9 ( 4.3 ; 18.8)	27.9 ( -1.2 ; 83.5)	12.7 ( 6.4 ; 20.9)	19.2 ( 10.6 ; 30.2)	50.8 ( 11.8 ; 98.9)	10.6 ( 4.6 ; 17.9)	15.0 ( 7.7 ; 23.4)	41.9 ( 9.3 ; 83.4)
	Smoking	25.3 ( 18.6 ; 33.4)	23.5 ( 17.9 ; 30.3)	-7.0 (-32.4 ; 27.8)	69.6 ( 51.3 ; 95.4)	93.3 ( 66.3 ; 126.1)	33.9 ( -5.2 ; 76.5)	47.6 ( 36.7 ; 62.5)	58.2 ( 42.8 ; 76.5)	22.5 (-10.5 ; 59.1)

LDL: low-density lipoprotein cholesterol.

Particularly for the estimates of Brazil, more than 200 data sources were included, from national surveys, such as the National Health Survey (*Pesquisa Nacional de Saúde*: PNS), the surveillance of risk and protection factors for chronic diseases by telephone survey (*Vigilância de fatores de risco e proteção para doenças crônicas por inquérito telefônico*: VIGITEL), the National Household Survey Sample (*Pesquisa Nacional por Amostra de Domicílios*), to the National School-based Health Survey and other cohort studies.^9-16^ Different data sources were used according to the particularities of each PSC.^2,4^

To estimate the burden of disease attributable to RF, the GBD follows the comparative risk assessment (CRA) framework. In brief, CRA is processed through five steps: 1) estimation of the exposure level from available sources, such as household surveys, administrative data, censuses, vital records, and environmental measures. After identifying the data, different definitions are standardized, in addition to adjustments by gender and standardized age groups – a step called *Crosswalking*. Then, spatial-temporal smoothing analyses are performed to estimate data over time, age group and area and, finally, the 95% confidence interval for the estimates (95%CI) are calculated; 2) identification of risk-outcome pairs, according to available evidence; 3) calculation of relative risk (RR), identified in cohort studies, and synthesized by meta-analysis and meta-regression methods. The RRs used by the GBD are universal, the same for morbidity and mortality, and applied to men and women and to all countries and geographic regions; 4) estimation of the *Theoretical Minimum Risk Exposure Level* (TMREL), defined as the minimum exposure level to each FR that would result in the lowest possible probability of a clinical event be attributable to it. The TMREL is used to calculate the population attributable factor (PAF) for different causes of death, diseases or disabilities; 5) calculation of the population attributable fraction, defined as the proportion of the number of cases that can be independently attributed to a given exposure.^4,15^

According to the GBD 2019 study, the estimated TMREL for the RF evaluated in the present study are:1) Systolic blood pressure (SBP): 110 to 115 mmHg; 2) Fasting blood glucose: 85 to 99 mg/dL; 3) LDL cholesterol: between 27 and 50 mg/dL; 4) BMI: 20 to 25 kg/m^2^ for adults; 5) renal function: albumin-to-creatinine ratio <30 mg/g or glomerular filtration rate >60 mL/min per 1.73 m^2^ ; 6) ambient air pollution: 2.4 to 5.9 μg/m3; 7) tobacco: no exposure, including secondhand smoke; 8) dietary habits, including salt intake of 1 - 5 g and 200 to 400 g of fruits and vegetables daily, among others; 9) physical activity: 8,000 METs per day; 10) alcohol use: no consumption; 11) ideal temperature: 25.6ºC. In this study, the 12th group of other environmental risk factors was also considered, which do not include air pollution, ambient temperature and exposure to tobacco smoke.^4^

To estimate exposure to risk factors, the GBD uses the summary measure of risk exposure (summary exposure value, or SEV), which represents the risk-weighted prevalence. The scale for the SEV ranges from 0 to 100%, with 0% reflecting no exposure to risk and 100% indicating maximum exposure. A decline in SEV indicates reduced exposure and an increase in SEV means the opposite. The SEV is estimated for each age, sex, location and year. The detailed methodology for estimating the SEV has been previously published.^4,15,16^

### Definitions of cardiovascular diseases

Standard definitions of the CVDs were used.^2^ Ischemic heart disease include acute myocardial infarction,^17^ stable angina (defined by the Rose Angina Questionnaire), chronic ischemic heart disease and heart failure secondary to myocardial ischemia. For stroke, acute and persistent clinical signs of brain dysfunction that lasted >24 hours or caused death were considered (World Health Organization). Lower extremity peripheral artery disease was defined as an ankle-brachial index <0.9, and for aortic aneurysm, the presence of thoracic and abdominal aneurysms was considered. Atrial fibrillation and *flutter* were diagnosed by electrocardiogram. For hypertensive heart disease, symptomatic heart failure was considered due to the direct and indirect long-term effects attributable to hypertension. Cardiomyopathy was defined as symptomatic heart failure due to primary myocardial disease or exposure to toxins, whereas acute myocarditis was defined as an acute, time-limited condition due to inflammation. For endocarditis and rheumatic heart disease, clinical diagnosis was used, and estimates for rheumatic heart disease included cases identified by clinical history, physical examination, or standardized echocardiographic criteria for definite disease (including subclinical disease). For non-rheumatic valve diseases, calcific aortic valve disease, mitral degenerative valve disease and others were considered.^2,8^

### Statistical analysis

The statistical models reported in the GBD 2019 study were used (Supplement 1: Supplementary Methods).^2,4,7^ Data sources for models are available online at the Global Health Data Exchange website (*http://ghdx.healthdata.org/*).^8^

### Metrics

In the present study, the metrics used to estimate the burden of disease attributable to RF were mortality and years of life lost due to disability – disability-adjusted life years (DALYs) – from 1990 to 2019.

For Brazil, GBD mortality estimates have some particularities. Mortality was estimated using data from the mortality information system (*Sistema de Informação Sobre Mortalidade*: SIM) coded according to the International Classification of Diseases.^18^ To adjust for quality issues in the reporting of causes of death, corrections were made for underreporting of deaths and for causes considered of little use for public health, called *garbage codes*, or unspecific causes. *Garbage code* redistribution algorithms were developed by the GBD study considering evidence from various sources, such as medical literature, expert opinion, and statistical techniques.^7^

To calculate DALYs, the years of life lost due to premature death (years of life lost, YLLs) are added, with reference to the maximum observed life expectancy, to the years lived with disability (YLD). YLDs represent the non-fatal disease burden and are determined by the prevalence of the condition multiplied by the *disability weight* caused by the condition. The prevalence of the conditions was estimated using representative population data, including cohort studies, registries, population surveys, and administrative data, applying statistical methods that adjust for differences in study definitions and methods. *Disability weights* reflect the severity of different conditions and were developed through previously validated surveys with the general population.^8^

In comparisons over time and between PSC, age-standardized rates were considered utilizing the direct method, using the global age composition of the GBD 2019. For the other analyses, non-standardized rates were presented. For each of the analyzed RFs, the attributable burden for total CVDs and for each disease separately, when applicable, was estimated. The ranking of RF was constructed to assess the changes that occurred between 1990 and 2019, according to sex, as well as the risk factor *ranking* for each of the PSC in 2019. The 95% UI was calculated and cited for each estimate, as previously described in the GBD methodology.^2^

### Sociodemographic index

The sociodemographic index (SDI) is used by the GBD as an estimate of the socioeconomic level of each country to assess its association with CVD risk factor and burden metrics, as a function of the global epidemiological transition.^4,7^ SDI was calculated for each country or territory from 1990 to 2019 and represents the weighted geometric mean of *per capita* income, education level and total fertility rate, allowing for the comparison of the performance of each country with others of similar socioeconomic level.

Additionally, the SPSS software version 23.0 for Mac OSX (*SPSS Inc., Chicago, Illinois*) was used to perform correlations (*Spearman’s* method) between the percent change in age-standardized mortality rates and SEV between 1990 and 2019 and the SDI of each PSC in 2019. A p-value <0.05 was considered statistically significant.

## Results

The geographic and sociodemographic characteristics of each of the PSC can be accessed in Table S1.

The percent contribution of CVD attributable to RF to mortality in 2019 in the different PSC was heterogeneous, ranging from 32.1%, 31.7%, 30.7% and 28.2% in Portugal, East Timor, Cape Verde and Brazil, respectively, to rates as low as 12% to 13.9% in the other countries (Figure S1). The percentage attributable to RF was high (>75%) in all PSC, being lowest in Portugal (78.8%) and Brazil (82.6%). Table S2 shows the age-standardized SEV rates for each cardiovascular RF, with 95% UI, by sex, for 1990 and 2019 and the percent change in the period. A significant increase in SEV related to alcohol consumption and high BMI was observed in all countries. For high SBP, a significant reduction was observed in Portugal and a trend towards stability in Brazil and East Timor, contrasting with a trend to increase in other countries, especially Equatorial Guinea (Table S2).

Figure 1 shows the *ranking* of age-standardized CVD mortality rates attributable to RF in the PSC, by sex, in 1990 and 2019. It was observed that high SBP remained as the main risk factor for CVD in all countries during this period. There was an increase in the importance of high fasting blood glucose – except in Brazil – and this factor ranked between the 3^rd^ and 4^th^ positions in 2019 in all PSC. On the other hand, smoking fell in the ranking of attributable mortality in all countries except Sao Tome and Principe (where it remained in the 8^th^ the position). There was a greater reduction in Brazil (3^rd^ to 6^th^) and Equatorial Guinea (6^th^ to 8^th^). High LDL cholesterol had a stable or decreasing trend in all countries, except in Portugal and Brazil. The Figure S2 depicts a similar pattern for DALYs rates attributable to cardiovascular RF.

**Figure 1:** Ranking of cardiovascular disease age-standardized mortality rates (/100,000 inhabitants) attributable to risk factors in Portuguese-speaking countries in 1990 and 2019.

The Figure S3 shows the crude and age-standardized CVD mortality and DALYs rates attributed to the selected FR between 1990 and 2019. There was an increase in the absolute number of CVD deaths and DALYs attributable to all FR except for some trends observed in Portugal, with a decline for dietary factors, high LDL cholesterol and high SBP, and a stable trend for high BMI and high fasting plasma glucose. On the other hand, when analyzing age-standardized mortality rate and DALYs, there is a contrast between Brazil and Portugal – which showed a decline for all RFs – and the other PSC, that showed a trend towards stability or increase. The exception was smoking, which declined in all PSC, except for Mozambique, São Tomé and Príncipe and East Timor (Figure S3, Tables 1 and 2, Supplementary Tables 3 and 4).

**Table 2:** Age-standardized disability-adjusted life years (DALYs) per 100,000 inhabitants

Countries	Risk Factors	Female			Male			Both		
		1990	2019	Percent Change %	1990	2019	Percent Change %	1990	2019	Percent Change %
**Angola**	All risk factors	5999.5 (4395.2 ; 7665.4)	4685.7 (3830.6 ; 5842.6)	-21.9 (-42.3 ; 9.6)	7806.8 (6071.9 ; 9648.6)	5927.2 (4931.1 ; 7175.3)	-24.1 (-42.7 ; 4.1)	6929.8 (5616.7 ; 8339.7)	5274.4 (4415.4 ; 6432.1)	-23.9 (-40.1 ; -0.7)
	Air pollution	2127.2 (1414.2 ; 2901.9)	1115.8 (806.5 ; 1503.7)	-47.5 (-63.4 ; -22.7)	3009.4 (2271.4 ; 3794.0)	1590.1 (1196.9 ; 2069.5)	-47.2 (-62.6 ; -24.9)	2577.7 (1987.6 ; 3287.7)	1336.5 (992.5 ; 1759.4)	-48.2 (-62.4 ; -28.8)
	Alcohol use	15.3 (-77.1 ; 114.3)	198.8 ( 70.0 ; 347.4)	1202.9 (-11035.7 ; 5853.2)	134.6 (-44.1 ; 339.4)	503.4 (303.1 ; 728.6)	273.9 (-2894.5 ; 3150.6)	74.5 (-35.3 ; 193.4)	335.1 (209.2 ; 493.8)	349.7 (-3664.4 ; 3172.1)
	Dietary risks	2014.8 (1306.8 ; 2966.1)	1425.8 (965.2 ; 2141.5)	-29.2 (-49.7 ; 2.1)	3077.2 (2228.5 ; 4197.0)	2070.4 (1501.1 ; 2877.1)	-32.7 (-50.8 ; -4.7)	2556.4 (1894.9 ; 3468.2)	1726.4 (1214.6 ; 2489.6)	-32.5 (-49.2 ; -8.7)
	High body mass index	509.4 (110.9 ; 1175.3)	1039.4 (534.9 ; 1624.8)	104.0 ( 12.2 ; 528.9)	551.6 (108.5 ; 1342.1)	1064.7 (505.3 ; 1729.3)	93.0 ( 8.1 ; 500.2)	532.7 (113.3 ; 1263.7)	1055.2 (545.3 ; 1649.2)	98.1 ( 13.9 ; 486.0)
	High fasting plasma glucose	616.7 (366.8 ; 994.2)	732.7 (425.4 ; 1157.0)	18.8 (-23.8 ; 92.8)	1328.9 (862.0 ; 2000.9)	1562.0 (1032.1 ; 2231.3)	17.5 (-21.1 ; 83.9)	963.5 (652.6 ; 1428.2)	1101.7 (739.8 ; 1595.8)	14.3 (-18.0 ; 66.0)
	High LDL cholesterol	863.5 (551.8 ; 1271.1)	791.9 (523.5 ; 1110.4)	-8.3 (-37.2 ; 33.9)	1329.1 (921.5 ; 1815.8)	1108.4 (789.7 ; 1522.1)	-16.6 (-41.3 ; 20.7)	1105.2 (792.6 ; 1493.0)	943.7 (663.9 ; 1292.7)	-14.6 (-37.7 ; 17.8)
	High systolic blood pressure	4547.3 (3319.4 ; 5912.5)	3629.9 (2857.3 ; 4543.0)	-20.2 (-41.9 ; 14.2)	5449.8 (4097.4 ; 6949.2)	4190.3 (3338.0 ; 5147.4)	-23.1 (-43.4 ; 8.2)	5025.9 (3962.3 ; 6245.9)	3912.6 (3189.4 ; 4828.3)	-22.2 (-40.0 ; 2.6)
	Kidney dysfunction	290.8 (188.4 ; 422.6)	284.8 (203.9 ; 389.4)	-2.1 (-28.8 ; 39.4)	399.0 (275.5 ; 537.0)	364.0 (270.1 ; 491.8)	-8.8 (-32.1 ; 27.1)	346.4 (248.9 ; 471.7)	322.6 (236.2 ; 437.1)	-6.9 (-28.4 ; 22.6)
	Low physical activity	131.1 ( 49.5 ; 276.6)	136.6 ( 55.2 ; 283.5)	4.2 (-26.1 ; 48.7)	99.7 ( 26.1 ; 252.5)	100.8 ( 29.7 ; 250.9)	1.1 (-26.5 ; 47.9)	117.8 ( 40.1 ; 266.5)	123.0 ( 46.0 ; 267.5)	4.4 (-23.3 ; 41.0)
	Non-optimal temperature	215.6 (108.2 ; 345.1)	153.6 ( 90.7 ; 237.8)	-28.7 (-50.8 ; 17.6)	281.9 (149.3 ; 452.1)	192.5 (112.2 ; 302.1)	-31.7 (-51.8 ; 2.2)	249.8 (134.5 ; 392.5)	172.2 (102.1 ; 268.5)	-31.1 (-49.4 ; 2.4)
	Other environmental risks	267.9 (100.7 ; 489.2)	209.8 ( 90.4 ; 363.2)	-21.7 (-42.7 ; 17.1)	485.8 (257.2 ; 760.0)	354.1 (196.6 ; 537.9)	-27.1 (-46.1 ; 1.6)	377.5 (184.9 ; 618.3)	275.0 (141.5 ; 437.0)	-27.1 (-43.4 ; -3.3)
	Smoking	346.2 (233.2 ; 481.0)	272.9 (195.7 ; 369.1)	-21.2 (-47.4 ; 20.3)	1602.4 (1208.8 ; 2044.9)	1163.6 (921.0 ; 1489.6)	-27.4 (-47.9 ; 4.4)	977.9 (757.7 ; 1229.1)	678.9 (539.0 ; 876.8)	-30.6 (-49.5 ; -2.7)
**Brazil**	All risk factors	5140.9 (4871.7 ; 5380.8)	2380.3 (2200.5 ; 2544.9)	-53.7 (-55.9 ; -51.4)	7756.5 (7453.1 ; 8034.5)	3888.3 (3646.9 ; 4112.3)	-49.9 (-52.8 ; -47.3)	6385.9 (6112.9 ; 6619.3)	3075.2 (2881.5 ; 3230.7)	-51.8 (-53.9 ; -50.0)
	Air pollution	1074.6 (805.7 ; 1372.7)	261.8 (189.5 ; 335.6)	-75.6 (-82.6 ; -66.5)	1617.5 (1168.8 ; 2094.1)	421.0 (304.6 ; 550.7)	-74.0 (-81.6 ; -63.1)	1333.9 (972.1 ; 1717.8)	335.8 (245.2 ; 436.8)	-74.8 (-82.3 ; -65.2)
	Alcohol use	24.8 (-16.1 ; 70.3)	14.8 ( -6.7 ; 38.8)	-40.2 (-202.7 ; 184.0)	369.0 (227.5 ; 534.8)	183.5 (103.5 ; 267.1)	-50.3 (-62.5 ; -35.6)	189.0 (114.0 ; 272.5)	92.6 ( 53.3 ; 134.8)	-51.0 (-64.3 ; -34.6)
	Dietary risks	1976.8 (1603.5 ; 2462.7)	830.8 (654.2 ; 1067.4)	-58.0 (-62.0 ; -54.2)	3373.7 (2700.5 ; 4120.9)	1546.0 (1214.4 ; 1948.9)	-54.2 (-58.3 ; -50.0)	2641.3 (2140.9 ; 3252.7)	1160.8 (913.6 ; 1466.2)	-56.1 (-59.5 ; -52.8)
	High body mass index	1462.1 (907.2 ; 2094.3)	924.3 (682.4 ; 1181.6)	-36.8 (-45.0 ; -22.2)	1768.1 (955.9 ; 2704.4)	1315.5 (882.4 ; 1774.0)	-25.6 (-37.9 ; 2.2)	1611.6 (936.5 ; 2376.0)	1108.9 (778.3 ; 1460.7)	-31.2 (-40.5 ; -12.4)
	High fasting plasma glucose	1007.6 (715.9 ; 1432.7)	483.6 (345.7 ; 673.9)	-52.0 (-58.5 ; -44.5)	1593.2 (1141.6 ; 2253.9)	906.1 (635.8 ; 1274.9)	-43.1 (-49.4 ; -35.1)	1279.7 (922.2 ; 1800.5)	673.5 (485.1 ; 947.7)	-47.4 (-52.2 ; -41.9)
	High LDL cholesterol	1425.8 (1165.2 ; 1745.1)	692.2 (567.0 ; 842.5)	-51.5 (-54.8 ; -47.9)	2496.0 (2090.2 ; 2980.2)	1310.6 (1097.4 ; 1543.0)	-47.5 (-50.9 ; -43.8)	1940.1 (1614.4 ; 2322.9)	981.3 (817.1 ; 1162.4)	-49.4 (-52.0 ; -46.8)
	High systolic blood pressure	3264.0 (2911.5 ; 3596.7)	1551.6 (1365.7 ; 1731.8)	-52.5 (-55.6 ; -49.0)	4825.1 (4330.2 ; 5295.9)	2560.7 (2294.0 ; 2810.7)	-46.9 (-50.3 ; -43.5)	4011.3 (3600.8 ; 4407.4)	2019.3 (1806.5 ; 2216.0)	-49.7 (-52.0 ; -47.1)
	Kidney dysfunction	405.4 (330.0 ; 485.6)	204.4 (166.3 ; 247.4)	-49.6 (-53.4 ; -46.4)	625.9 (507.2 ; 754.1)	351.7 (284.7 ; 423.0)	-43.8 (-47.6 ; -39.9)	510.8 (414.8 ; 611.6)	272.1 (221.5 ; 325.5)	-46.7 (-49.5 ; -43.9)
	Low physical activity	387.4 (187.6 ; 624.2)	197.2 (110.7 ; 303.3)	-49.1 (-54.9 ; -38.3)	483.4 (191.0 ; 878.6)	275.7 (127.9 ; 478.4)	-43.0 (-49.8 ; -27.6)	434.5 (193.2 ; 747.3)	233.1 (118.2 ; 375.7)	-46.4 (-52.1 ; -34.5)
	Non-optimal temperature	160.5 ( 44.4 ; 251.2)	55.1 ( 15.0 ; 84.8)	-65.7 (-81.5 ; -34.3)	233.7 ( 53.3 ; 364.4)	87.6 ( 15.8 ; 135.2)	-62.5 (-86.1 ; -17.9)	195.7 ( 47.7 ; 304.9)	70.1 ( 13.4 ; 108.1)	-64.2 (-84.6 ; -30.1)
	Other environmental risks	180.0 ( 62.9 ; 301.9)	68.7 ( 23.0 ; 123.4)	-61.9 (-66.9 ; -58.2)	387.6 (201.3 ; 572.2)	150.1 ( 70.8 ; 237.3)	-61.3 (-67.0 ; -57.0)	277.4 (127.9 ; 428.4)	105.1 ( 44.6 ; 175.8)	-62.1 (-67.2 ; -58.3)
	Smoking	1720.6 (1562.0 ; 1894.4)	524.3 (473.0 ; 579.1)	-69.5 (-72.8 ; -66.1)	3008.3 (2835.8 ; 3176.8)	972.3 (899.1 ; 1048.2)	-67.7 (-70.1 ; -65.1)	2332.2 (2200.7 ; 2463.5)	731.3 (681.2 ; 782.2)	-68.6 (-70.9 ; -66.4)
**Cabo Verde**	All risk factors	3232.1 (2881.9 ; 3588.7)	3229.7 (2736.6 ; 3742.1)	-0.1 (-16.2 ; 19.6)	4980.5 (4462.4 ; 5466.7)	5375.2 (4739.6 ; 6059.0)	7.9 ( -6.4 ; 24.7)	3949.1 (3575.7 ; 4282.9)	4178.4 (3663.4 ; 4722.8)	5.8 ( -8.7 ; 22.2)
	Air pollution	1085.5 (916.9 ; 1284.9)	873.8 (686.1 ; 1074.6)	-19.5 (-37.3 ; 3.1)	1872.6 (1601.9 ; 2162.1)	1641.8 (1300.4 ; 2001.5)	-12.3 (-30.3 ; 7.9)	1409.1 (1221.4 ; 1628.4)	1218.3 (971.7 ; 1472.0)	-13.5 (-31.5 ; 6.8)
	Alcohol use	56.4 ( 1.2 ; 123.5)	53.8 ( -5.9 ; 122.8)	-4.5 (-146.8 ; 342.6)	209.7 ( 95.7 ; 329.3)	296.6 (160.6 ; 461.8)	41.5 (-14.5 ; 145.4)	120.1 ( 54.2 ; 195.2)	161.2 ( 78.9 ; 260.9)	34.2 (-30.0 ; 158.2)
	Dietary risks	1200.0 (929.4 ; 1610.7)	1100.3 (800.4 ; 1528.9)	-8.3 (-25.5 ; 10.9)	2226.2 (1763.9 ; 2828.4)	1984.2 (1477.5 ; 2687.2)	-10.9 (-26.2 ; 6.6)	1620.7 (1283.0 ; 2085.9)	1494.1 (1100.1 ; 2041.3)	-7.8 (-23.1 ; 8.9)
	High body-mass index	667.6 (350.3 ; 1006.8)	996.0 (686.6 ; 1370.1)	49.2 ( 10.9 ; 132.8)	634.4 (231.2 ; 1133.9)	1415.4 (895.0 ; 2019.2)	123.1 ( 55.0 ; 364.7)	651.1 (305.9 ; 1050.2)	1197.0 (801.8 ; 1682.0)	83.8 ( 35.4 ; 201.0)
	High fasting plasma glucose	424.0 (272.3 ; 638.9)	891.1 (569.5 ; 1292.8)	110.2 ( 53.8 ; 192.3)	709.3 (474.4 ; 1049.6)	1390.1 (950.8 ; 1954.4)	96.0 ( 47.5 ; 165.4)	540.6 (356.6 ; 799.3)	1100.1 (744.7 ; 1552.5)	103.5 ( 58.5 ; 165.0)
	High LDL cholesterol	641.5 (478.6 ; 832.2)	754.7 (542.3 ; 998.2)	17.6 ( -4.5 ; 41.4)	1211.2 (921.3 ; 1525.0)	1227.4 (907.3 ; 1582.9)	1.3 (-15.5 ; 20.9)	872.9 (669.5 ; 1097.6)	971.8 (717.6 ; 1245.1)	11.3 ( -4.9 ; 31.5)
	High systolic blood pressure	2304.0 (1921.5 ; 2702.3)	2322.6 (1862.6 ; 2791.3)	0.8 (-20.1 ; 25.0)	3430.1 (2927.3 ; 3948.6)	3862.3 (3257.9 ; 4532.1)	12.6 ( -5.7 ; 34.3)	2764.5 (2413.2 ; 3140.2)	3014.4 (2510.3 ; 3542.8)	9.0 ( -7.2 ; 28.5)
	Kidney dysfunction	204.5 (150.6 ; 268.1)	286.8 (208.8 ; 371.1)	40.2 ( 15.4 ; 72.9)	322.2 (228.8 ; 420.9)	445.8 (330.5 ; 564.7)	38.4 ( 16.5 ; 63.7)	252.7 (185.4 ; 328.1)	356.8 (267.5 ; 455.0)	41.2 ( 21.0 ; 67.0)
	Low physical activity	66.9 ( 25.1 ; 147.8)	90.3 ( 35.4 ; 195.0)	35.0 ( 9.3 ; 70.9)	85.2 ( 23.4 ; 234.3)	107.9 ( 30.4 ; 270.0)	26.6 ( 1.0 ; 64.6)	74.3 ( 23.7 ; 184.2)	98.6 ( 34.5 ; 227.6)	32.6 ( 10.5 ; 63.0)
	Non-optimal temperature	130.6 ( -1.5 ; 271.7)	119.7 ( 31.3 ; 249.1)	-8.4 (-44.8 ; 44.2)	209.7 ( 14.1 ; 434.8)	197.0 ( 43.0 ; 415.5)	-6.1 (-40.8 ; 38.0)	163.1 ( 11.3 ; 338.4)	153.8 ( 37.2 ; 320.2)	-5.7 (-37.9 ; 45.2)
	Other environmental risks	89.7 ( 16.5 ; 174.7)	75.0 ( 15.3 ; 145.3)	-16.4 (-33.1 ; 9.6)	162.8 ( 49.3 ; 289.0)	142.2 ( 40.7 ; 261.4)	-12.6 (-30.3 ; 3.3)	119.8 ( 29.9 ; 216.5)	103.6 ( 26.8 ; 192.6)	-13.5 (-29.3 ; 0.0)
	Smoking	255.0 (200.8 ; 309.6)	177.5 (140.3 ; 219.7)	-30.4 (-47.4 ; -8.8)	1025.1 (887.5 ; 1168.5)	730.9 (610.8 ; 861.7)	-28.7 (-41.4 ; -13.2)	573.3 (506.5 ; 645.6)	427.3 (360.6 ; 500.8)	-25.5 (-38.1 ; -8.9)
**Equatorial Guinea**	All risk factors	6569.8 (4287.1 ; 9089.7)	4163.8 (2926.5 ; 5799.9)	-36.6 (-60.5 ; 4.9)	9527.0 (7147.6 ; 11927.0)	4260.3 (3067.7 ; 5613.6)	-55.3 (-68.4 ; -33.0)	7918.4 (5983.2 ; 9983.4)	4227.0 (3165.8 ; 5693.3)	-46.6 (-62.5 ; -23.9)
	Air pollution	2482.8 (1481.8 ; 3765.3)	982.2 (596.0 ; 1473.7)	-60.4 (-79.0 ; -27.6)	3909.8 (2833.2 ; 5128.4)	1101.5 (689.6 ; 1597.4)	-71.8 (-83.0 ; -54.0)	3128.9 (2266.2 ; 4171.6)	1036.8 (645.1 ; 1528.6)	-66.9 (-80.4 ; -48.9)
	Alcohol use	40.9 (-78.3 ; 193.9)	141.0 ( 13.8 ; 305.7)	244.4 (-3080.2 ; 2575.4)	216.4 (-57.0 ; 539.2)	307.6 (129.9 ; 516.4)	42.1 (-1112.9 ; 1274.7)	118.2 (-46.4 ; 314.2)	210.5 ( 79.5 ; 381.6)	78.1 (-1552.1 ; 1339.9)
	Dietary risks	2125.4 (1242.0 ; 3354.3)	1184.7 (689.2 ; 1888.4)	-44.3 (-67.0 ; -7.2)	3671.8 (2554.0 ; 5092.8)	1360.5 (874.5 ; 2048.1)	-62.9 (-74.7 ; -43.6)	2823.4 (1987.6 ; 3980.8)	1266.3 (821.3 ; 1949.9)	-55.2 (-69.8 ; -34.4)
	High body-mass index	732.6 (198.2 ; 1561.9)	1507.2 (908.0 ; 2310.4)	105.7 ( -0.1 ; 524.7)	705.4 (137.8 ; 1707.1)	1398.5 (815.0 ; 2158.8)	98.3 ( -8.0 ; 714.5)	723.5 (187.0 ; 1594.7)	1471.1 (904.3 ; 2214.8)	103.3 ( -1.7 ; 549.0)
	High fasting plasma glucose	658.2 (357.3 ; 1088.6)	744.7 (434.3 ; 1193.3)	13.1 (-36.4 ; 99.5)	1553.6 (973.5 ; 2331.5)	1175.5 (754.0 ; 1744.0)	-24.3 (-50.4 ; 20.2)	1046.1 (698.0 ; 1551.0)	921.0 (596.3 ; 1372.7)	-12.0 (-42.2 ; 31.2)
	High LDL cholesterol	972.1 (552.2 ; 1559.5)	722.0 (428.7 ; 1124.7)	-25.7 (-57.0 ; 26.1)	1683.6 (1153.8 ; 2312.9)	782.6 (492.7 ; 1176.6)	-53.5 (-68.8 ; -26.2)	1298.4 (875.7 ; 1817.4)	754.8 (478.0 ; 1133.3)	-41.9 (-61.9 ; -13.4)
	High systolic blood pressure	4934.3 (3200.0 ; 6958.9)	3270.3 (2258.5 ; 4577.1)	-33.7 (-59.8 ; 11.2)	6611.8 (4760.9 ; 8476.4)	3093.9 (2140.7 ; 4141.8)	-53.2 (-67.7 ; -29.1)	5714.8 (4252.3 ; 7288.3)	3218.6 (2341.0 ; 4384.1)	-43.7 (-61.2 ; -18.4)
	Kidney dysfunction	321.0 (187.7 ; 508.2)	286.4 (183.8 ; 427.6)	-10.8 (-46.3 ; 51.5)	493.2 (334.7 ; 677.9)	281.3 (190.3 ; 403.4)	-43.0 (-59.7 ; -15.8)	399.3 (278.4 ; 561.6)	286.1 (190.9 ; 411.9)	-28.4 (-51.0 ; 2.9)
	Low physical activity	143.2 ( 49.4 ; 319.9)	152.2 ( 62.1 ; 308.1)	6.2 (-36.3 ; 82.9)	122.1 ( 32.9 ; 318.6)	97.6 ( 28.6 ; 233.5)	-20.1 (-50.0 ; 33.6)	136.9 ( 46.4 ; 310.4)	131.4 ( 50.6 ; 274.1)	-4.0 (-37.4 ; 50.0)
	Non-optimal temperature	145.8 (-15.2 ; 347.3)	65.4 ( 9.8 ; 163.8)	-55.1 (-82.2 ; -10.3)	215.1 ( -4.8 ; 506.9)	65.3 ( 8.9 ; 169.8)	-69.7 (-95.6 ; -42.1)	177.5 (-11.7 ; 418.0)	65.9 ( 9.9 ; 164.6)	-62.9 (-85.6 ; -30.0)
	Other environmental risks	435.3 (213.2 ; 748.2)	212.3 (102.9 ; 355.0)	-51.2 (-70.5 ; -21.8)	879.4 (538.8 ; 1293.4)	280.7 (154.4 ; 437.1)	-68.1 (-78.2 ; -53.3)	633.6 (374.6 ; 952.0)	240.9 (126.7 ; 382.1)	-62.0 (-73.4 ; -46.5)
	Smoking	263.5 (160.7 ; 408.5)	138.5 ( 84.6 ; 219.1)	-47.4 (-70.5 ; -5.7)	1885.9 (1341.1 ; 2486.9)	702.2 (473.2 ; 1003.9)	-62.8 (-75.6 ; -41.8)	990.3 (726.1 ; 1299.4)	377.1 (258.1 ; 542.7)	-61.9 (-74.9 ; -42.9)
**Guinea-Bissau**	All risk factors	6,230.3 (4699.3 ; 7828.9)	6284.9 (4934.5 ; 7962.6)	0.9 (-26.3 ; 39.6)	8677.3 (6803.9 ; 10844.6)	7626.5 (6117.8 ; 9356.4)	-12.1 (-34.9 ; 19.9)	7415.5 (5951.3 ; 9059.8)	6919.5 (5515.9 ; 8586.3)	-6.7 (-29.8 ; 23.5)
	Air pollution	2680.9 (1920.2 ; 3605.5)	2375.9 (1785.9 ; 3066.2)	-11.4 (-36.9 ; 24.9)	3918.3 (2878.4 ; 5226.8)	3047.4 (2349.2 ; 3813.7)	-22.2 (-43.8 ; 9.1)	3279.7 (2470.8 ; 4272.8)	2692.3 (2078.8 ; 3414.8)	-17.9 (-39.5 ; 11.6)
	Alcohol use	90.2 (-11.3 ; 210.3)	76.1 (-23.7 ; 209.3)	-15.6 (-242.3 ; 303.3)	431.4 (195.9 ; 707.7)	350.1 (140.1 ; 607.3)	-18.8 (-62.9 ; 60.8)	254.1 (109.4 ; 424.2)	202.5 ( 68.1 ; 373.0)	-20.3 (-69.4 ; 68.7)
	Dietary risks	2447.2 (1684.4 ; 3503.7)	2406.1 (1665.2 ; 3521.1)	-1.7 (-30.0 ; 38.5)	3923.7 (2770.2 ; 5444.2)	3266.2 (2369.6 ; 4473.7)	-16.8 (-40.0 ; 15.6)	3155.9 (2275.0 ; 4403.3)	2804.6 (1995.1 ; 3895.0)	-11.1 (-34.2 ; 20.6)
	High body mass index	1065.0 (416.6 ; 1923.0)	1665.5 (942.3 ; 2572.9)	56.4 ( 2.1 ; 186.9)	824.4 (233.4 ; 1772.1)	1257.4 (510.7 ; 2209.9)	52.5 ( -1.7 ; 212.7)	950.3 (330.9 ; 1810.6)	1480.4 (763.7 ; 2401.0)	55.8 ( 6.2 ; 186.6)
	High fasting plasma glucose	609.5 (387.3 ; 951.1)	1235.5 (745.1 ; 1946.8)	102.7 ( 31.1 ; 221.6)	969.6 (628.3 ; 1475.4)	1460.9 (933.3 ; 2188.9)	50.7 ( 0.5 ; 132.0)	782.3 (518.7 ; 1164.3)	1332.4 (863.9 ; 2007.8)	70.3 ( 18.8 ; 144.0)
	High LDL cholesterol	1079.7 (717.8 ; 1574.2)	1276.2 (872.9 ; 1764.5)	18.2 (-20.2 ; 72.0)	1665.4 (1122.7 ; 2384.7)	1657.0 (1154.2 ; 2188.0)	-0.5 (-30.4 ; 41.9)	1358.3 (940.5 ; 1914.3)	1456.2 (1018.8 ; 1943.0)	7.2 (-23.2 ; 51.4)
	High systolic blood pressure	4332.4 (3146.7 ; 5614.9)	4529.6 (3409.1 ; 5890.7)	4.6 (-25.3 ; 47.8)	5534.3 (4184.5 ; 7156.4)	5216.6 (3984.4 ; 6616.8)	-5.7 (-32.5 ; 31.7)	4916.5 (3848.8 ; 6174.5)	4866.1 (3790.0 ; 6146.4)	-1.0 (-26.6 ; 32.7)
	Kidney dysfunction	414.3 (293.9 ; 562.3)	490.9 (352.1 ; 667.9)	18.5 (-15.8 ; 65.4)	549.5 (381.0 ; 759.5)	558.5 (400.8 ; 742.1)	1.6 (-25.7 ; 37.9)	479.7 (349.3 ; 634.7)	523.4 (379.7 ; 701.7)	9.1 (-18.4 ; 46.1)
	Low physical activity	102.1 ( 35.2 ; 236.2)	118.9 ( 42.5 ; 276.8)	16.5 (-17.9 ; 66.6)	145.9 ( 39.6 ; 377.6)	137.4 ( 40.0 ; 350.7)	-5.8 (-31.5 ; 32.2)	123.4 ( 38.7 ; 302.8)	128.1 ( 42.0 ; 307.6)	3.9 (-20.9 ; 40.1)
	Non-optimal temperature	174.0 (-486.0 ; 390.6)	185.3 ( 28.6 ; 332.2)	6.5 (-163.7 ; 90.3)	253.7 (-731.3 ; 532.3)	227.8 ( 12.2 ; 389.2)	-10.2 (-157.2 ; 40.9)	212.6 (-593.7 ; 433.0)	205.4 ( 35.6 ; 361.4)	-3.4 (-168.6 ; 52.4)
	Other environmental risks	286.6 (115.1 ; 489.2)	292.8 (126.2 ; 493.2)	2.2 (-26.4 ; 49.3)	559.3 (303.4 ; 866.5)	484.1 (277.2 ; 732.4)	-13.4 (-37.2 ; 21.5)	417.5 (208.7 ; 652.4)	379.9 (197.8 ; 600.9)	-9.0 (-33.8 ; 22.7)
	Smoking	328.4 (227.6 ; 452.0)	287.5 (204.4 ; 385.8)	-12.4 (-42.4 ; 28.9)	1388.6 (1014.8 ; 1840.9)	918.1 (700.6 ; 1162.9)	-33.9 (-54.5 ; -4.6)	837.9 (637.7 ; 1089.7)	580.5 (446.5 ; 734.0)	-30.7 (-51.5 ; -3.9)
**Mozambique**	All risk factors	4984.7 (4089.4 ; 5939.3)	4771.3 (3678.2 ; 6235.3)	-4.3 (-29.1 ; 28.7)	6407.0 (5226.9 ; 7754.2)	8455.5 (6898.2 ; 10077.4)	32.0 ( 3.3 ; 67.2)	5688.1 (4798.3 ; 6665.6)	6479.1 (5166.2 ; 8024.3)	13.9 (-11.7 ; 44.9)
	Air pollution	2035.6 (1527.2 ; 2728.0)	1691.8 (1227.1 ; 2338.9)	-16.9 (-40.8 ; 16.5)	2776.1 (2089.8 ; 3714.1)	3312.6 (2608.0 ; 4064.1)	19.3 (-11.4 ; 59.6)	2397.5 (1852.1 ; 3150.0)	2440.8 (1876.1 ; 3110.1)	1.8 (-24.4 ; 36.3)
	Alcohol use	-20.9 (-52.7 ; 14.1)	-10.9 (-69.6 ; 54.9)	-47.6 (-935.3 ; 689.0)	-7.5 (-117.8 ; 110.2)	118.4 (-120.0 ; 385.4)	-1688.0 (-4458.0 ; 4236.8)	-14.2 (-73.8 ; 48.0)	49.0 (-69.1 ; 186.7)	-444.1 (-2464.3 ; 3711.1)
	Dietary risks	1965.6 (1178.6 ; 3032.0)	1714.4 (977.2 ; 2743.3)	-12.8 (-37.5 ; 20.2)	2806.2 (1846.8 ; 4085.1)	3164.3 (2127.9 ; 4520.4)	12.8 (-16.0 ; 51.4)	2373.1 (1546.7 ; 3505.3)	2383.0 (1518.4 ; 3527.0)	0.4 (-23.6 ; 32.4)
	High body mass index	470.9 (131.6 ; 997.6)	1098.0 (579.1 ; 1761.4)	133.2 ( 32.4 ; 484.0)	437.0 ( 89.7 ; 1040.9)	1477.1 (679.9 ; 2453.0)	238.0 ( 98.9 ; 908.9)	457.0 (115.5 ; 1038.7)	1283.8 (634.4 ; 2077.3)	180.9 ( 67.8 ; 605.3)
	High fasting plasma glucose	415.2 (266.7 ; 674.7)	611.2 (351.5 ; 1029.6)	47.2 (-14.4 ; 136.1)	755.9 (475.7 ; 1128.2)	1745.4 (1139.4 ; 2522.4)	130.9 ( 61.8 ; 241.8)	571.2 (381.3 ; 824.4)	1101.3 (720.8 ; 1622.2)	92.8 ( 37.9 ; 173.5)
	High LDL cholesterol	616.5 (432.7 ; 878.1)	694.6 (447.4 ; 1017.6)	12.7 (-20.9 ; 56.5)	949.1 (676.3 ; 1329.1)	1496.6 (1065.4 ; 2013.3)	57.7 ( 17.1 ; 116.3)	777.2 (566.7 ; 1090.9)	1062.9 (745.3 ; 1448.8)	36.7 ( 2.9 ; 82.7)
	High systolic blood pressure	3718.3 (2937.6 ; 4645.2)	3668.6 (2693.3 ; 4911.8)	-1.3 (-28.8 ; 37.3)	4566.7 (3562.9 ; 5600.0)	6288.4 (5000.2 ; 7703.7)	37.7 ( 6.4 ; 78.1)	4147.7 (3396.3 ; 5038.5)	4901.3 (3813.0 ; 6156.2)	18.2 ( -9.7 ; 52.3)
	Kidney dysfunction	246.8 (184.3 ; 333.0)	290.4 (200.8 ; 408.5)	17.7 (-14.5 ; 61.2)	339.5 (250.3 ; 455.6)	547.5 (407.3 ; 713.9)	61.3 ( 23.7 ; 108.8)	292.0 (221.2 ; 384.1)	408.8 (302.2 ; 543.5)	40.0 ( 7.2 ; 78.8)
	Low physical activity	31.2 ( 10.9 ; 86.8)	35.6 ( 12.3 ; 95.7)	14.0 (-19.7 ; 59.8)	38.3 ( 11.9 ; 109.6)	59.7 ( 18.2 ; 168.5)	55.9 ( 13.5 ; 107.4)	34.9 ( 11.8 ; 96.3)	46.8 ( 15.5 ; 130.9)	34.0 ( 0.9 ; 75.9)
	Non-optimal temperature	149.8 ( 70.6 ; 247.3)	132.2 ( 71.2 ; 216.2)	-11.8 (-39.2 ; 35.3)	194.7 ( 95.0 ; 318.5)	237.2 (131.7 ; 383.7)	21.9 (-10.6 ; 77.9)	171.9 ( 84.7 ; 278.4)	180.8 ( 98.6 ; 290.4)	5.2 (-23.0 ; 56.2)
	Other environmental risks	261.7 (121.8 ; 446.7)	250.6 (125.9 ; 417.4)	-4.2 (-29.7 ; 32.6)	718.6 (493.5 ; 992.0)	755.6 (486.2 ; 1051.0)	5.1 (-19.0 ; 35.2)	478.1 (302.8 ; 698.4)	472.2 (285.2 ; 698.0)	-1.2 (-24.1 ; 26.3)
	Smoking	281.5 (206.0 ; 381.2)	259.9 (176.3 ; 362.1)	-7.7 (-39.2 ; 35.4)	1144.0 (886.4 ; 1461.9)	1362.9 (1056.3 ; 1739.2)	19.1 (-11.8 ; 59.9)	693.8 (552.7 ; 858.8)	761.4 (587.0 ; 968.6)	9.7 (-18.3 ; 46.7)
**Portugal**	All risk factors	3975.2 (3678.9 ; 4220.2)	1259.7 (1125.8 ; 1379.5)	-68.3 (-70.5 ; -66.0)	6467.6 (6199.8 ; 6737.6)	2307.9 (2164.6 ; 2458.8)	-64.3 (-66.1 ; -62.4)	5089.2 (4813.4 ; 5329.9)	1742.1 (1601.3 ; 1867.5)	-65.8 (-67.4 ; -63.9)
	Air pollution	383.5 (140.9 ; 684.6)	58.6 ( 34.2 ; 84.3)	-84.7 (-92.1 ; -63.2)	635.5 (214.7 ; 1135.0)	115.6 ( 67.1 ; 168.5)	-81.8 (-90.8 ; -53.6)	498.6 (173.3 ; 889.3)	85.1 ( 49.3 ; 123.3)	-82.9 (-91.3 ; -58.4)
	Alcohol use	156.7 ( 65.6 ; 250.4)	34.5 ( 11.6 ; 59.6)	-78.0 (-87.6 ; -66.2)	817.7 (545.1 ; 1084.0)	244.2 (159.3 ; 335.1)	-70.1 (-75.0 ; -65.3)	434.4 (315.3 ; 557.0)	126.3 ( 85.6 ; 172.2)	-70.9 (-76.6 ; -65.2)
	Dietary risks	1284.8 (1060.2 ; 1551.1)	421.2 (341.8 ; 527.1)	-67.2 (-69.9 ; -63.8)	2418.6 (1986.6 ; 2921.4)	928.3 (751.5 ; 1140.0)	-61.6 (-64.6 ; -58.1)	1799.3 (1477.3 ; 2165.0)	655.6 (535.7 ; 806.8)	-63.6 (-66.1 ; -60.4)
	High body-mass index	826.0 (466.4 ; 1206.6)	327.5 (208.2 ; 459.9)	-60.4 (-65.1 ; -51.2)	1186.0 (563.5 ; 1876.8)	554.2 (305.8 ; 830.1)	-53.3 (-59.7 ; -39.5)	997.8 (527.0 ; 1519.4)	435.5 (255.2 ; 633.1)	-56.4 (-61.4 ; -45.4)
	High fasting plasma glucose	835.3 (539.4 ; 1328.9)	370.1 (253.2 ; 541.8)	-55.7 (-69.3 ; -37.9)	1211.2 (832.5 ; 1830.0)	664.7 (475.1 ; 935.0)	-45.1 (-59.1 ; -28.6)	1005.5 (687.6 ; 1530.6)	503.2 (364.3 ; 711.2)	-50.0 (-61.6 ; -36.2)
	High LDL cholesterol	1128.6 (811.9 ; 1602.0)	346.0 (247.0 ; 476.9)	-69.3 (-72.0 ; -66.3)	2115.0 (1689.9 ; 2725.8)	750.7 (613.5 ; 924.6)	-64.5 (-67.6 ; -61.4)	1579.1 (1216.6 ; 2105.8)	534.5 (422.8 ; 687.7)	-66.1 (-68.9 ; -63.2)
	High systolic blood pressure	2545.2 (2121.7 ; 2988.7)	755.9 (614.3 ; 894.2)	-70.3 (-75.6 ; -64.2)	4088.5 (3569.3 ; 4595.6)	1437.8 (1255.6 ; 1611.7)	-64.8 (-68.7 ; -60.6)	3243.3 (2797.8 ; 3673.3)	1072.5 (938.7 ; 1209.2)	-66.9 (-70.3 ; -62.8)
	Kidney dysfunction	331.4 (255.8 ; 406.1)	107.3 ( 81.9 ; 134.3)	-67.6 (-70.4 ; -64.7)	472.2 (378.5 ; 570.1)	163.3 (129.3 ; 199.9)	-65.4 (-67.8 ; -62.6)	395.9 (312.8 ; 478.4)	133.4 (105.0 ; 162.6)	-66.3 (-68.6 ; -64.0)
	Low physical activity	241.0 ( 96.4 ; 454.2)	79.2 ( 34.1 ; 143.3)	-67.2 (-71.9 ; -59.5)	287.0 ( 86.6 ; 611.0)	105.6 ( 34.2 ; 210.8)	-63.2 (-70.7 ; -52.7)	265.6 ( 98.9 ; 524.9)	92.4 ( 35.7 ; 173.5)	-65.2 (-70.6 ; -58.3)
	Non-optimal temperature	402.4 (331.0 ; 480.2)	111.3 ( 89.0 ; 133.4)	-72.3 (-74.4 ; -70.3)	629.7 (517.6 ; 747.5)	195.3 (158.8 ; 235.2)	-69.0 (-70.9 ; -66.8)	504.2 (414.7 ; 600.5)	150.0 (121.7 ; 179.8)	-70.2 (-72.0 ; -68.3)
	Other environmental risks	163.0 ( 77.1 ; 253.2)	44.3 ( 18.6 ; 72.6)	-72.8 (-77.5 ; -69.2)	439.9 (284.8 ; 596.2)	119.0 ( 67.6 ; 171.9)	-72.9 (-77.6 ; -69.6)	282.9 (169.0 ; 398.2)	76.9 ( 39.9 ; 115.5)	-72.8 (-77.1 ; -69.7)
	Smoking	644.0 (564.1 ; 726.7)	167.6 (147.9 ; 189.1)	-74.0 (-77.6 ; -70.0)	2087.3 (1971.8 ; 2207.3)	638.6 (591.6 ; 685.4)	-69.4 (-71.8 ; -67.0)	1285.1 (1205.3 ; 1361.9)	384.2 (355.8 ; 414.3)	-70.1 (-72.5 ; -67.6)
**Sao Tome and Principe**	All risk factors	4903.8 (4146.2 ; 5592.8)	5285.6 (4171.6 ; 6407.3)	7.8 (-14.3 ; 34.7)	4424.0 (3548.5 ; 5303.5)	5220.0 (4291.5 ; 6097.6)	18.0 ( -7.1 ; 49.6)	4655.3 (3913.5 ; 5399.0)	5262.6 (4320.2 ; 6132.4)	13.0 ( -8.0 ; 39.6)
	Air pollution	1927.4 (1580.0 ; 2328.9)	1612.4 (1214.5 ; 2072.8)	-16.3 (-36.9 ; 11.7)	1749.2 (1367.1 ; 2168.1)	1628.6 (1240.5 ; 2014.2)	-6.9 (-29.8 ; 23.8)	1838.3 (1482.7 ; 2214.2)	1622.8 (1253.5 ; 2017.9)	-11.7 (-32.2 ; 15.9)
	Alcohol use	54.3 (-35.1 ; 156.7)	122.0 ( 0.2 ; 271.2)	124.8 (-1878.3 ; 1478.3)	202.3 ( 87.7 ; 336.6)	317.8 (168.3 ; 499.0)	57.1 (-11.5 ; 224.3)	124.8 ( 38.8 ; 226.5)	216.5 (109.1 ; 349.9)	73.5 (-19.7 ; 372.5)
	Dietary risks	1651.5 (1207.6 ; 2333.8)	1742.7 (1174.3 ; 2546.6)	5.5 (-19.0 ; 34.5)	1720.3 (1250.9 ; 2386.2)	1940.4 (1375.8 ; 2690.4)	12.8 (-11.6 ; 45.3)	1671.0 (1244.1 ; 2325.2)	1840.1 (1297.4 ; 2582.2)	10.1 (-12.4 ; 39.8)
	High body mass index	1149.6 (634.4 ; 1783.9)	1748.3 (1147.1 ; 2490.6)	52.1 ( 8.2 ; 132.7)	610.1 (217.9 ; 1135.0)	1407.7 (866.1 ; 2120.3)	130.7 ( 51.9 ; 367.7)	889.6 (438.9 ; 1451.8)	1584.0 (1035.0 ; 2249.3)	78.0 ( 28.9 ; 180.7)
	High fasting plasma glucose	691.4 (450.7 ; 1043.4)	1163.6 (723.9 ; 1714.4)	68.3 ( 21.0 ; 139.6)	699.6 (424.2 ; 1103.9)	1239.2 (795.4 ; 1801.9)	77.1 ( 28.4 ; 151.8)	685.2 (451.9 ; 1025.6)	1198.8 (779.4 ; 1699.3)	75.0 ( 33.9 ; 136.3)
	High LDL cholesterol	888.6 (640.0 ; 1165.8)	1140.1 (795.8 ; 1498.6)	28.3 ( -1.5 ; 65.5)	869.3 (588.7 ; 1186.0)	1198.7 (848.3 ; 1572.6)	37.9 ( 7.1 ; 79.1)	874.7 (622.5 ; 1152.5)	1171.4 (844.4 ; 1517.1)	33.9 ( 6.0 ; 69.4)
	High systolic blood pressure	3539.8 (2811.3 ; 4228.3)	3934.8 (3001.7 ; 4860.0)	11.2 (-14.1 ; 44.8)	3045.7 (2333.9 ; 3761.0)	3759.9 (2962.7 ; 4521.8)	23.5 ( -4.0 ; 60.7)	3297.0 (2654.7 ; 3911.1)	3859.2 (3065.5 ; 4637.7)	17.0 ( -6.3 ; 47.6)
	Kidney dysfunction	393.4 (304.1 ; 483.5)	556.2 (418.5 ; 716.7)	41.4 ( 12.3 ; 81.0)	290.2 (209.9 ; 388.4)	437.6 (323.3 ; 566.3)	50.8 ( 20.5 ; 90.3)	343.4 (259.3 ; 432.0)	500.6 (379.5 ; 629.2)	45.8 ( 19.3 ; 82.2)
	Low physical activity	97.0 ( 36.6 ; 212.3)	129.9 ( 51.2 ; 274.9)	33.9 ( 5.2 ; 72.7)	79.8 ( 22.8 ; 194.3)	109.0 ( 31.4 ; 268.5)	36.7 ( 4.9 ; 78.2)	88.6 ( 30.7 ; 202.8)	120.6 ( 41.9 ; 276.0)	36.1 ( 9.4 ; 69.8)
	Non-optimal temperature	16.3 (-55.9 ; 81.4)	21.7 (-12.1 ; 84.2)	33.3 (-278.6 ; 367.3)	15.4 (-47.6 ; 78.9)	22.3 (-13.0 ; 81.8)	45.4 (-384.5 ; 329.1)	15.8 (-53.4 ; 78.8)	22.0 (-13.1 ; 82.7)	39.9 (-334.0 ; 336.1)
	Other environmental risks	159.0 ( 42.7 ; 283.2)	155.8 ( 45.4 ; 289.4)	-2.0 (-24.1 ; 28.9)	200.0 ( 88.9 ; 325.3)	210.9 ( 96.8 ; 347.0)	5.5 (-18.7 ; 34.9)	177.1 ( 66.0 ; 300.8)	182.2 ( 70.6 ; 311.0)	2.9 (-17.5 ; 28.5)
	Smoking	154.4 (119.1 ; 196.4)	173.9 (126.4 ; 230.7)	12.6 (-19.9 ; 64.0)	470.5 (354.8 ; 599.5)	615.5 (474.0 ; 768.4)	30.8 ( -1.8 ; 78.6)	306.2 (237.3 ; 377.2)	389.8 (300.0 ; 482.8)	27.3 ( -3.4 ; 72.3)
**Timor-Leste**	All risk factors	5245.2 (4237.9 ; 6318.1)	5727.3 (4650.7 ; 6805.8)	9.2 (-16.5 ; 38.6)	5157.0 (3969.3 ; 7028.7)	7221.6 (5334.1 ; 9597.5)	40.0 ( 2.1 ; 77.9)	5205.8 (4272.1 ; 6425.2)	6476.7 (5147.2 ; 7999.9)	24.4 ( -5.1 ; 54.9)
	Air pollution	2196.8 (1686.5 ; 2845.8)	1967.9 (1518.9 ; 2435.5)	-10.4 (-34.7 ; 19.2)	2080.5 (1505.1 ; 2932.2)	2410.1 (1650.4 ; 3329.1)	15.8 (-19.6 ; 51.9)	2140.0 (1646.1 ; 2762.8)	2190.6 (1631.0 ; 2828.2)	2.4 (-25.5 ; 33.2)
	Alcohol use	-2.7 (-13.5 ; 10.5)	11.9 (-16.3 ; 50.8)	-547.6 (-3649.5 ; 3536.9)	39.0 (-49.8 ; 142.4)	295.6 ( 57.7 ; 583.8)	657.3 (-5045.9 ; 6221.4)	18.9 (-28.1 ; 74.7)	154.3 ( 28.3 ; 318.1)	717.6 (-5256.3 ; 9199.6)
	Dietary risks	2380.6 (1638.0 ; 3255.7)	2418.4 (1677.1 ; 3291.1)	1.6 (-24.7 ; 31.4)	2645.0 (1804.2 ; 3825.0)	3452.2 (2270.2 ; 4875.1)	30.5 ( -6.9 ; 71.7)	2516.4 (1794.0 ; 3435.5)	2936.2 (2011.2 ; 4031.7)	16.7 (-13.8 ; 51.1)
	High body-mass index	380.3 ( 96.7 ; 847.5)	590.8 (211.4 ; 1136.1)	55.4 ( 0.9 ; 210.6)	254.1 ( 43.6 ; 675.6)	668.8 (212.5 ; 1339.0)	163.2 ( 55.4 ; 577.8)	315.6 ( 71.8 ; 766.0)	630.9 (223.9 ; 1210.9)	99.9 ( 30.9 ; 303.3)
	High fasting plasma glucose	543.6 (359.3 ; 818.3)	1493.1 (986.7 ; 2191.2)	174.7 ( 94.2 ; 292.1)	597.0 (359.7 ; 941.4)	1832.7 (1179.3 ; 2821.4)	207.0 (110.8 ; 347.6)	569.1 (370.6 ; 852.3)	1659.8 (1121.0 ; 2421.9)	191.6 (113.4 ; 300.2)
	High LDL cholesterol	994.8 (710.9 ; 1343.8)	1180.1 (820.5 ; 1595.4)	18.6 (-12.4 ; 55.0)	976.6 (652.3 ; 1471.2)	1447.2 (885.4 ; 2125.4)	48.2 ( 1.6 ; 97.6)	988.2 (704.4 ; 1351.8)	1317.3 (894.6 ; 1800.3)	33.3 ( -4.0 ; 73.7)
	High systolic blood pressure	3595.3 (2758.9 ; 4466.5)	3782.6 (2933.5 ; 4685.8)	5.2 (-24.2 ; 39.2)	3442.5 (2548.8 ; 4786.6)	5095.6 (3629.2 ; 6895.6)	48.0 ( 5.8 ; 92.4)	3523.1 (2794.6 ; 4533.6)	4442.1 (3386.4 ; 5642.3)	26.1 ( -6.5 ; 61.4)
	Kidney dysfunction	502.7 (370.5 ; 653.6)	685.8 (504.1 ; 882.6)	36.4 ( 2.4 ; 75.0)	446.5 (305.9 ; 651.6)	761.5 (504.6 ; 1077.3)	70.5 ( 23.6 ; 121.4)	474.9 (343.5 ; 633.8)	724.4 (519.6 ; 965.7)	52.5 ( 15.9 ; 93.5)
	Low physical activity	83.0 ( 29.5 ; 195.3)	102.4 ( 34.9 ; 241.0)	23.4 ( -7.2 ; 61.0)	93.5 ( 26.9 ; 217.3)	138.8 ( 42.0 ; 334.3)	48.5 ( 9.8 ; 91.8)	88.3 ( 28.9 ; 204.7)	120.4 ( 39.1 ; 285.0)	36.3 ( 7.3 ; 68.7)
	Non-optimal temperature	76.8 ( 12.6 ; 147.1)	73.8 ( 24.3 ; 141.0)	-3.9 (-49.4 ; 92.4)	75.7 ( 12.9 ; 149.9)	94.1 ( 30.3 ; 188.7)	24.3 (-35.6 ; 148.0)	76.4 ( 13.7 ; 146.5)	84.0 ( 27.0 ; 164.3)	10.0 (-40.5 ; 113.1)
	Other environmental risks	181.8 ( 50.0 ; 334.9)	199.7 ( 70.9 ; 355.6)	9.8 (-15.7 ; 55.3)	289.1 (141.3 ; 480.4)	384.0 (193.6 ; 622.3)	32.8 ( -3.9 ; 73.2)	236.2 ( 97.4 ; 394.6)	291.8 (131.7 ; 468.7)	23.6 ( -6.1 ; 59.4)
	Smoking	607.6 (445.7 ; 793.9)	545.8 (400.2 ; 711.8)	-10.2 (-36.7 ; 24.1)	1775.7 (1295.2 ; 2464.1)	2338.8 (1659.3 ; 3187.1)	31.7 ( -9.1 ; 76.7)	1203.3 (923.6 ; 1583.0)	1445.2 (1053.0 ; 1920.8)	20.1 (-14.0 ; 59.0)

LDL: low-density lipoprotein cholesterol.

Figure 2 shows the percentage of CVD deaths attributable to each cardiovascular risk factor, by country, in 1990 and 2019. High SBP remained with the highest percentage, with even an increase observed in all PSC except Portugal. Still, it was noted an overall increase in the contribution of dietary risks between 1990 and 2019 (3^rd^ to 2^nd^) as well as in alcohol consumption, high fasting plasma glucose and high BMI. Conversely, there was a percent reduction of high LDL cholesterol and, especially, smoking, although the latter still has a more significant contribution in Portugal, Brazil and East Timor.

**Figure 2:** Percentage of total cardiovascular disease deaths attributable to each cardiovascular risk factor, by Portuguese-speaking country in 1990 and 2019.

In the analysis of CVD mortality rates attributable to selected RF by PSC (Figure 3), it was observed that high SBP ranked first in all PSC in 1990 and 2019. In 1990, CVD mortality rates per 100,000 inhabitants attributed to PAS were highest in Equatorial Guinea (253.5), Angola (225.3) and Guinea Bissau (214.0), while in 2019, these rates were highest in Mozambique (224.1), Guinea Bissau (220.4) and East Timor (210.4), with the most significant reductions observed in Portugal (-66.6%, 95% UI -71.0 - 61.2%) and Brazil (-49.8%, 95% UI -52.5 - -47.1%). Dietary risks, high fasting plasma glucose, high LDL-cholesterol and air pollution were among the top five RF in most PSC in 1990 and 2019, except for the markedly lower rates attributable to air pollution in Brazil and Portugal both in 1990 and in 2019, also with a more significant reduction in these countries in the period. The increase in mortality rates attributable to alcohol consumption in nearly all PSC, except for Brazil and Portugal, and the reduction in smoking (also notably in Brazil [-69.5%] and Portugal [-73.2%]) were also remarkable, despite the still relatively higher rates in these two countries and in East Timor in 2019 (Figure 3, Table 1). Table 2 shows similar patterns for DALYs attributable to RF in the PSC.

**Figure 3:** Age-standardized cardiovascular disease mortality rate (/100,000 inhabitants) attributable to cardiovascular risk factors, by Portuguese-speaking country, in 1990 and 2019.

Assessing CVD mortality rate and DALYs attributable to the combined cardiovascular RF, Figure 4 shows a trend towards stability between 1990 – 2019 in most PSC for crude rates, with a decreasing trend in Portugal and Equatorial Guinea, and an increase in East Timor. For age-standardized rates, Portugal and Brazil had a strong downward trend, contrasting with the other countries, which showed a pattern of relative stability, or increase in the case of Mozambique and East Timor (PSC at the lower limits of the SDI).

**Figure 4:** Cardiovascular disease mortality ate and disability-adjusted life years (DALYs) attributable to grouped cardiovascular risk factors in Portuguese-speaking countries, between 1990 and 2019.

Figure 5 shows the percent changes in mortality rates attributable to selected FR according to SDI in 2019 for each PSC. For all RF, an inverse correlation trend between SDI and percent change was observed, with statistical significance for dietary risks, high LDL cholesterol and high SBP. In the three PSC with the highest SDI (Portugal, Equatorial Guinea and Brazil) there was a considerable reduction in mortality rates attributable to all RF, except for high fasting glucose and high BMI, which tended, respectively, towards stability and increase only in Equatorial Guinea. For age-standardized SEV rates (Figure S4), the observed pattern was different, with a trend towards a negative correlation between the percent change in SEV rates attributable to smoking and SDI, with a significant positive correlation observed only for dietary factors.

**Figure 5:** Correlation between the sociodemographic index (SDI) and the percent change in age-standardized cardiovascular disease mortality rates attributable to selected risk factors in Portuguese-speaking countries from 1990 to 2019.

## Discussion

The PSC share sociocultural characteristics derived from the common Portuguese colonization, albeit in different degrees, often coexisting with traces of other cultures that participated in the colonization process and population composition. There are approximately 280 million Portuguese speakers in the world (approximately 216 million in Brazil); Portuguese is the fifth most spoken language in the world, and the most spoken in the southern hemisphere.^3^ PSC have different socioeconomic realities and health system inequality, but similar ethnicities, which are known determining factors for CVD.^3,6,7^ Our analysis of cardiovascular RF in PSC reinforces this heterogeneity, demonstrating a more significant reduction in CVD attributable to RF in countries with more structured health systems, and a close relationship between mortality trends and SDI, especially for dietary factors, high LDL cholesterol and elevated SBP.

A cross-sectional retrospective study of outpatients born in Portugal, Brazil and Africa, among others, from general practice clinics in Lambeth, South London, found that Portuguese speakers (the largest non-English language preference group) were more likely to have hypertension (OR=1.43, 95% CI 1.30 - 1.57); diabetes mellitus (OR=1.74, 95% CI=1.50 - 2.02); stroke (OR = 1.40, 95% CI = 1.08-1.81); obesity (OR=1.53, 95% CI=1.36-1.73); and smoking (OR=1.13, 95% CI = 1.02 to 1.25) compared to other ethnic groups. The authors discussed whether these differences could be explained by language barriers, or if they would derive from common genetic determinants, in addition to – above all – social and cultural factors.^19^

Our study found that CVD attributable to cardiovascular RF accounted for approximately 30% of total deaths in most PSC in 2019, although in countries with SDI less than 0.5, this percentage was less than 15%, except for Guinea Equatorial (0.69) which had the second highest SDI among all PSC. This trend is associated with a late epidemiological transition, *i.e*., countries with the worst socioeconomic markers still tend to present increased proportions of these diseases and can, therefore, apply successful strategies previously applied to other PSC, trying to mitigate this trend.^3^ The percentage attributable to RF was high (>75%) in all PSC, and high SBP was the main risk factor for CVDs in the period analyzed. In all PSC, there was a reduction in the age-standardized CVD mortality rates attributed to RF in the period, especially in countries with higher SDI (Portugal, Equatorial Guinea and Brazil). It is noteworthy that Equatorial Guinea has the largest *per capita* gross domestic product in the African continent, however, resources are unevenly distributed, with little benefit to the general population and coexisting proportional mortality from chronic and infectious diseases.^6^

Hypertension, diabetes mellitus, hypercholesterolemia, obesity, and smoking were the top five modifiable traditional cardiovascular RF observed in Africa in 2019.^4^ At least one of these five RF is present in 80% to 95% of individuals who suffer a fatal or non-fatal cardiovascular event in the continent.^4,20^ The same was observed for Brazil and Portugal,^3,21^ except for smoking, which showed significant reductions in these countries during this period, as previously reported in another study,^4^ as a result of public policies and coping campaigns.^4^ On the other hand, an increase in mortality rates from CVDs attributable to alcohol consumption was observed in the PSC of Africa and in Equatorial Guinea and Guinea Bissau, probably reflecting the worldwide trend of increasing alcohol consumption with an impact on CVDs.^22^

It is noteworthy that CVD mortality rates attributable to high SBP remained first in the ranking in all PSC between 1990 and 2019. Again, more expressive reductions were observed in Portugal and Brazil,^3,21^ probably associated with the highest SDIs, but also with population policies to reduce salt intake, especially in Portugal, where there was a reduction in myocardial infarction and stroke attributable to high SBP.^21^ These data show a change in the profile of countries with higher CVD mortality rates attributable to high SBP, with a decline in those with better socioeconomic indices and earlier epidemiological transition, with an inverse trend in those with lower SDIs.

The PSC showed an increase in dietary and metabolic risk factors attributable to CVD mortality. The *Prospective Urban Rural Epidemiology* (PURE) study, conducted in 21 countries, with 148,858 participants and a mean follow-up of 9.5 years, showed that higher intakes of refined grains, which accounted for 70% of caloric intake in African countries, were associated with higher SBP, and higher risk of all-cause and CVD mortality.^23^ In our analysis, dietary risks associated with high fasting plasma glucose and high LDL-cholesterol were ranked among the top five RF in most PSC in 1990 and 2019, and correlated with PSC with lower SDIs. These findings were also observed in a GBD substudy that analyzed mortality and disease burden associated with CVD worldwide.^2^

A study that analyzed the burden of CVD in 194 countries around the world, between 1990 and 2019, showed a downward trend in the DALYs, YLL and YLD, with higher rates of YLD in women compared to men,^24^ with the same occurring in relation to the burden of CVDs attributable to cardiovascular RF.^4^ These data highlight the heterogeneity of PSC in terms of mortality and disease burden, in which variations cannot be explained by the SDI alone, with a potential contribution of multiple factors such as gender, ethnicity and even cultural and environmental differences.

The PURE study suggested that a large proportion of premature CVD deaths could be avoided by decreasing some modifiable RF with global policies such as controlling hypertension and smoking and improving health education.^25^ The impact of reducing others RF such as dietary risks and ambient air pollution may vary according to the socioeconomic level of each country, and with the development of internal regulations of economic activities (such as the emission of pollutants and rapairing of environmental damage).^26,27^ Thus, strategies to address CVD mortality and burden in PSC could focus, at this time, on the most prevalent RF, with low-cost and high-impact population measures, such as reducing dietary intake of salt and calories, reducing tobacco and alcohol consumption, and blood pressure control.

### Limitations and strengths of the study

Limitations related to the methodology of the GBD study have been previously detailed,^4,7^ and are mainly related to the heterogeneity of primary data sources among the PSC, completeness of mortality statistics and limitations of extrapolating estimates for territories with low data quality – a condition observed for some PSC. There has been a progressive improvement in the completeness of prevalence and morbidity data; however, integrity and quality for some PSC are still limited, according to the GBD 2019.^4^ As an example, there are very low rates or non-existent data on mortality in PSC in sub-Saharan Africa.^7,8^ It is possible that there was also an inadequacy of the GBD study models for different countries in some groups of diseases subject to less epidemiological surveillance, especially non-notifiable cardiovascular RF. Furthermore, for some RF, there are no surveys or specific health surveillance programs in several PSC. Specifically regarding RF estimates, the GBD 2019 assumes uniform distribution of RR in all countries, for the same age and sex.^4^ which may potentially increase uncertainty of the results. Primary studies, where available, report prevalence data as a measure of exposure to a risk factor, which limits comparability with risk exposure measures (SEV) of the GBD. Furthermore, the GBD methodology disregards distal RF, which can mediate the prevalence and mortality of intermediate RF, affecting their effects as social determinants of health.^28,29^ Another methodological aspect is the limitation of modeling for the coexistence of simultaneous RF, which is known to result in a risk greater than the sum of individual factors (e.g., hypertension, in the presence of diabetes and smoking, potentiating ischemic heart disease).^2,30^ Additionally, the adjustment method of RF for standardized definitions applied by GBD can be an additional source of bias.^4,15^ Finally, despite similar colonization, the sociocultural, demographic, economic and ethnic heterogeneity of the PSC – influencing lifestyle habits, health behaviors, awareness and control of RF – may not be adequately captured by the analytical models.^6^

However, despite these limitations, GBD is a robust, comprehensive and validated methodology, from the epidemiological point of view, for estimating the burden of disease attributable to cardiovascular RF, through the production of comparable metrics between PSC – including those with scarce or no primary data. In addition, in light of the reality of local health systems, our data may help the reformulation of health policies.

## Conclusions

The set of 12 cardiovascular RFs included in this analysis of GBD 2019 account for over 75% of the CVD burden in the nine PSC, with a greater impact of these diseases on mortality in Portugal, East Timor, Cape Verde and Brazil. High SBP remained as the main risk factor for cardiovascular mortality and DALYs between 1990 and 2019. There was a significant reduction in age-standardized cardiovascular mortality rates attributable to RF, especially in the PSC with better socioeconomic indices, such as Brazil, Portugal and Equatorial Guinea. Overall, there has been an increasing impact of dietary and metabolic RF, in parallel with reduced rates of tobacco smoking in most PSC. In addition, there was a marked negative correlation between the variation in cardiovascular mortality rates attributable to RF and SDI. These results show the heterogeneity among the PSC in relation to the epidemiology of the RF evaluated, suggesting the need for health policies and government actions adapted to the reality of each country, and for a collaboration between these nations to reduce the impact of CVD.

These data can help countries to identify common problems, being an important stimulus for the exchange of experiences between scientists and academic communities. The PSC must make progress in the engagement and solidarity between them,^31^ especially those with more resources and technical capabilities, supporting the training of human resources and the development of partnerships.

## *Supplemental Materials

For additional information, please click here.
